# The Synthesis and Biological Evaluation of 2-(1*H*-Indol-3-yl)quinazolin-4(3*H*)-One Derivatives

**DOI:** 10.3390/molecules28145348

**Published:** 2023-07-11

**Authors:** Elena Y. Mendogralo, Larisa Y. Nesterova, Ekaterina R. Nasibullina, Roman O. Shcherbakov, Alexander G. Tkachenko, Roman Y. Sidorov, Maxim A. Sukonnikov, Dmitry A. Skvortsov, Maxim G. Uchuskin

**Affiliations:** 1 Department of Chemistry, Perm State University, Bukireva St. 15, 614990 Perm, Russia; larisa.nesterova@bk.ru (L.Y.N.); kat.nasibullina@yandex.ru (E.R.N.); romanshcherbakov00@gmail.com (R.O.S.); agtkachenko@iegm.ru (A.G.T.); sidorov.r@iegm.ru (R.Y.S.); mu@psu.ru (M.G.U.); 2 Institute of Ecology and Genetics of Microorganisms, Ural Branch of the Russian Academy of Sciences, Goleva St. 13, 614081 Perm, Russia; 3 Department of Chemistry, M.V. Lomonosov Moscow State University, Leninskie Gory 1-3, 119991 Moscow, Russia; sukonnikoff.maxim@yandex.ru (M.A.S.); skvorratd@mail.ru (D.A.S.)

**Keywords:** azaheterocycle, indole, quinazolinone, antibacterial activity, resistance, *Mycobacterium smegmatis*, molecular docking

## Abstract

The treatment of many bacterial diseases remains a significant problem due to the increasing antibiotic resistance of their infectious agents. Among others, this is related to *Staphylococcus aureus*, especially methicillin-resistant *S. aureus* (MRSA) and *Mycobacterium tuberculosis*. In the present article, we report on antibacterial compounds with activity against both *S. aureus* and MRSA. A straightforward approach to 2-(1*H*-indol-3-yl)quinazolin-4(3*H*)-one and their analogues was developed. Their structural and functional relationships were also considered. The antimicrobial activity of the synthesized compounds against *Mycobacterium tuberculosis* H_37_Rv, *S. aureus* ATCC 25923, MRSA ATCC 43300, *Candida albicans* ATCC 10231, and their role in the inhibition of the biofilm formation of *S. aureus* were reported. 2-(5-Iodo-1*H*-indol-3-yl)quinazolin-4(3*H*)-one (**3k**) showed a low minimum inhibitory concentration (MIC) of 0.98 μg/mL against MRSA. The synthesized compounds were assessed via molecular docking for their ability to bind long RSH (RelA/SpoT homolog) proteins using mycobacterial and streptococcal (p)ppGpp synthetase structures as models. The cytotoxic activity of some synthesized compounds was studied. Compounds **3c**, **f**, **g**, **k**, **r**, and 3**z** displayed significant antiproliferative activities against all the cancer cell lines tested. Indolylquinazolinones **3b**, **3e**, and **3g** showed a preferential suppression of the growth of rapidly dividing A549 cells compared to slower growing fibroblasts of non-tumor etiology.

## 1. Introduction

One of the most serious threats to human health is bacterial antibiotic resistance [1]. This problem is projected to cause 10 million deaths worldwide each year by 2050 without the development of new strategies for defeating antibiotic resistance [2]. The most commonly isolated nosocomial pathogens are *Escherichia coli* and *Staphylococcus aureus*. Of particular concern currently is methicillin-resistant *S. aureus* (MRSA), which is considered to be one of the most dangerous antibiotic-resistant microorganisms [3]. Another cause of a serious bacterial infection is the bacillus *Mycobacterium tuberculosis*. Until the outbreak of SARS-CoV-2, tuberculosis (TB) was the leading cause of death from a single infectious agent, ranking above HIV/AIDS. It is estimated that, today, about a quarter of the world’s population is infected with TB. Most people do not develop tuberculosis, and in some, the infection clears. However, without treatment, the death rate from TB disease is high (about 50%) [4]. Despite the availability of drugs for the treatment of tuberculosis, there is a need for improved therapeutic agents. Side effects, the duration of treatment, and drug resistance are the main limitations to the effectiveness of the drugs used. One of the factors affecting the resistance of some infections to common antibiotics is the role of bacterial biofilm formation. Biofilms are highly organized surface-associated communities enclosed in an extracellular polymer matrix. The bacteria in a biofilm are known to be 1000 times more resistant to antibiotics. Therefore, one of the attractive approaches to combating multidrug-resistant bacteria is the inhibition of biofilm formation.

**Figure 1:**
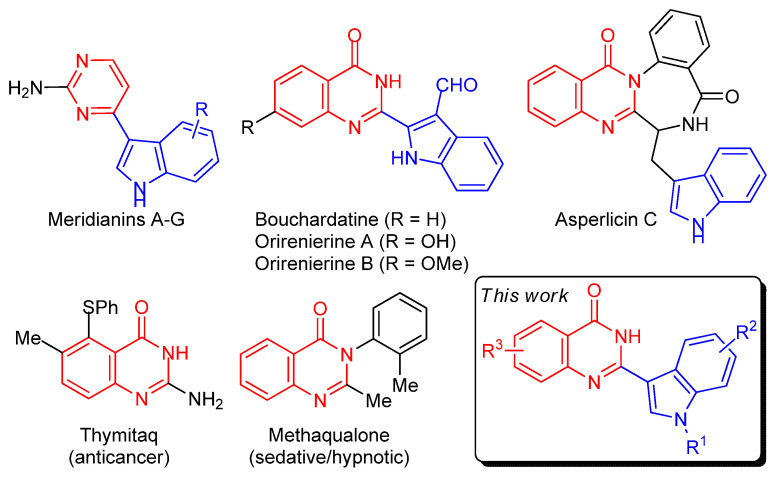
Selected examples of biological active quinazolinone derivatives.

In recent years, many biologically active compounds have been isolated from representatives of marine flora and fauna. Meridianins, a group of indole alkaloids isolated from the tunicate *Aplidium meridianum* [5,6], have promising anti-tuberculosis [7], antimalarial [8,9], and antitumor [10] activities. Notably, analogues of meridianin have shown powerful inhibitory activity against biofilm formation by *S. aureus* (MRSA) [11] and *Mycobacterium smegmatis* [12]. We posited that the closest structural analogues of meridianins would have the ability to control the behavior of bacteria based on the shared structural features of meridianins with quinazolinone derivatives. Quinazolinone scaffolds are among more than 150 natural alkaloids and pharmaceuticals. Quinazolinone derivatives exhibit a wide range of biological activity, with anti-inflammatory, antitumor, antipsychotic, and antibacterial activities (Figure 1) [13,14,15,16,17,18].

The different physiological processes underlying bacterial stress adaptation are under the control of indoles [19]. A family of widely distributed bacterial metabolites, termed the ‘‘indolokines”, are able to enhance *E. coli* persister cell formation, thereby connecting some processes, including biofilm formation, antibiotic tolerance, virulence, and others [20,21]. One of the main regulators of cell adaptive responses to stress is guanosine tetraphosphate (ppGpp), which is a bacterial alarmone participating in the dormant state formation. In this state, bacterial cells are transformed into persister cells that are tolerant to antibiotics and stresses [22]. One of the regulatory effects of ppGpp is the induction of the activity of the ribosome modulation factors that are responsible for the inhibition of protein synthesis. At the same time, it has been shown that indole is also capable of inducing one of the ribosome hibernation factors—RaiA—thereby taking part in persister cell formation [23]. We showed that ppGpp can stimulate tryptophanase induction, which is responsible for indole synthesis, thereby strengthening its own ability to form persisters [24]. Previously, we described the inhibition of alarmone synthetase enzymes, the product of which is ppGpp [25]. In turn, the inhibition of these enzymes suppressed the ability of bacterial cells to form a persister state responsible for antibiotic tolerance. Furthermore, it was shown that substituted indoles are able to bind Rel_Seq_ (p)ppGpp synthetase/hydrolase and can constitute a promising scaffold for (p)ppGpp synthetase enzyme inhibitors’ development [26].

Based on data from the literature, we hypothesized that the combination of a quinazolinone fragment with an additional azaheterocycle in a single molecule could promise a synergistic enhancement of the pharmaceutical value of these two biologically important structural subunits. A wide library of synthesized indole derivatives will make it possible to find among them those that are able to bind alarmone synthetases as the probable targets for interaction. To date, many methods have been developed for the synthesis of quinazolinones and their analogues due to their high applied significance [27]. The most common synthetic approach to quinazolin-4(3*H*)-ones involves the condensation of anthranilamides with aromatic aldehydes [28,29,30,31,32], ketones [33,34], alkynes [35], carboxylic acid [36], and their derivatives [37], benzyl alcohols [38], CO/aryl bromides [39], and methylarenes [40], etc. While approaches to quinazolin-4(3*H*)-ones are well known and capable of providing the formation of the desired framework, they suffer from certain drawbacks. Unfortunately, these methods require expensive transition metal catalysts, additives, harsh reaction conditions, and a moderate reaction scale. The most significant disadvantage of most of the described methods is associated with the limited applicability of the starting heteroaromatic substrates. Therefore, the search for a simple and efficient protocol for accessing 2-(1*H*-indol-3-yl)quinazolin-4(3*H*)-one from readily available starting substrates under simple reaction conditions is highly desirable.

Herein, we describe a straightforward approach to 2-(1*H*-indol-3-yl)quinazolin-4(3*H*)-one and their analogues based on the condensation of anthranilamides with aldehydes and an investigation of their cytotoxicity and antibacterial activity, including tests against *Mycobacterium tuberculosis* H_37_Rv, as well as their influence on the formation of biofilms. We describe the results of molecular docking studies on the ability of substituted indoles to bind to long RSH proteins, using two proteins functioning as the alarmone synthetases: Rel_Mtb_ from *Mycobacterium tuberculosis* [41] and Rel_Seq_ from *Streptococcus equisimilis* as the models [26].

## 2. Results and Discussion

### 2.1. Chemistry

**Scheme 1:**
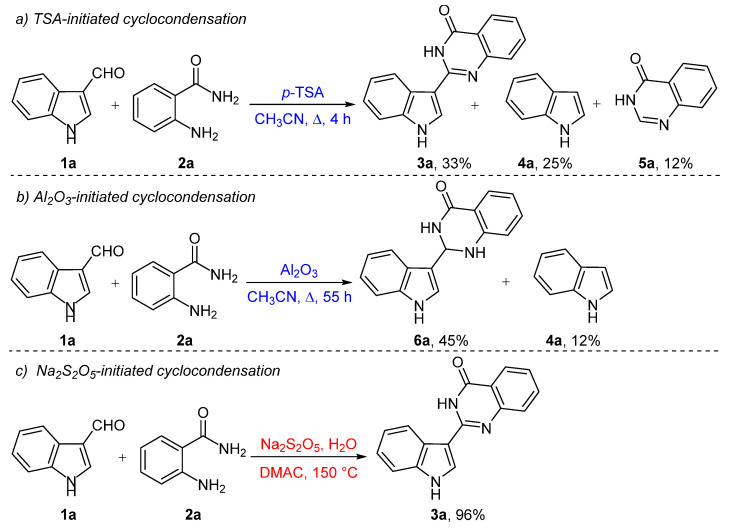
The key results of optimization of conditions for the model cyclocondensation reaction.

The starting point of our research was the search for the optimal conditions of the model reaction of commercially available 1*H*-indole-3-carboxaldehyde (**1a**) with anthranilamide (**2a**). Initially, we screened the reaction conditions using various Brønsted acids. The best result was achieved when the reaction mixture was stirred at reflux for 4 h in acetonitrile in the presence of *p*-TSA (*p*-toluenesulfonic acid). In this case, the yield of desired quinazolinone **3a** was 33% (Scheme 1a). A thorough analysis and separation of the reaction mixture revealed the formation of indole (**4a**) and unsubstituted quinazolinone **5a**. In other acidic conditions (amberlyst-15, CH_3_CN or toluene; NH_4_Cl or AcOH, EtOH; citric acid, toluene, etc.), various side processes were observed, which significantly decreased the yield of the desired product. Next, we found that changing the reaction initiator to Al_2_O_3_ led to the formation of 2-(1*H*-indol-3-yl)-2,3-dihydroquinazolin-4(1*H*)-one (**6a**) as a major product (Scheme 1b). In addition to dihydroquinazolinone **6a**, some indole (**4a**) was isolated from the reaction mixture. The obtained results (Scheme 1a,b) indicate that the key intermediate quinazolinone **6a** is unstable under used reaction conditions and can be oxidized to form the desired quinazolinone **3a** or undergo degradation affording 3-unsubstituted indole (**4a**). Control experiments showed that the dihydroquinazolinone **6a** in the EtOAc, CH_2_Cl_2_, and ethanol solutions was oxidized to key quinazolinone **3a** upon standing in air. Moreover, the deformylation reaction and its related cleavage processes are well known and widely used as a key step for the preparation of natural and synthetic compounds [42,43,44,45]. Finally, the desired product **3a** was obtained in a quantitative yield when the reaction was performed in *N*,*N*-dimethylacetamide (DMAC) in the presence of Na_2_S_2_O_5_ at 150 °C (Scheme 1c). Presumably, the true initiator of the cyclocondensation was NaHSO_3_, which was in situ generated via the hydrolysis of the starting Na_2_S_2_O_5_ [46].

**Scheme 2:**
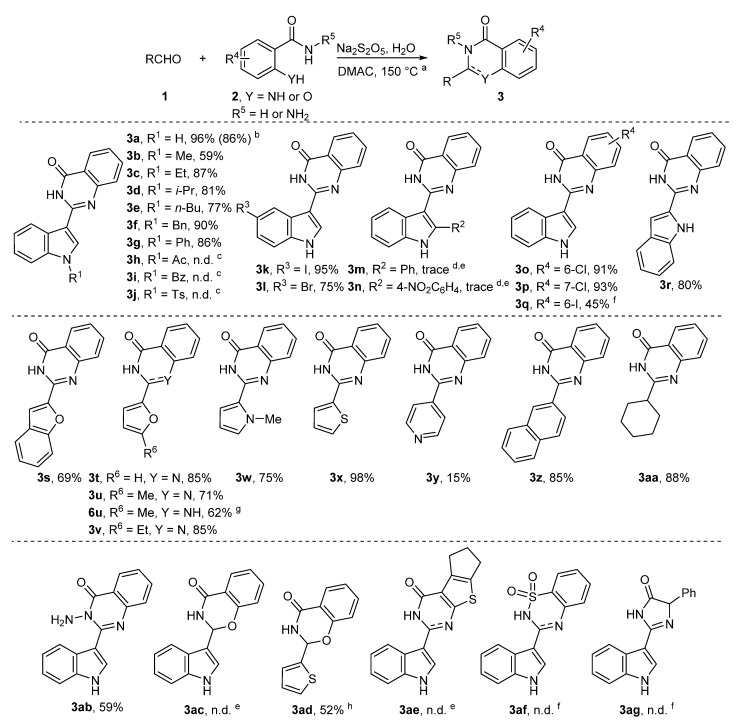
The synthesis of substituted indolylquinazolinones **3**. ^a^ All reactions were performed at 1.3 mmol scale of aldehyde **1** and 1.3 mmol of amide **2**. Isolated yields. ^b^ The reaction was performed at 3 mmol of aldehyde **1** and 3 mmol of amide **2**. Isolated yield. ^c^ The 2-(1*H*-indol-3-yl)quinazolin-4(3*H*)-one (**3a**) was isolated in 80–96% yield (n.d.—not detected). ^d^ The formation of indolylquinazolinones **3m**, **n** were detected using thin-layer chromatography (TLC) and gas chromatography–mass spectrometry (GC/MS). The quinazolinone **5a** was isolated in 37% and 59% yields. ^e^ The formation of indole (**4a**) was determined using TLC and GC/MS. ^f^ The corresponding *N*,*N*-dimethyl-1*H*-indole-3-carbothioamide (**7a**) was also isolated in a 21–50% yield. ^g^ The reaction conditions: aldehyde **1** (0.76 mmol), amide **2** (0.69 mmol), DMSO (1 mL), 100 °C, 3.5 h. Isolated yield. ^h^ The reaction conditions: aldehyde **1** (0.25 mmol), amide **2** (0.25 mmol), TFA (0.25 mmol), toluene (2 mL), reflux, 40 h.

With the optimized conditions in hand, we examined the substrate scope of this reaction. Initially, we performed the reaction at a 3 mmol scale and found that the yield of the desired product **3a** was 86% (Scheme 2). Next, we found that anthranilamide (**2a**) reacted smoothly with various *N*-substituted 3-formylindoles **1** to form the corresponding products **3b**-**g** in good to excellent yields. When indole-3-carboxaldehydes **1** containing easily removal groups at the nitrogen atom were used, we isolated the indolylquinazolinone **3a** instead of the expected *N*-substituted indolylquinazolinones **3h**–**j**. The products **3m**, **n,** containing an aromatic substituent at the C(2) position of the indole core, were detected only in trace amounts, while quinazolinone **5a** was isolated in 59 and 37% yields, respectively. The formation of quinazolinone **5a** in these cases could be associated with the better leaving nature of the 2-arylindoles in the intermediate dihydroquinazolinones. The halogen atoms in the starting indole **1** or anthranilamide **2** did not have a significant influence on the yields of the desired products **3k**, **l**, **o**, and 3**p**. A reduced yield of indolylquinazolinone **3q** was associated with the partial thioamidation of the 1*H*-indole-3-carboxaldehyde (**1a**) by elemental sulfur upon the prolonged heating of the reaction mixture [47]; among the major indolylquinazolinone **3q**, we isolated *N*,*N*-dimethyl-1*H*-indole-3-carbothioamide (**7a**) in a 21% yield (for details see Supplementary Materials). We also performed the reaction of indole-2-carboxaldehyde (**1o**) with anthranilamide (**2a**) and isolated the corresponding product **3r** in a high yield.

Next, we expanded the scope of the developed protocol and used various carbo- and heteroaromatic aldehydes **1** as starting substrates. We found that, in all cases, the desired indolylquinazolinones **3** were isolated in high yields. The exception was the electron-deficient isonicotinaldehyde, for which the desired product **3y** was obtained in a low yield. It should be noted that the use of dimethyl sulfoxide without additional reagents in the reaction of 5-methylfurfural with anthranilamide (**2a**) resulted in the formation of the dihydroquinazolinone **6u** in a 62% yield. Product **6u**, in contrast to its indole analogue **6a**, was more stable and was not oxidized in the solution in air atmosphere.

We tested a series of structural analogues of anthranilamide with 1*H*-indole-3-carboxaldehyde (**1a**). We found that the reaction of aldehyde **1a** with anthranilic acid hydrazide led to the corresponding product **3ab** in a good yield. The reaction of salicylic amide with 1*H*-indole-3-carboxaldehyde (**1a**) unfortunately did not provide the desired product **3ac** due to the predominant occurrence of the deformylation reaction. A similar result was also obtained in the case of Gewald’s thiophene. It is noteworthy that, when using thiophene-2-carbaldehyde, the product **3ad** was isolated in a moderate yield. In the case of 2-aminobenzenesulfonamide and 2-amino-2-phenylacetamide, the thioamidation reaction predominated, while we did not observe the formation of the desired products **3af** and 3**ag**. It should be noted that all the synthesized compounds were stable and fully characterized by NMR and HRMS.

**Scheme 3:**
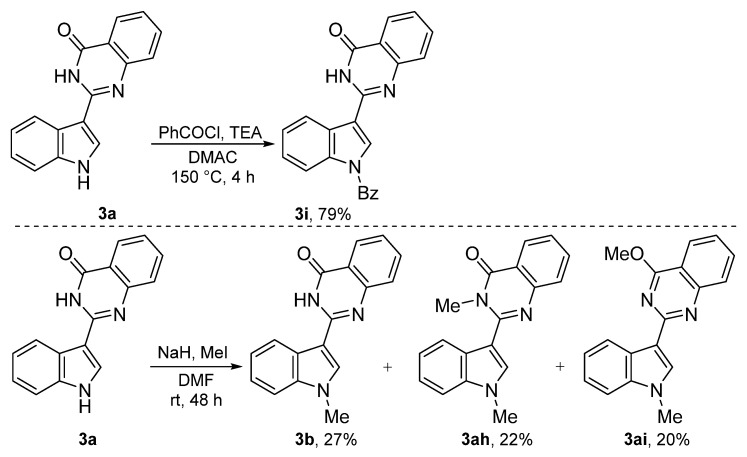
The functionalization of the *N*-atom of model indolylquinazolinone **3a**.

Finally, we studied some reactivity of the model 2-(1*H*-indol-3-yl)quinazolin-4(3*H*)-one (**3a**) (Scheme 3). Initially, we checked the possibility of the functionalization of the nitrogen atom and synthesized various *N*-substituted indolylquinazolinones **3**. The problem of the hydrolysis of the protecting group at the nitrogen atom can be solved by changing the order of the reaction steps. The treatment of indolylquinazolinone **3a** with benzoyl chloride afforded 2-(1-benzoyl-1*H*-indol-3-yl)quinazolin-4(3*H*)-one (**3i**) in a good yield. The sequential treatment of quinazolinone **3a** with NaH and methyl iodide afforded a mixture of three products in equal amounts. Along with the expected mono- **3b** and disubstituted indolylquinazolinones **3ah**, we observed the formation of 4-methoxy-2-(1-methyl-1*H*-indol-3-yl)quinazoline (**3ai**). (Scheme 3).

**Scheme 4:**
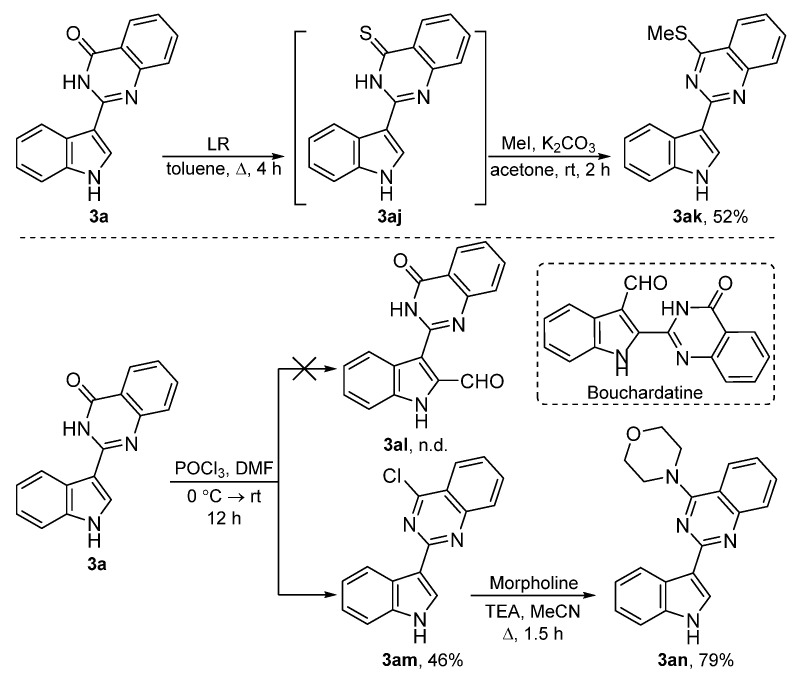
The functionalization of the amide moiety of model indolylquinazolinone **3a**.

According to the literature data, it is known that some thioquinazolinones are characterized by increased antimycobacterial activity compared to that of quinazolinones [48]. To study the reactivity of the substrate **3a**, we developed a method of thionation with Lawesson’s reagent (LR) and a subsequent alkylation affording the target 2-(1*H*-indol-3-yl)-4-(methylthio)quinazolinone (**3ak**) in a moderate yield over two steps (Scheme 4). An attempt to prepare an isomer of bouchardatine **3al** using the Vilsmeier–Haack formylation of the 2-(1*H*-indol-3-yl)quinazolin-4(3*H*)-one (**3a**) was unsuccessful (Scheme 4). Instead of the expected product, 4-chloro-2-(1*H*-indol-3-yl)quinazoline (**3am**) was isolated in a moderate, nonoptimized yield. The obtained 4-chloroquinazoline **3am** was treated with morpholine and the desired 4-[2-(1*H*-indol-3-yl)quinazolin-4-yl]morpholine (**3an**) was obtained in a high yield. Both observations demonstrated the possibility of accessing a wide range of 4-amino- and 4-thiosubstituted quinazolines.

### 2.2. In Vitro Biological Evaluation

#### 2.2.1. Antibacterial Activity

The 32 synthesized compounds were evaluated for their antimicrobial activity against *Staphylococcus aureus*, *Escherichia coli*, *Mycobacterium smegmatis,* and *Candida albicans*. High activity against staphylococci was demonstrated by indolylquinazolinone **3k** (Table 1). Compound **3k** inhibited the growth of *S. aureus* ATCC 25923, a standard reference strain for evaluating the activity of antimicrobial compounds, showing an MIC of 3.90 μg/mL. At the same time, its MIC against the reference strain *S. aureus* ATCC 43300 (MRSA) was even lower and did not exceed 1 μg/mL. This is a very promising result, as MRSA is now widespread and a significant problem in human and veterinary medicine, because it is resistant to many antibacterial compounds. Additionally, we studied the activity of compound **3k** against another staphylococcus species, *S. epidermidis* ATCC 12228. The MIC and MBC (minimum bactericidal concentration) in this case were 7.80 and 12.50 µg/mL, respectively. All of the compounds were found to be inactive against the Gram-negative bacterium *E. coli*. In addition to their antibacterial properties, the antimycotic activity of the synthesized substances against *Candida albicans* was studied (minimum fungicidal concentration—MFC). Compounds **3d**, **3p**, **3t**, **3u**, **5a**, and **6u** showed moderate activity against this microorganism, while substances **3k** and **3ai** showed more pronounced activity (MIC 7.80, 62.50 µg/mL, respectively) (Table 1). Other quinazolinone derivatives **3** were found to be inactive (See Supplementary Materials).

Mycobacteria are a special group of bacteria with unique structural and physiological features. This group includes both saprophytes and animal or human pathogens, in particular, the causative agent of tuberculosis, *Mycobacterium tuberculosis*. *Mycobacterium smegmatis* is widely used as a model microorganism for studying antimycobacterial activity. Most of the compounds were found to be inactive against *M. smegmatis*. Although *M. smegmatis* is used as a model organism for the study of *M. tuberculosis*, these bacteria are not identical and have their own individual characteristics. Particularly, there are significant differences in their growth rate and in the structure of their cell walls. Considering the high activity of compound **3k** against Gram-positive organisms (staphylococci), we studied its activity against *M. tuberculosis* H_37_Rv using a BASTEC MGIT 960 growth system [49,50]. This compound inhibited the growth of *M. tuberculosis* H_37_Rv within 41 days at a concentration of 10 μg/mL. In the presence of 5 μg/mL of this substance, the growth of the mycobacteria was not observed for 18 days, and in the presence of 1 μg/mL, for 5 days (Table 2). Therefore, indolylquinazolinone **3k** is the leading compound that is promising for further studies in the field of anti-tubercular activity.

It is worth noting that several compounds studied in this work have previously been tested for their antibacterial activity. Compounds **3k**, **3t**, and **3z** are active against several species of *Leishmania*, which are intracellular parasites [8,51]. Indolylquinazolinone **3k** has an antibacterial effect against *M. tuberculosis*, which is confirmed by our studies [7]. Compounds **3t**, **3u**, and **3x**–**z** have been tested against *E. coli* and *S. aureus*, but did not show high activity [52], which is in agreement with our results. Quinazolinone **5a** has an antibacterial effect against *E. coli*, *S. aureus*, *Bacillus subtilis*, and other bacteria [53,54], but this result is not confirmed by our studies. Perhaps this is due to the difference in the methodological approaches to the antimicrobial activity determination. In our experiments, only the antifungal activity of compound **5a** was noted, which had not previously been shown.

#### 2.2.2. Antibiofilm Activity

**Figure 2:**
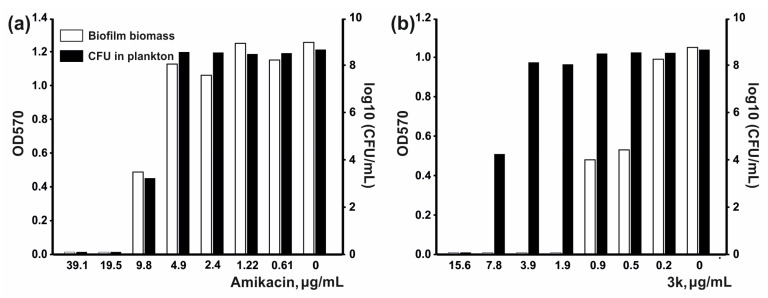
Influence of sublethal concentrations of amikacin (**a**) and 2-(5-iodo-1*H*-indol-3-yl)quinazolin-4(3*H*)-one (**3k**) (**b**) on the *S. aureus* ATCC 25923 biofilm biomass (OD570) and the number of colony-forming units (CFU) in plankton. Diagrams show the mean values of three (**a**) and four (**b**) experiments.

Biofilms are communities of microorganisms that are attached to a surface and play a significant role in bacterial persistence. This is a significant problem in the health industry and much research has been performed to reduce microbial colonization and prevent biofilm formation [55]. In this study, the ability of the most active synthesized compound **3k** to prevent biofilm formation was tested. Most of the known antibiotics inhibit biofilm formation by affecting cell viability (decrease in biofilm biomass correlates with a decrease in the number of cells in a culture). We evaluated the number of living cells in a culture during biofilm formation in the presence of various concentrations of 2-(5-iodo-1*H*-indol-3-yl)quinazolin-4(3*H*)-one (**3k**) and the control antibiotic amikacin to ensure that the observed inhibition of biofilm formation was not the result of a decrease in the number of planktonic cells. Compound **3k** was able to prevent the appearance of biofilms of staphylococci without affecting the number of cells in a culture (Figure 2b). In contrast, amikacin reduced the formation of a microbial biofilm due to its lethal effect on planktonic cells (Figure 2a).

#### 2.2.3. Cytotoxicity Activity

**Figure 3:**
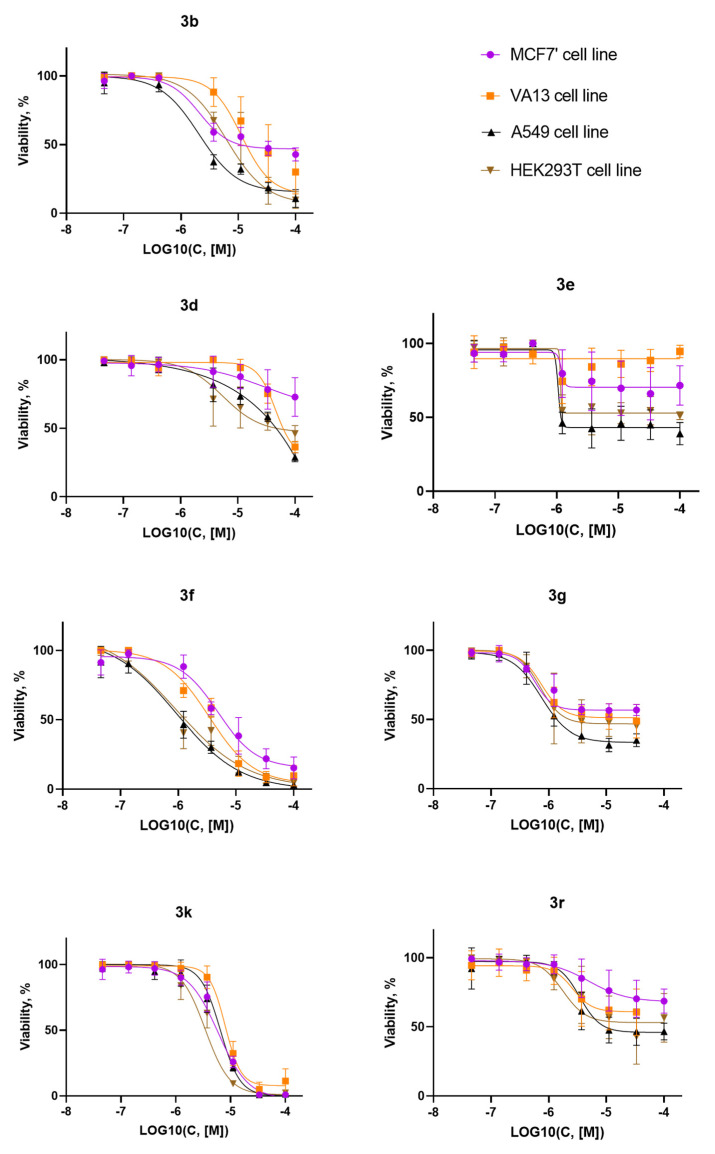
In vitro cytotoxicity of the compounds **3b**, **3d**–**3g**, **k**, and 3**r** to cells MCF7′, A549, HEK293T, and VA13 via MTT assay.

Compounds **3c**, **f**, **g**, **k**, **r**, and 3**z** showed the highest cytotoxicity, for which IC_50_ or IC_50abs_ were observed in the early micromolar range (less than 10 μM) against the tested cell lines. The dependences of the number of cells on the concentration of the compounds are shown in Figure 3. It should be noted that compounds **3b**, **3e**, and **3g** showed a preferential suppression of the growth of rapidly dividing A549 cells compared to slower growing fibroblasts of non-tumor etiology. In addition, there was a significant proportion of living cells compared to the untreated control over a wide range of high concentrations. The greatest difference was observed for compound **3e**. Although this dependence can be explained, for example, by the solubility of the compounds, it is more likely to be explained by a cytostatic rather than a cytotoxic effect. A similar pattern of cytotoxicity was observed, for example, for antitubulin compounds in the nanomolar concentration range.

For another group of compounds (**3f** and **3k**, etc.), there was a different pattern of cytotoxicity, when complete cell death was observed at high concentrations. Therefore, two parameters of action on the cells were calculated for the compounds: IC_50_ (half of the maximal inhibition) and IC_50abs_ (half of the absolute quantity of cells) (see Table 3). The rest of the compounds had a low cytotoxicity or did not show noticeable toxic effects in the studied concentration range.

### 2.3. In Silico Characterization of Substituted Indoles Binding to Long RSH Proteins

#### Molecular Docking of Substituted Indoles

Searching for new chemical compounds that are targeted to non-essential bacterial adaptation pathways, such as stringent response, is an attractive approach for developing antimicrobials preventing bacterial persistence, biofilm formation, quorum sensing, and virulence [56]. Previously, researchers have identified indole derivatives as a chemotype capable of binding the Rel_Seq_ (p)ppGpp synthetase/hydrolase synthetic domain via virtual screening and thermal shift assays [26]. Based on these data, we selected a set of substituted indoles from our library of compounds to assess the possibility of their binding to Rel_Mtb_ or Rel_Seq_ proteins in synthetic domain (SYN) active sites by using molecular docking methods. The Rel_Mtb_ crystal structure unbound to the substrate [57] was used to analyze the ligand binding to an inactive state synthetase, while the Rel_Seq_ structure complexed with a GDP (guanosine diphosphate) substrate [58] was used to investigate the binding to (p)ppGpp synthetase in a catalytically competent state. The reference ligand indole-5-carboxylic acid demonstrated a Rel_Seq_ active site binding energy of −6.42 kcal/mol (Table 4), consistent with the result of −6.41 kcal/mol calculated in the previous research (in which ligand mentioned as I2) [26].

**Figure 4:**
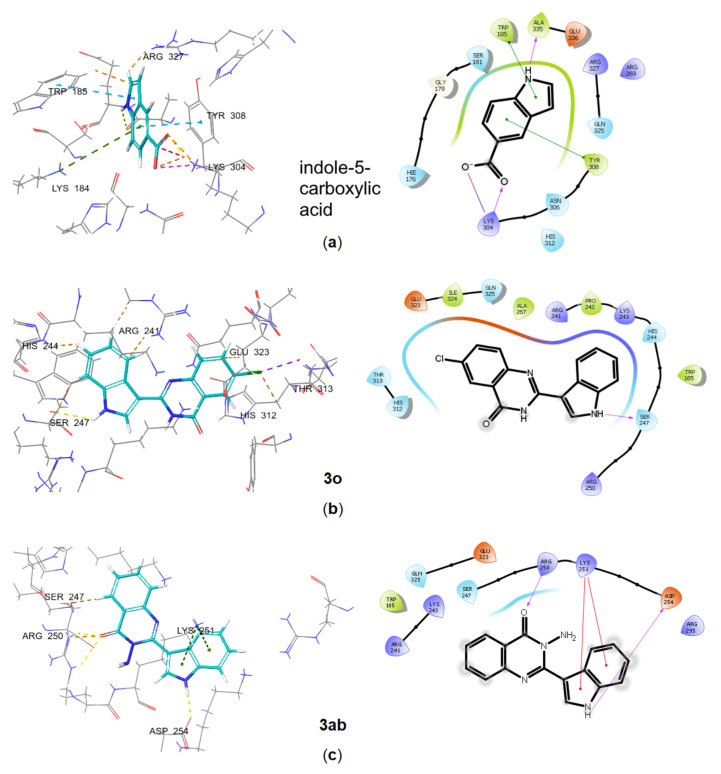
Best energy interactions between ligands and Rel_Seq_ protein synthetic domain active site. (**a**) Reference ligand indole-5-carboxylic acid; (**b**) **3o** compound; and (**c**) **3ab** compound. Protein chains are shown in grey thin sticks, ligands are shown in blue thick sticks, interactions are shown in dashed lines: yellow hydrogen bonds, blue pi–pi stacking and green pi-cation interactions, purple salt bridges.

The docked ligands interacted with the Arg241 and His312 residues (Figure 4b,c), which are involved in GDP substrate binding [58]. Indolylquinazolinones and their derivatives demonstrated weaker binding in the Rel_Seq_ active site than the reference ligand, with the most negative docking score of −4.76 kcal/mol (Table 4). This might have been due to the planar structure of the analyzed compounds, lacking the flexibility to optimally fit the tight binding pocket in the substrate-bound state (Figure 4b,c). However, eight analyzed compounds demonstrated stronger binding energies (from −7.61 to −6.56 kcal/mol) compared to the reference ligand (−6.55 kcal/mol) in case of the substrate-unbound Rel_Mtb_ structure SYN active site (Table 4).

**Figure 5:**
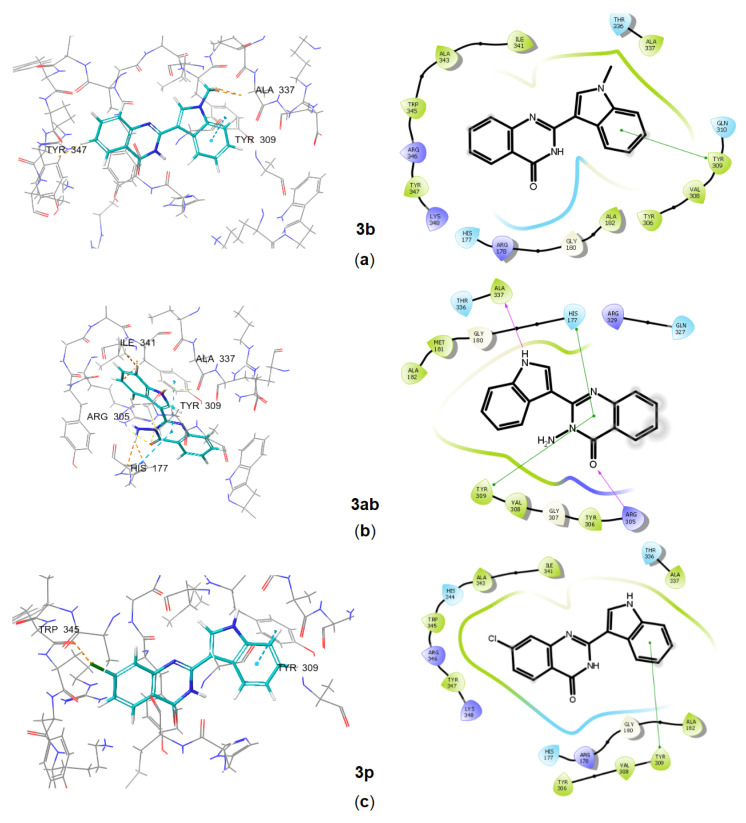
Best energy interactions between ligands and Rel_Mtb_ protein synthetic domain active site. (**a**) **3b** compound, (**b**) **3ab** compound, and (**c**) **3p** compound. Color coding is identical to Figure 4.

Moreover, the indole derivatives interacted with the Tyr309 residue (Figure 5a–c), which has been shown to be essential for Rel_Mtb_ (p)ppGpp synthesis [59]. Indole derivatives are therefore promising candidates for developing inhibitors of (p)ppGpp synthetases.

## 3. Materials and Methods

### 3.1. Instrumentation

^1^H and ^13^C NMR spectra were recorded on a «Bruker Avance III HD 400» (400 MHz for ^1^H and 100 MHz for ^13^C NMR) at 40 °C. The chemical shifts (δ) were measured in ppm with respect to the solvent ([D_6_] DMSO, ^1^H: δ = 2.50 ppm, ^13^C: δ = 39.52 ppm). The coupling constants (*J*) are given in Hertz (Hz). The splitting patterns of an apparent multiplets associated with the averaged coupling constants were designated as s (singlet), d (doublet), t (triplet), q (quartet), m (multiplet), dd (doublet of doublets), ddd (doublet of doublet of doublets), and br. (broadened). High-resolution mass measurements (HRMS) were carried out using a BrukermicroTOF-QTM ESI-TOF mass spectrometer. A GC/MS analysis was performed on an «Agilent 7890B» interfaced to an «Agilent 5977A» mass selective detector. The melting points were determined with a «Stuart SMP 30». Data sets for X-Ray diffraction were collected with a «New Xcalibur, Ruby» diffractometer. Column chromatography was performed on silica gel Macherey Nagel (40–63 μm). Flash column chromatography was performed over silica gel (0.04–0.063 mm), using a mixture of EtOAc, petroleum ether. TLC plates were visualized by exposure to ultraviolet light. All the reactions were carried out using freshly distilled and dry solvents from solvent stills.

### 3.2. Materials

Starting 1*H*-indole-3-carboxaldehyde (purity 97%), furfural (99%), 5-methylfurfural (99%), 2-thiophenecarboxaldehyde (98%), 4-pyridinecarboxaldehyde (97%), 2-naphthaldehyde (98%), cyclohexanecarbaldehyde (97%), Lawesson’s reagent (97%), morpholine (99%), benzoyl chloride (99%), iodomethane (99%), and 2-aminobenzenesulfonamide (98%) were purchased from Sigma-Aldrich (St. Louis, MO, USA). The reagents for the analysis of the antimicrobial activity: Cefazolin—MP Biomedicals (Illkirch, France), Cefotaxime—PROMED (Saransk, Russia), Chloramphenicol—AppliChem (Darmstadt, Germany), Amikacin sulfate—Sigma-Aldrich (St. Louis, MO, USA), Fluconazole—Sigma-Aldrich (St. Louis, MO, USA), Amphotericin B—Sigma-Aldrich (St. Louis, MO, USA), Isoniazid—Sigma (St. Louis, MO, USA), LB-broth—VWR (Radnor, PA, USA), and LB-agar—Sigma (St. Louis, MO, USA).

### 3.3. Synthesis

#### 3.3.1. Synthesis of the Starting Substrates

Starting 1-methylindole-3-carboxaldehyde (**1b**), 1-*n*-butyl-1*H*-indole-3-carbaldehyde (**1e**) [60], 1-ethyl-1*H*-indole-3-carbaldehyde (**1c**), 1-isopropyl-1*H*-indole-3-carbaldehyde (**1d**) [61], 1-benzyl-1*H*-indole-3-carbaldehyde (**1f**) [62], 1-phenyl-1*H*-indole-3-carbaldehyde (**1g**) [63], 1-acetyl-1*H*-indole-3-carbaldehyde (**1h**) [64], 1-benzoyl-1*H*-indole-3-carbaldehyde (**1i**) [65], 1-tosyl-1*H*-indole-3-carbaldehyde (**1j**) [66], 5-iodo-1*H*-indole-3-carbaldehyde (**1k**) [67], 5-bromo-1*H*-indole-3-carbaldehyde (**1l**) [68], 2-phenyl-1*H*-indole-3-carbaldehyde (**1m**) [69], 2-(4-nitrophenyl)-1*H*-indole-3-carbaldehyde (**1n**) [70], benzofuran-2-carbaldehyde (**1p**) [71], 5-ethylfuran-2-carbaldehyde (**1s**) [72], 1-methyl-1*H*-pyrrole-2-carbaldehyde (**1t**) [73], anthranilamide (**2a**) [74], 2-amino-4-chlorobenzamide (**2b**) [75], 2-aminobenzohydrazide (**2e**) [76], 2-hydroxybenzamide (**2f**) [77], 2-amino-5,6-dihydro-4*H*-cyclopentathiophene-3-carboxamide (**2g**) [78], and 2-amino-2-phenylacetamide (**2i**) [79] were synthesized according to known procedures.

#### 3.3.2. Synthesis of 2-(1*H*-indol-3-yl)quinazolin-4(3*H*)-one (**3a**) [80]

To a solution of 1*H*-indole-3-carboxaldehyde (**1a**) (1.3 mmol, 0.189 g) and anthranilamide (**2a**) (1.3 mmol, 0.177 g) in dry acetonitrile (2.5 mL), *p*-TSA (0.5 mmol, 0.095 g) was added. The reaction mixture was refluxed for 4 h (TLC control). Then, the reaction mixture was poured into H_2_O (50 mL). The formed precipitate was filtered. The products were purified using column chromatography on silica gel using the mixture of petroleum ether/EtOAc (1:1) as an eluent and recrystallized from ethanol. There was a yield of 0.112 g (33%) and it was a colorless oil. ^1^H NMR (400 MHz, DMSO-*d*_6_) δ = 12.08 (br.s, 1H), 11.81 (br.s, 1H), 8.72—8.70 (m, 1H), 8.55 (d, *J* = 2.8 Hz, 1H), 8.13–8.11 (m, 1H), 7.81–7.72 (m, 2H), 7.50–7.48 (m, 1H), 7.43–7.39 (m, 1H), and 7.27–7.20 (m, 2H) ppm; ^13^C{^1^H} NMR (100 MHz, DMSO-*d*_6_) δ = 162.0, 150.2, 149.7, 136.8, 134.2, 129.0, 126.9, 125.7, 125.5, 125.0, 122.5, 122.3, 120.8, 120.4, 111.9, and 108.6 ppm.

Indole (**4a**) and quinazolin-4(3*H*)-one (**5a**) were isolated as by-products in the synthesis of 2-(1*H*-indol-3-yl)quinazolin-4(3*H*)-one (**3a**). The spectral data of the indole (**4a**) were identical to the commercially available ones. Quinazolin-4(3H)-one (**5a**) [81]. There was a yield of 0.023 g (12%) and it was a white solid. Mp. = 215–216 °C (EtOH, lit. [81] 213–214 °C). ^1^H NMR (400 MHz, DMSO-*d*_6_) δ = 12.16 (br.s, 1H), 8.12 (br.d, *J* = 8.0 Hz, 1H), 8.08 (s, 1H), 7.82–7.78 (m, 1H), 7.66 (d, *J* = 8.0 Hz, 1H), and 7.53–7.49 (m, 1H) ppm; ^13^C{^1^H} NMR (100 MHz, DMSO-*d*_6_) δ = 160.6, 148.7, 145.2, 134.2, 127.1, 126.6, 125.7, and 122.6 ppm.

#### 3.3.3. Synthesis of 2-(1*H*-indol-3-yl)-2,3-dihydroquinazolin-4(1*H*)-one (**6a**) [82]

To a suspension of 1*H*-indole-3-carboxaldehyde (**1a**) (1.3 mmol, 0.189 g) and Al_2_O_3_ (acidic) (2.3 mmol, 0.235 g) in dry acetonitrile (2.5 mL), anthranilamide (**2a**) (1.3 mmol, 0.177 g) was added. The reaction mixture was refluxed for 55 h (TLC control). Then, the Al_2_O_3_ was filtered off and washed with hot EtOAc (3 × 5 mL). The combined organic fractions were concentrated to dryness under reduced pressure. The product was purified using column chromatography on silica gel using the mixture of petroleum ether/EtOAc (1:1) as an eluent. There was a yield of 0.154 g (45%) and it was a colorless oil. ^1^H NMR (400 MHz, DMSO-*d*_6_) δ = 11.09 (br.s, 1H), 8.08 (br.s, 1H), 7.80–7.78 (m, 1H), 7.70–7.68 (m, 1H), 7.43–7.41 (m, 2H), 7.27–7.23 (m, 1H), 7.15–7.11 (m, 1H), 7.04–7.01 (m, 1H), 6.92 (br.s, 1H), 6.79–6.77 (m, 1H), 6.73–6.69 (m, 1H), and 6.07 (s, 1H) ppm; ^13^C{^1^H} NMR (100 MHz, DMSO-*d*_6_) δ = 164.2, 148.8, 136.6, 133.0, 127.5, 125.4, 124.6, 121.4, 120.0, 118.7, 117.0, 115.3, 114.43, 114.38, 111.6, and 61.7 ppm.

Indole (**4a**) was isolated as by-product in the synthesis of 2-(1*H*-indol-3-yl)-2,3-dihydroquinazolin-4(1*H*)-one (**6a**). The spectral data of the indole (**4a**) were identical to the commercially available ones.

#### 3.3.4. General Procedure for the Synthesis of Quinazolin-4(3*H*)-Ones **3a**–**3g**, **3k**–**3ab**

To a suspension of aldehyde **1** (1.3 mmol) and Na_2_S_2_O_5_ (4.5 mmol, 0.855 g) in *N*,*N*-dimethylacetamide (2.5 mL), amide **2** (1.3 mmol) and H_2_O (4.5 mmol, 81 μL) were added. The reaction mixture was heated at 150 °C for 6.5–55 h (TLC control). Then, the reaction mixture was poured into H_2_O (50 mL). The formed precipitate was filtered. The products **3a**,**d**,**f**,**g**,**k**,**l**,**o**–**r**,**t**,**w**,**x**,**ab** were purified using column chromatography on silica gel using the mixture of petroleum ether/EtOAc (1:1) as an eluent and recrystallized from a suitable solvent. The products **3b**,**c**,**e**,**s**,**u**,**v**,**y**,**z**,**aa** were recrystallized from a suitable solvent without prior purification using column chromatography.

2-(1H-Indol-3-yl)quinazolin-4(3H)-one (**3a**) [80]. A yield of 0.326 g (96%), 14 h, white solid. Mp. ≥ 320 °C, decomposition (EtOH, lit. [80] > 300 °C). All the spectral data of 2-(1*H*-indol-3-yl)quinazolin-4(3*H*)-one (**3a**) were consistent with those described above.

2-(1-Methyl-1H-indol-3-yl)quinazolin-4(3H)-one (**3b**) [83]. A yield of 0.211 g (59%), 43 h, white solid. Mp. ≥ 324 °C, decomposition (EtOH, lit. [83] 290–295 °C). ^1^H NMR (400 MHz, DMSO-*d*_6_) δ = 12.07 (s, 1H), 8.72–8.70 (m, 1H), 8.51 (s, 1H), 8.12–8.10 (m, 1H), 7.80–7.71 (m, 2H), 7.55 (br.d, *J* = 7.6 Hz, 1H), 7.43–7.39 (m, 1H), 7.33–7.25 (m, 2H), and 3.88 (s, 3H) ppm; ^13^C{^1^H} NMR (100 MHz, DMSO-*d*_6_) δ = 162.0, 149.9, 149.6, 137.3, 134.2, 132.8, 126.8, 125.9, 125.7, 125.0, 122.6, 122.4, 121.1, 120.4, 110.3, 107.6, and 33.2 ppm.

2-(1-Ethyl-1H-indol-3-yl)quinazolin-4(3H)-one (**3c**). A yield of 0.327 g (87%), 47 h, pale beige solid. Mp. = 283–284 °C (EtOH). ^1^H NMR (400 MHz, DMSO-*d*_6_) δ = 12.05 (br.s, 1H), 8.72 (d, *J* = 7.5 Hz, 1H), 8.62 (br.s, 1H), 8.12 (d, *J* = 7.6 Hz, 1H), 7.80–7.72 (m, 2H), 7.58 (d, *J* = 7.6 Hz, 1H), 7.43–7.39 (m, 1H), 7.31–7.24 (m, 2H), 4.27 (q, *J* = 7.2 Hz, 2H), and 1.48 (t, *J* = 7.2 Hz, 3H) ppm; ^13^C{^1^H} NMR (100 MHz, DMSO-*d*_6_) δ = 161.9, 149.9, 149.7, 136.5, 134.2, 131.1, 126.9, 126.0, 125.7, 125.0, 122.51, 122.50, 121.1, 120.4, 110.3, 107.7, 40.8, and 14.7 ppm. HRMS (ESI) Calcd for C_18_H_16_N_3_O [M + H]^+^ 290.1288; Found 290.1278.

2-(1-Isopropyl-1H-indol-3-yl)quinazolin-4(3H)-one (**3d**). A yield of 0.319 g (81%), 24 h, beige solid. Mp. = 275–276 °C (EtOH). ^1^H NMR (400 MHz, DMSO-*d*_6_) δ = 12.03 (br.s, 1H), 8.78 (br.s, 1H), 8.73–8.71 (m, 1H), 8.10 (br.d, *J* = 7.6 Hz, 1H), 7.80–7.72 (m, 2H), 7.62 (br.d, *J* = 7.6 Hz, 1H), 7.43–7.39 (m, 1H), 7.31–7.24 (m, 2H), 4.89–4.80 (m, 1H), 1.54 (s, 3H), and 1.52 (s, 3H) ppm; ^13^C{^1^H} NMR (100 MHz, DMSO-*d*_6_) δ = 161.9, 150.0, 149.7, 136.3, 134.2, 128.5, 126.9, 126.0, 125.7, 125.0, 122.5, 122.4, 121.1, 120.3, 110.4, 107.8, 47.1, and 22.3 (2C) ppm. HRMS (ESI) Calcd for C_19_H_18_N_3_O [M + H]^+^ 304.1444; Found 304.1452.

2-(1-Butyl-1H-indol-3-yl)quinazolin-4(3H)-one (**3e**). A yield of 0.317 g (77%), 55 h, white solid. Mp. = 264–265 °C (EtOH). ^1^H NMR (400 MHz, DMSO-*d*_6_) δ = 12.05 (br.s, 1H), 8.72 (br.d, *J* = 8.0 Hz, 1H), 8.59 (s, 1H), 8.12–8.10 (m, 1H), 7.80–7.71 (m, 2H), 7.60 (d, *J* = 8.0 Hz, 1H), 7.43–7.39 (m, 1H), 7.31–7.24 (m, 2H), 4.25 (t, *J* = 7.2 Hz, 2H), 1.88–1.81 (m, 2H), 1.36–1.31 (m, 2H), and 0.94 (t, *J* = 7.2 Hz, 3H) ppm; ^13^C{^1^H} NMR (100 MHz, DMSO-*d*_6_) δ = 161.9, 149.9, 149.6, 136.8, 134.2, 131.8, 126.8, 125.9, 125.6, 125.0, 122.51, 122.48, 121.0, 120.3, 110.4, 107.6, 45.6, 31.2, 19.3, and 13.4 ppm. HRMS (ESI) Calcd for C_20_H_20_N_3_O [M + H]^+^ 318.1601; Found 318.1598.

2-(1-Benzyl-1H-indol-3-yl)quinazolin-4(3H)-one (**3f**). A yield of 0.411 g (90%), 19 h, white solid. Mp. = 307–308 °C (EtOH). ^1^H NMR (400 MHz, DMSO-*d*_6_) δ = 12.12 (br.s, 1H), 8.73–8.71 (m, 1H), 8.66 (br.s, 1H), 8.11 (d, *J* = 7.6 Hz, 1H), 7.80–7.72 (m, 2H), 7.63–7.60 (m, 1H), 7.43–7.40 (m, 1H), 7.36–7.33 (m, 4H), 7.31–7.24 (m, 3H), and 5.50 (s, 2H) ppm; ^13^C{^1^H} NMR (100 MHz, DMSO-*d*_6_) δ = 161.9, 149.8, 149.6, 137.0, 136.7, 134.2, 132.2, 128.6 (2C), 127.6, 127.4 (2C), 126.9, 126.1, 125.7, 125.1, 122.7, 122.5, 121.2, 120.4, 110.7, 108.3, and 49.7 ppm. HRMS (ESI) Calcd for C_23_H_18_N_3_O [M + H]^+^ 352.1444; Found 352.1451.

2-(1-Phenyl-1H-indol-3-yl)quinazolin-4(3H)-one (**3g**). A yield of 0.377 g (86%), 14 h, pale yellow solid. Mp. = 344–345 °C (EtOH). ^1^H NMR (400 MHz, DMSO-*d*_6_) δ = 12.14 (br.s, 1H), 8.86 (s, 1H), 8.84–8.82 (m, 1H), 8.14 (br.d, *J* = 8.0 Hz, 1H), 7.84–7.78 (m, 2H), 7.71–7.67 (m, 4H), 7.65–7.61 (m, 1H), 7.53–7.50 (m, 1H), 7.48–7.45 (m, 1H), and 7.37–7.35 (m, 2H) ppm; ^13^C{^1^H} NMR (100 MHz, DMSO-*d*_6_) δ = 161.9, 138.9, 138.1, 136.1, 134.3, 131.7, 130.0 (2C), 127.4, 127.1, 126.6, 125.7, 125.5, 124.0 (2C), 123.7, 122.9, 122.0, 120.6, 110.8, 110.6, and 110.1 ppm. HRMS (ESI) Calcd for C_22_H_16_N_3_O [M + H]^+^ 338.1288; Found 338.1288.

2-(5-Iodo-1H-indol-3-yl)quinazolin-4(3H)-one (**3k**) [8]. A yield of 0.478 g (95%), 14.5 h, beige solid. Mp. ≥ 308 °C, decomposition (EtOH, lit. [8] 309–311 °C). ^1^H NMR (400 MHz, DMSO-*d*_6_) δ = 12.13 (br.s, 1H), 11.98 (br.s, 1H), 9.06 (d, *J* = 1.6 Hz, 1H), 8.53 (s, 1H), 8.12 (dd, *J* = 8.0, 0.8 Hz, 1H), 7.81–7.78 (m, 1H), 7.73–7.70 (m, 1H), 7.51 (dd, *J* = 8.4, 1.6 Hz, 1H), 7.45–7.41 (m, 1H), and 7.36 (d, *J* = 8.4 Hz, 1H) ppm; ^13^C{^1^H} NMR (100 MHz, DMSO-*d*_6_) δ = 161.9, 149.8, 149.4, 135.9, 134.3, 130.63, 130.56, 129.8, 127.9, 126.9, 125.7, 125.2, 120.4, 114.4, 107.8, and 85.1 ppm.

2-(5-Bromo-1H-indol-3-yl)quinazolin-4(3H)-one (**3l**). A yield of 0.331 g (75%), 14 h, beige solid. Mp. ≥ 340 °C, decomposition (EtOH). ^1^H NMR (400 MHz, DMSO-*d*_6_) δ = 12.14 (br.s, 1H), 12.01 (br.s, 1H), 8.85 (d, *J* = 1.6 Hz, 1H), 8.58 (s, 1H), 8.12 (dd, *J* = 8.0, 0.8 Hz, 1H), 7.81–7.72 (m, 2H), 7.47 (d, *J* = 8.4 Hz, 1H), 7.45–7.41 (m, 1H), and 7.37 (dd, *J* = 8.8, 2.0 Hz, 1H) ppm; ^13^C{^1^H} NMR (100 MHz, DMSO-*d*_6_) δ = 161.9, 149.8, 149.4, 135.5, 134.3, 130.2, 127.2, 126.9, 125.7, 125.2, 125.1, 124.4, 120.4, 114.0, 113.6, and 108.2 ppm. HRMS (ESI) Calcd for C_16_H_11_BrN_3_O [M + H]^+^ 340.0080; Found 340.0080.

2-(2-Phenyl-1H-indol-3-yl)quinazolin-4(3H)-one *(***3m**) was determined using GC/MS in trace amounts. Quinazolin-4(3*H*)-one (**5a**) was isolated as by-product in the synthesis of 2-(2-phenyl-1*H*-indol-3-yl)quinazolin-4(3*H*)-one (**3m**) in a 37% yield. All the spectral data of quinazolin-4(3*H*)-one (**5a**) were consistent with those described above.

2-[2-(4-Nitrophenyl)-1H-indol-3-yl]quinazolin-4(3H)-one *(***3n**) was determined using GC/MS in trace amounts. Quinazolin-4(3*H*)-one (**5a**) was isolated as by-product in the synthesis of 2-[2-(4-nitrophenyl)-1*H*-indol-3-yl]quinazolin-4(3*H*)-one (**3n**) in a 59% yield. All the spectral data of quinazolin-4(3*H*)-one (**5a**) were consistent with those described above.

6-Chloro-2-(1H-indol-3-yl)quinazolin-4(3H)-one (**3o**). A yield of 0.350 g (91%), 20 h, pale beige solid. Mp. ≥ 390 °C, decomposition (EtOH). ^1^H NMR (400 MHz, DMSO-*d*_6_) δ = 12.24 (br.s, 1H), 11.85 (br.s, 1H), 8.68 (br.d, *J* = 6.8 Hz, 1H), 8.55 (d, *J* = 2.0 Hz, 1H), 8.04 (s, 1H), 7.79–7.73 (m, 2H), 7.49 (br.d, *J* = 6.8 Hz, 1H), and 7.26–7.20 (m, 2H) ppm; ^13^C{^1^H} NMR (100 MHz, DMSO-*d*_6_) δ = 161.1, 150.7, 148.4, 136.8, 134.3, 129.4, 129.1, 129.0, 125.4, 124.7, 122.6, 122.3, 121.5, 120.9, 111.9, and 108.3 ppm. HRMS (ESI) Calcd for C_16_H_11_ClN_3_O [M + H]^+^ 296.0585; Found 296.0582.

7-Chloro-2-(1H-indol-3-yl)quinazolin-4(3H)-one (**3p**). A yield of 0.357 g (93%), 23 h, pale beige solid. Mp. = 323–324 °C (EtOH). ^1^H NMR (400 MHz, DMSO-*d*_6_) δ = 12.19 (br.s, 1H), 11.87 (br.s, 1H), 8.70 (d, *J* = 7.2 Hz, 1H), 8.57 (d, *J* = 2.4 Hz, 1H), 8.09 (d, *J* = 8.4 Hz, 1H), 7.78 (s, 1H), 7.50–7.48 (m, 1H), 7.42–7.40 (m, 1H), and 7.26–7.20 (m, 2H) ppm; ^13^C{^1^H} NMR (100 MHz, DMSO-*d*_6_) δ = 161.4, 151.6, 150.9, 138.9, 136.8, 129.6, 127.7, 125.9, 125.5, 125.1, 122.6, 122.4, 121.0, 119.1, 111.9, and 108.3 ppm. HRMS (ESI) Calcd for C_16_H_11_ClN_3_O [M + H]^+^ 296.0585; Found 296.0582.

2-(1H-Indol-3-yl)-6-iodoquinazolin-4(3H)-one (**3q**). A yield of 0.226 g (45%), 32 h, pale brown solid. Mp. ≥ 308 °C, decomposition (1,4-dioxane/EtOH). ^1^H NMR (400 MHz, DMSO-*d*_6_) δ = 12.21 (br.s, 1H), 11.85 (br.s, 1H), 8.68–8.66 (m, 1H), 8.54 (br.s, 1H), 8.37 (br.s, 1H), 8.05–8.03 (m, 1H), 7.54–7.48 (m, 2H), and 7.25–7.20 (m, 2H) ppm; ^13^C{^1^H} NMR (100 MHz, DMSO-*d*_6_) δ = 160.7, 150.8, 149.0, 142.5, 136.8, 134.0, 129.4, 129.1, 125.4, 122.6, 122.3, 122.2, 120.9, 111.9, 108.4, and 89.1 ppm. HRMS (ESI) Calcd for C_16_H_11_IN_3_O [M + H]^+^ 387.9941; Found 387.9947.

N,N-dimethyl-1H-indole-3-carbothioamide (**7a**) was isolated as a by-product in the synthesis of 2-(1*H*-indol-3-yl)-6-iodoquinazolin-4(3*H*)-one (**3q**), 3-(1*H*-indol-3-yl)-2*H*-benzo[*e*][1,2,4]thiadiazine-1,1-dioxide (**3af**), and 2-(1*H*-indol-3-yl)-5-phenyl-3,5-dihydro-4*H*-imidazol-4-one (**3ag**) in 21%, 47%, and 50% yields, respectively. Yields of 0.056 g (21%) for **3q**, 0.125 g (47%) for **3af**, and 0.133 g (50%) for **3ag** were obtained and they were pale beige solids. Mp. ≥ 180 °C, decomposition (petroleum ether/EtOAc). ^1^H NMR (400 MHz, DMSO-*d*_6_) δ = 11.51 (br.s, 1H), 7.68–7.62 (m, 2H), 7.43 (s, 1H), 7.12 (br.s, 2H), and 3.42 (br.s, 6H) ppm; ^13^C{^1^H} NMR (100 MHz, DMSO-*d*_6_) δ = 193.2, 135.5, 127.0, 124.8, 121.6, 120.3, 119.9, 118.3, 111.8, and 43.3 (br.s, 2C) ppm. HRMS (ESI) Calcd for C_11_H_13_N_2_S [M + H]^+^ 205.0794; Found 205.0792. (CCDC 2269768 contains the supplementary crystallographic data for this paper. These data can be obtained free of charge from the Cambridge Crystallographic Data Centre via. Available online: www.ccdc.cam.ac.uk/structures; accessed on 14 June 2023; for details, see Supplementary Materials).

2-(1H-Indol-2-yl)quinazolin-4(3H)-one (**3r**) [84]. A yield of 0.271 g (80%), 15 h, grey solid. Mp. ≥ 326 °C, decomposition (EtOH, lit. [84] 302–304 °C). ^1^H NMR (400 MHz, DMSO-*d*_6_) δ = 12.54 (br.s, 1H), 11.73 (br.s, 1H), 8.16 (br.d, *J* = 7.6 Hz, 1H), 7.86–7.83 (m, 1H), 7.75–7.73 (m, 1H), 7.67–7.64 (m, 2H), 7.56–7.49 (m, 2H), 7.25–7.22 (m, 1H), and 7.09–7.05 (m, 1H) ppm; ^13^C{^1^H} NMR (100 MHz, DMSO-*d*_6_) δ = 161.6, 148.7, 146.5, 137.6, 134.5, 130.0, 127.4, 126.8, 126.1, 126.0, 123.9, 121.4, 121.1, 119.9, 112.3, and 104.9 ppm.

2-(Benzofuran-2-yl)quinazolin-4(3H)-one (**3s**) [85]. A yield of 0.235 g (69%), 6,5 h, beige solid. Mp. = 253–254 °C (EtOH) (lit. [85] 254–257 °C). ^1^H NMR (400 MHz, DMSO-*d*_6_) δ = 12.69 (br.s, 1H), 8.17 (br.d, *J* = 7.6 Hz, 1H), 8.06 (s, 1H), 7.85–7.73 (m, 4H), 7.57–7.47 (m, 2H), and 7.37–7.34 (m, 1H) ppm; ^13^C{^1^H} NMR (100 MHz, DMSO-*d*_6_) δ = 161.4, 154.8, 148.3, 147.6, 144.2, 134.6, 127.4, 127.3, 126.93, 126.91, 125.9, 123.7, 122.5, 121.5, 111.7, and 110.1 ppm.

2-(Furan-2-yl)quinazolin-4(3H)-one (**3t**) [84]. A yield of 0.234 g (85%), 9 h, beige solid. Mp. = 221–222 °C (EtOH) (lit. [84] 210–212 °C). ^1^H NMR (400 MHz, DMSO-*d*_6_) δ = 12.42 (br.s, 1H), 8.12 (br.d, *J* = 7.2 Hz, 1H), 7.98 (br.s, 1H), 7.82–7.78 (m, 1H), 7.69–7.67 (m, 1H), 7.62 (d, *J* = 3.2 Hz, 1H), 7.50–7.47 (m, 1H), and 6.74–6.73 (m, 1H) ppm; ^13^C{^1^H} NMR (100 MHz, DMSO-*d*_6_) δ = 161.4, 148.6, 146.4, 146.1, 143.9, 134.5, 127.1, 126.3, 125.8, 121.1, 114.3, and 112.4 ppm.

2-(5-Methylfuran-2-yl)quinazolin-4(3H)-one (**3u**) [86]. A yield of 0.209 g (71%), 9 h, brown solid. Mp. = 195–196 °C (EtOH) (lit. [86] 191–192 °C). ^1^H NMR (400 MHz, DMSO-*d*_6_) δ = 12.28 (br.s, 1H), 8.11 (d, *J* = 7.6 Hz, 1H), 7.81–7.77 (m, 1H), 7.69–7.67 (m, 1H), 7.53 (d, *J* = 3.0 Hz, 1H), 7.48–7.44 (m, 1H), 6.36 (d, *J* = 3.0 Hz, 1H), and 2.41 (s, 3H) ppm; ^13^C{^1^H} NMR (100 MHz, DMSO-*d*_6_) δ = 161.4, 156.0, 148.8, 144.4, 143.9, 134.4, 127.0, 126.0, 125.8, 120.9, 115.7, 108.8, and 13.4 ppm.

2-(5-Ethylfuran-2-yl)quinazolin-4(3H)-one (**3v**). A yield of 0.265 g (85%), 9 h, pale yellow solid. Mp. = 187–188 °C (EtOH). ^1^H NMR (400 MHz, DMSO-*d*_6_) δ = 12.30 (br.s, 1H), 8.11 (d, *J* = 8.0 Hz, 1H), 7.80–7.77 (m, 1H), 7.69–7.67 (m, 1H), 7.52 (d, *J* = 3.0 Hz, 1H), 7.48–7.44 (m, 1H), 6.37 (d, *J* = 3.0 Hz, 1H), 2.75 (q, *J* = 7.6 Hz, 2H), and 1.25 (t, *J* = 7.6 Hz, 3H) ppm; ^13^C{^1^H} NMR (100 MHz, DMSO-*d*_6_) δ = 161.5, 161.3, 148.8, 144.3, 144.0, 134.4, 127.0, 126.0, 125.8, 120.9, 115.5, 107.4, 20.9, and 11.7 ppm. HRMS (ESI) Calcd for C_14_H_13_N_2_O_2_ [M + H]^+^ 241.0972; Found 241.0971.

2-(1-Methyl-1H-pyrrol-2-yl)quinazolin-4(3H)-one (**3w**). A yield of 0.219 g (75%), 10 h, white solid. Mp. = 224 °C (EtOH). ^1^H NMR (400 MHz, DMSO-*d*_6_) δ = 11.98 (br.s, 1H), 8.09 (d, *J* = 7.6 Hz, 1H), 7.78–7.74 (m, 1H), 7.63–7.61 (m, 1H), 7.44–7.40 (m, 1H), 7.22 (d, *J* = 2.0 Hz, 1H), 7.08 (s, 1H), 6.15 (br.s, 1H), and 4.07 (s, 3H) ppm; ^13^C{^1^H} NMR (100 MHz, DMSO-*d*_6_) δ = 161.8, 148.7, 146.7, 134.2, 129.9, 126.9, 125.6, 125.5, 123.9, 120.3, 114.7, 107.6, and 37.4 ppm. HRMS (ESI) Calcd for C_13_H_12_N_3_O [M + H]^+^ 226.0975; Found 226.0979.

2-(Thiophen-2-yl)quinazolin-4(3H)-one (**3x**) [84]. A yield of 0.291 g (98%), 14.5 h, pale yellow solid. Mp. = 287 °C (EtOH) (lit. [84] 246–248 °C). ^1^H NMR (400 MHz, DMSO-*d*_6_) δ = 12.58 (br.s, 1H), 8.23 (d, *J* = 2.8 Hz, 1H), 8.13–8.12 (m, 1H), 7.85–7.78 (m, 2H), 7.66–7.64 (m, 1H), 7.50–7.46 (m, 1H), and 7.24–7.22 (m, 1H) ppm; ^13^C{^1^H} NMR (100 MHz, DMSO-*d*_6_) δ = 161.7, 148.5, 147.8, 137.3, 134.5, 132.0, 129.3, 128.3, 126.8, 126.2, 125.9, and 120.8 ppm.

2-(Pyridin-4-yl)quinazolin-4(3H)-one (**3y**) [84]. A yield of 0.044 g (15%), 7.5 h, white solid. Mp. = 276–277 °C (EtOH) (lit. [84] 270–272 °C). ^1^H NMR (400 MHz, DMSO-*d*_6_) δ = 12.69 (br.s, 1H), 8.78 (d, *J* = 4.4 Hz, 2H), 8.18 (br.d, *J* = 7.6 Hz, 1H), 8.11 (d, *J* = 4.4 Hz, 2H), 7.88–7.84 (m, 1H), 7.79–7.77 (m, 1H), and 7.59–7.55 (m, 1H) ppm; ^13^C{^1^H} NMR (100 MHz, DMSO-*d*_6_) δ = 161.9, 150.4, 150.1 (2C), 148.2, 139.8, 134.6, 127.6, 127.2, 125.8, 121.4 (2C), and 121.4 ppm.

2-(Naphthalen-2-yl)quinazolin-4(3H)-one (**3z**) [83]. A yield of 0.301 g (85%), 9 h, pale yellow solid. Mp. = 285–286 °C (EtOH) (lit. [83] 285–287 °C). ^1^H NMR (400 MHz, DMSO-*d*_6_) δ = 12.59 (br.s, 1H), 8.82 (br.s, 1H), 8.32–8.30 (m, 1H), 8.19 (d, *J* = 7.6 Hz, 1H), 8.08–8.06 (m, 2H), 8.02–8.00 (m, 1H), 7.88–7.84 (m, 1H), 7.81–7.79 (m, 1H), 7.66–7.61 (m, 2H), and 7.56–7.52 (m, 1H) ppm; ^13^C{^1^H} NMR (100 MHz, DMSO-*d*_6_) δ = 162.2, 152.2, 148.6, 134.5, 134.1, 132.2, 129.9, 128.8, 128.03, 127.97, 127.8, 127.5, 127.4, 126.8, 126.5, 125.8, 124.4, and 121.0 ppm.

2-Cyclohexylquinazolin-4(3H)-one (**3aa**) [87]. A yield of 0.261 g (88%), 8 h, white solid. Mp. = 222 °C (EtOAc) (lit. [87] 230–231 °C). ^1^H NMR (400 MHz, DMSO-*d*_6_) δ = 11.99 (br.s, 1H), 8.07 (br.d, *J* = 7.6 Hz, 1H), 7.77–7.73 (m, 1H), 7.58 (br.d, *J* = 8.0 Hz, 1H), 7.45–7.42 (m, 1H), 2.61–2.55 (m, 1H), 1.92–1.89 (m, 2H), 1.80–1.77 (m, 2H), 1.69–1.55 (m, 3H), and 1.35–1.20 (m, 3H) ppm; ^13^C{^1^H} NMR (100 MHz, DMSO-*d*_6_) δ = 161.8, 160.6, 148.9, 134.0, 126.9, 125.7, 125.5, 120.9, 42.7, 30.1 (2C), 25.4 (2C), and 25.2 ppm.

3-Amino-2-(1H-indol-3-yl)quinazolin-4(3H)-one (**3ab**). A yield of 0.212 g (59%), 15 h, pale brown solid. Mp. ≥ 235 °C, decomposition (EtOH). ^1^H NMR (400 MHz, DMSO-*d*_6_) δ = 11.78 (br.s, 1H), 8.78–8.75 (m, 2H), 8.15–8.13 (m, 1H), 7.82–7.74 (m, 2H), 7.53–7.50 (m, 1H), 7.46–7.43 (m, 1H), 7.25–7.20 (m, 2H), and 5.97 (s, 2H) ppm; ^13^C{^1^H} NMR (100 MHz, DMSO-*d*_6_) δ = 161.0, 151.0, 147.4, 135.8, 133.8, 133.0, 127.0, 126.7, 125.8, 125.0, 122.8, 122.1, 120.6, 118.4, 111.6, and 107.7 ppm. HRMS (ESI) Calcd for C_16_H_13_N_4_O [M + H]^+^ 277.1084; Found 277.1082.

#### 3.3.5. Synthesis of 2-(5-methylfuran-2-yl)-2,3-dihydroquinazolin-4(1*H*)-one (**6u**) [88]

To a solution of 5-methylfuran-2-carbaldehyde (**1s**) (0.76 mmol, 76 µL) in DMSO (1.0 mL), anthranilamide (**2a**) (0.69 mmol, 94 mg) was added. The reaction mixture was heated at 100 °C for 3.5 h (TLC control). Then, the reaction mixture was poured into H_2_O (50 mL). The formed precipitate was filtered. The product was purified using column chromatography on silica gel using the mixture of petroleum ether/EtOAc (1:1) as an eluent and recrystallized from ethanol. A yield of 0.098 g (62%) was obtained and it was a pale yellow solid. Mp. = 168–169 °C (EtOH) (lit. [88] 177–178 °C). ^1^H NMR (400 MHz, DMSO-*d*_6_) δ = 8.24 (br.s, 1H), 7.61 (br.d, *J* = 7.6 Hz, 1H), 7.25–7.21 (m, 1H), 7.10 (s, 1H), 6.75 (br.d, *J* = 8.4 Hz, 1H), 6.69–6.65 (m, 1H), 6.13 (d, *J* = 3.2 Hz, 1H), 5.98 (br.s, 1H), 5.68 (br.s, 1H), and 2.22 (s, 3H) ppm; ^13^C{^1^H} NMR (101 MHz, DMSO-*d*_6_) δ = 163.1, 152.5, 151.3, 147.1, 133.1, 127.1, 117.1, 114.9, 114.4, 108.0, 106.2, 60.3, and 13.2 ppm.

#### 3.3.6. Synthesis of 2-(thiophen-2-yl)-2,3-dihydro-4*H*-benzo[*e*][1,3]oxazin-4-one (**3ad**) [89]

To a solution of thiophene-2-carbaldehyde (**1u**) (0.25 mmol, 24 μL) in toluene (2 mL), 2-hydroxybenzamide (**1f**) (0.25 mmol, 0.034 g) and TFA (0.25 mmol, 19 μL) were added. The reaction mixture was refluxed for 40 h (TLC control) and cooled to room temperature. The reaction mixture was poured into H_2_O (10 mL) and extracted with EtOAc (3 × 5 mL). The combined organic layers were washed with brine (3 × 10 mL), dried with anhydrous Na_2_SO_4_, and concentrated to dryness. The product was purified using column chromatography on silica gel using the mixture of petroleum ether/EtOAc (5:1) as an eluent. A yield of 0.030 g (52%) was obtained and it was a yellow oil. ^1^H NMR (400 MHz, DMSO-*d*_6_) δ = 9.14 (br.s, 1H), 7.79 (br.d, *J* = 7.6 Hz, 1H), 7.62–7.61 (m, 1H), 7.53–7.49 (m, 1H), 7.28–7.27 (m, 1H), 7.14–7.11 (m, 1H), 7.05–7.03 (m, 2H), and 6.69 (br.s, 1H) ppm; ^13^C{^1^H} NMR (101 MHz, DMSO-*d*_6_) δ = 162.0, 156.1, 140.7, 134.5, 127.8, 127.7, 127.3, 126.7, 122.4, 118.3, 116.9, and 80.5 ppm.

#### 3.3.7. Synthesis of 2-(1-benzoyl-1*H*-indol-3-yl)quinazolin-4(3*H*)-one (**3i**)

To a solution of 2-(1*H*-indol-3-yl)quinazolin-4(3*H*)-one (**3a**) (1.0 mmol, 0.261 g) and triethylamine (2.0 mmol, 279 µL) in dimethylacetamide (2.0 mL) benzoyl chloride (2.0 mmol, 232 µL) was added. The reaction mixture was heated at 150 °C for 4 h (TLC control). Then, the reaction mixture was poured into H_2_O (50 mL). Then, NaHCO_3_ (4.2 mmol, 353 mg) was added portion wise. The reaction mixture was stirred at room temperature for 2 h. The formed precipitate was filtered. The product **3i** was purified using column chromatography on silica gel using the mixture of petroleum ether/EtOAc (2:1) as an eluent and recrystallized from an acetone. A yield of 0.288 g (79%) was obtained and it was a white solid. Mp. = 317–318 °C, decomposition (acetone). ^1^H NMR (400 MHz, DMSO-*d*_6_) δ = 12.45 (br.s, 1H), 8.89–8.87 (m, 1H), 8.76 (s, 1H), 8.38–8.36 (m, 1H), 8.14–8.12 (m, 1H), 7.88–7.82 (m, 4H), 7.79–7.75 (m, 1H), 7.70–7.66 (m, 2H), and 7.52–7.48 (m, 3H) ppm; ^13^C{^1^H} NMR (100 MHz, DMSO-*d*_6_) δ = 168.4, 161.8, 148.8, 148.5, 136.1, 134.4, 133.1, 132.4, 130.9, 129.5 (2C), 128.7 (2C), 127.8, 127.3, 126.3, 125.7, 125.5, 124.6, 123.1, 121.0, 115.6, and 113.9 ppm. HRMS (ESI) Calcd for C_23_H_16_N_3_O_2_ [M + H]^+^ 366.1237; Found 366.1246.

#### 3.3.8. Synthesis of Quinazolin-4(3*H*)-Ones **3b**, **3ah**, **3ai**

To a stirred solution of 2-(1*H*-indole-3-yl)quinazolin-4(3*H*)-one (3a) (0.9 mmol, 0.235 g) in dry DMF (1 mL), sodium hydride (2.7 mmol, 60% dispersion in mineral oil, 0.108 g) was added portion wise. The reaction mixture was stirred at room temperature for 5 min, followed by the addition of a methyl iodide (1.8 mmol, 112 µL). The reaction mixture was stirred for 48 h at ambient temperature (TLC control). The reaction mixture was poured into H_2_O (30 mL). The formed precipitate was filtered. The products were purified using column chromatography on silica gel using the mixture of petroleum ether/EtOAc (3:1) as an eluent and recrystallized from a suitable solvent.

2-(1-Methyl-1H-indol-3-yl)quinazolin-4(3H)-one (**3b**) [83]. A yield of 0.067 g (27%), white solid. Mp. ≥ 324 °C, decomposition (EtOH, lit. [83] 290–295 °C). All the spectral data of 2-(1-methyl-1*H*-indol-3-yl)quinazolin-4(3*H*)-one (**3b**) were consistent with those described above.

3-Methyl-2-(1-methyl-1H-indol-2-yl)quinazolin-4(3H)-one (**3ah**) [90]. A yield of 0.057 g (22%), white solid. Mp. = 192–193 °C (EtOH, lit. [90] 194–196 °C). ^1^H NMR (400 MHz, DMSO-*d*_6_) δ = 8.16 (dd, *J* = 8.0, 1.2 Hz, 1H), 8.06–8.04 (m, 2H), 7.82–7.78 (m, 1H), 7.66 (d, *J* = 8.0 Hz, 1H), 7.57 (d, *J* = 8.0 Hz, 1H), 7.50–7.46 (m, 1H), 7.32–7.28 (m, 1H), 7.23–7.20 (m, 1H), 3.92 (s, 3H), and 3.69 (s, 3H) ppm; ^13^C{^1^H} NMR (100 MHz, DMSO-*d*_6_) δ = 162.1, 151.8, 147.6, 136.3, 134.0, 132.7, 126.7, 126.6, 126.0, 125.7, 122.2, 121.2, 120.6, 119.2, 110.2, 108.7, 33.6, and 32.8 ppm.

4-Methoxy-2-(1-methyl-1H-indol-3-yl)quinazoline (**3ai**). A yield of 0.052 g (20%), yellow solid. Mp. = 160 °C, decomposition (EtOAc). ^1^H NMR (400 MHz, DMSO-*d*_6_) δ = 8.76–8.74 (m, 1H), 8.36 (s, 1H), 8.09–8.06 (m, 1H), 7.89–7.84 (m, 2H), 7.55–7.48 (m, 2H), 7.30–7.24 (m, 2H), 4.27 (s, 3H), and 3.92 (s, 3H) ppm; ^13^C{^1^H} NMR (100 MHz, DMSO-*d*_6_) δ = 165.7, 158.7, 151.4, 137.7, 134.0, 133.7, 126.7, 126.1, 125.2, 123.0, 122.2, 121.9, 120.7, 113.9, 113.7, 110.2, 53.9, and 32.8 ppm. HRMS (ESI) Calcd for C_18_H_16_N_3_O [M + H]^+^ 290.1288; Found 290.1279.

#### 3.3.9. Synthesis of 2-(1*H*-indol-3-yl)-4-(methylthio)quinazoline (**3ak**)

To a solution of 2-(1*H*-indol-3-yl)quinazolin-4(3*H*)-one (**3a**) (0.5 mmol, 131 mg) in toluene (5 mL), Lawesson’s reagent (1.5 mmol, 606 mg) was added. The reaction mixture was refluxed for 4 h (TLC control) and cooled to room temperature. The mixture was treated with 1N sodium hydroxide (5 mL) and extracted with EtOAc (3 × 10 mL). The combined organic layers were washed with brine (3 × 10 mL), dried with anhydrous Na_2_SO_4_, and concentrated to dryness. To a solution of 2-(1*H*-indol-3-yl)quinazoline-4(3*H*)-thione (**3aj**) in acetone (2 mL), K_2_CO_3_ (0.6 mmol, 0.083 g) and MeI (0.6 mmol, 37 μL) were added. The reaction mixture was stirred for 2 h at room temperature (TLC control). The reaction was poured into H_2_O (100 mL) and extracted with EtOAc (3 × 20 mL). To the reaction, H_2_O (2 mL) was added and extracted with EtOAc (3 × 20 mL). The combined organic layers were washed with brine (3 × 10 mL), dried with anhydrous Na_2_SO_4_, and concentrated to dryness. The product was purified using column chromatography on silica gel using the mixture of petroleum ether/EtOAc (19:1) as an eluent and recrystallized from a mixture of petroleum ether/EtOAc. A yield of 0.076 g (52%), yellow solid. Mp. = 182–184 °C (petroleum ether/EtOAc). ^1^H NMR (400 MHz, DMSO-*d*_6_) δ = 11.67 (br.s, 1H), 8.76–8.73 (m, 1H), 8.40 (d, *J* = 2.8 Hz, 1H), 8.02 (d, *J* = 8.4 Hz, 1H), 7.93–7.86 (m, 2H), 7.55–7.50 (m, 2H), 7.23–7.21 (m, 2H), and 2.84 (s, 3H) ppm; ^13^C{^1^H} NMR (100 MHz, DMSO-*d*_6_) δ = 169.6, 158.3, 148.4, 137.2, 133.9, 130.3, 127.8, 125.7, 125.6, 123.4, 122.0, 121.9, 120.9, 120.4, 114.9, 111.9, and 12.0 ppm. HRMS (ESI) Calcd for C_17_H_14_N_3_S [M + H]^+^ 292.0903; Found 292.0894. 

#### 3.3.10. Synthesis of 4-Chloro-2-(1H-indol-3-yl)quinazoline (3am)

To a solution of 2-(1*H*-indol-3-yl)quinazolin-4(3*H*)-one (**3a**) (1.0 mmol, 0.261 g) in dry DMF (4.5 mL), POCl_3_ (9.0 mmol) was added at 0 °C. The reaction was stirred at ambient temperature for 12 h (TLC control). Then, the reaction mixture was poured into H_2_O (100 mL). Then, NaHCO_3_ (10.0 mmol, 0.840 g) was added portion wise. The reaction mixture was stirred at room temperature for 2 h. The formed precipitate was filtered. The product was purified using column chromatography on silica gel using the mixture of petroleum ether/EtOAc (2:1) as an eluent and recrystallized from a mixture of petroleum ether/EtOAc. A yield of 0.128 g (46%), pale pink solid. Mp. = 217 °C (petroleum ether/EtOAc). ^1^H NMR (400 MHz, DMSO-*d*_6_) δ = 9.54 (br.s, 1H), 8.77–8.74 (m, 2H), 8.29–8.21 (m, 2H), 8.13–8.06 (m, 2H), 7.80–7.77 (m, 1H), and 7.51–7.45 (m, 2H) ppm; ^13^C{^1^H} NMR (100 MHz, DMSO-*d*_6_) δ = 161.1, 156.6, 150.9, 135.7 (2C), 128.7 (2C), 128.1 (2C), 127.6, 125.5 (2C), 125.3, 124.8, 122.7, and 121.3 ppm. HRMS (ESI) Calcd for C_16_H_11_ClN_3_ [M + H]^+^ 280.0636; Found 280.0635.

#### 3.3.11. Synthesis of 4-[2-(1*H*-indol-3-yl)quinazolin-4-yl]morpholine (**3an**)

To a solution of 4-chloro-2-(1*H*-indol-3-yl)quinazoline (**3am**) (1.0 mmol, 279 mg) and Et_3_N (1.5 mmol, 202 µL) in MeCN (5 mL), morpholine (1.2 mmol, 103 µL) was added. The reaction mixture was refluxed for 1.5 h (TLC control). Then, the reaction mixture was poured into H_2_O (50 mL) and the product was extracted with dichloromethane (3 × 20 mL). The combined organic layers were washed with brine (3 × 20 mL), dried with anhydrous Na_2_SO_4_, and concentrated to dryness under reduced pressure. The product was purified using column chromatography on silica gel using the mixture of petroleum ether/EtOAc (10:1) as an eluent. A yield of 0.261 g (79%), beige oil. ^1^H NMR (400 MHz, DMSO-*d*_6_) δ = 11.58 (br.s, 1H), 8.72–8.68 (m, 1H), 8.26 (d, *J* = 2.8 Hz, 1H), 7.97 (br.d, *J* = 7.6 Hz, 1H), 7.84 (dd, *J* = 8.4, 0.8 Hz, 1H), 7.78–7.74 (m, 1H), 7.50–7.46 (m, 1H), 7.44–7.39 (m, 1H), 7.21–7.16 (m, 2H), 3.89–3.87 (m, 4H), and 3.77–3.75 (m, 4H) ppm; ^13^C{^1^H} NMR (100 MHz, DMSO-*d*_6_) δ = 164.0, 158.4, 152.2, 137.1, 132.5, 129.5, 127.6, 125.8, 125.0, 123.9, 122.1, 121.6, 120.2, 115.3, 114.1, 111.8, 66.0 (2C), and 50.0 (2C) ppm. HRMS (ESI) Calcd for C_20_H_19_N_4_O [M + H]^+^ 331.1553; Found 331.1559.

### 3.4. Antibacterial Activity

The synthesized compounds were tested for their in vitro growth inhibitory and bactericidal (fungicidal) activity against *Staphylococcus aureus* (ATCC 25923), *Staphylococcus aureus* (MRSA, ATCC 43300), *Staphylococcus epidermidis* (ATCC 12228), *Escherichia coli* (ATCC 8739), *Escherichia coli* (ATCC 25922), *Mycobacterium smegmatis* (mc(2)155/ ATCC 700084), and *Candida albicans* (ATCC 10231). Cefazolin, cefotaxime, chloramphenicol, amikacin, rifampicin, isoniazid, fluconazole, and Amphotericin B were used as control antimicrobial (fungicidal) agents. The cells were grown overnight at 37 °C in a glass tube with 5 mL of LB-broth containing 1% of glucose for *C. albicans* and 0.05% Tween 80 for *M. smegmatis*. The grown cultures were then diluted 1:100 with fresh medium and cultivated for 5 h. The *M. smegmatis* culture was grown for 20 h with agitation on a shaker GFL1092 (GFL, Germany) (200 rpm, 37 °C). Then, the cultures were adjusted to OD 0.1 (A625) and diluted to 1:10 for *M. smegmatis* and 1:100 for the other microorganisms. The resulting suspension was used to determine the MIC using the serial dilutions method in 96-well plates with modifications. The synthesized compounds were dissolved in DMSO at a concentration of 20 mg/mL and a series of two-fold dilutions was prepared in the same solvent. Then, 10 μL of the solutions was added to the wells of the plate containing 190 μL of the cell suspension. To the control wells, 10 µL of DMSO was added. The plates were incubated at 37 °C under static conditions for 24 h (72 h for *M. smegmatis*) for the minimum inhibitory concentration determination. The minimum bactericidal concentration and MFC were determined as the lowest concentration of an antimicrobial agent required to achieve a 99.9% reduction in the initial inoculum. In total, 10 µL from each well of a plate for the MIC determination was inoculated on Petri dish with LB-agar (containing 1% of glucose for *C. albicans*), and colonies were checked for after incubation (24 h, 37 °C) to determine the MBC or MFC. The antitubercular activity of the synthesized compounds was studied with the *M. tuberculosis* H_37_Rv strain using the standard BASTEC MGIT 960 growth system (Becton Dickinson) [50]; 10 mg of the test substances was dissolved in 1 mL of DMSO, then mixed with Middlebrook 7H9 medium in a ratio of 1:9 and added to MGIT tubes, obtaining a series of two-fold dilutions with final concentrations from 1 to 100 μg/mL. The tubes initially contained 7 mL of Middlebrook Broth 7H9, 0.8 mL of MGIT OADC supplement, and oxygen-quenched fluorochrome, which changes the fluorescence as microorganisms grow. A total of 0.5 mL of a suspension of *M. tuberculosis* H_37_Rv in physiological saline containing 5 × 10^7^ cells/mL was added to each tube. All the tubes were incubated at 37 °C for 41 days and examined for fluorescence daily.

### 3.5. Biofilm Assay

Biofilm formation was measured using the classical crystal violet test in microtiter plates [91]. The cells were grown overnight at 37 °C in a glass tube with 5 mL of LB-broth. The grown culture was then adjusted to OD 0.1 (A625) and added to the wells of the plates at 200 μL and cultivated for 24 h at 37 °C under static conditions. After that, the wells were washed twice with distilled water, stained for 30 min with 0.1% crystal violet, and washed three times. The dye was extracted with 95% ethanol over 30 min and the optical density (A570) was measured with microplate reader Tecan Infinite M200Pro (Tecan, Austria).

### 3.6. Cytotoxicity Activity

#### 3.6.1. Cell Lines and Culture Conditions

The human cell lines MCF7′, A549, VA13, and HEK293T were maintained in DMEM/F-12 media containing 10% FBS, 50u/mL of penicillin, and 0.05 mg/mL of streptomycin (all products were from Thermo Fisher Scientific, Waltham, MA, USA) at 37 °C in 5% CO_2_. The cell cultures were genotyped using STR and tested for the absence of mycoplasma.

#### 3.6.2. Cytotoxicity Measurements (Mosmann Assay)

The measurements were carried out using the Mosmann assay (MTT) [92]; 2500 cells per well for the A549, HEK293T, and MCF7′ cell lines or 4000 cells per well for the VA13 cell line were plated out in 135 µL of DMEM-F12 (Gibco) media in a 96-well plate. The cells were incubated in a 5% CO_2_ incubator for the first 16 h without being treated. Then, 15 µL of media-DMSO solutions or suspensions of the tested substances were added to the cells (final DMSO concentrations in the media were 1% or less) and treated cells at 72 h (triplicate each). The MTT reagent then was added to the cells up to a final concentration of 0.5 g/L (10× stock solution in PBS was used) and incubated for 3 h at 37 °C in the incubator, under an atmosphere of 5% CO_2_. The MTT solution was then discarded and 140 µL of DMSO was added. The plates were swayed on a shaker (80 rpm) to solubilize the formazan. The absorbance was measured using a microplate reader (VICTOR X5 Plate Reader) at a wavelength of 555 nm to measure the formazan concentration and corresponding quantity of cells. The results were used to construct a dose–response graph and estimate the CC_50_ value using the Prism software (GraphPad Software, Boston, MA, USA). Both the IC_50_ (half of the maximal inhibition) and IC_50abs_ (half of the absolute quantity of cells) were calculated.

### 3.7. Molecular Docking

Molecular docking was carried out using the Schrödinger Maestro software [93]. Our analysis was based on the protein structures of Rel_Mtb_ from *M. tuberculosis* (PDB 5XNX) and Rel_Seq_ from *Streptococcus dysgalactiae subsp. equisimilis* (PDB 1VJ7), which were optimized and minimized with Protein Preparation Wizard and OPLS3 force field. Hydrogen atoms and disulfide bonds were added and water molecules were removed. LigPrep was used to prepare the ligands: ionization states (in pH range of 7 ± 2) and tautomers of the molecules were generated and the chirality of the molecules was determined based on their three-dimensional structure. Only the strongest energy binding isomersation state or tautomer is demonstrated in results for each compound. The Rel_Mtb_ and Rel_Seq_ protein structures were aligned and the 15 Å cubic binding region was centered relative to the crystallized GDP substrate. Receptor grids were generated for both proteins. The standard precision (SP) docking method was applied with default parameters.

## 4. Conclusions

In summary, a series of 2-(1*H*-indol-3-yl)quinazolin-4(3*H*)-one **3** and analogues was synthesized. The analysis of their biological activity showed that some target compounds have antibacterial properties, ranging from good to outstanding. Thus, compound **3k** showed an activity comparable to that of standard antimicrobial agents against *C. albicans*, *S. aureus*, and *S. epidermidis* and a high activity against MRSA. This compound also showed activity against *M. tuberculosis*. In addition, the ability of **3k** to prevent the formation of bacterial biofilms in sublethal concentrations was found. The evaluation of their cytotoxic activity showed that some compounds, **3c**, **3f**, **3g**, **3k**, **3r**, and 3**z,** exhibited significant antiproliferative activity against all the cancer cell lines studied. As a result of the study of in silico compounds, it was shown that the set of synthesized indole derivatives contained compounds with high enough negative values of the binding energy (−∆G) in both protein models used for the molecular docking, which demonstrates their potential ability for the high affinity binding of alarmone synthetases nearest to their active centers. This opens up the possibility of detecting representatives of substituted indoles that can demonstrate an ability to suppress persister cell formation through the inhibition of alarmone synthetases that are synthesized during the transition to the stationary phase.

## Figures and Tables

**Table 1:** Selected antimicrobial data (MIC and MBC/MFC, µg/mL) for the quinazolinone derivatives **3**, **5a**, and **6u** ^a^.

Compounds	*C. a.* 10231 ^b^		*M. s.* 70084 ^c^		*E. c.* 25922 ^d^		*E. c.* 8739 ^e^		*S. a.* 25923 ^f^		MRSA ^g^	
	MIC	MFC	MIC	MBC	MIC	MBC	MIC	MBC	MIC	MBC	MIC	MBC
**3d**	125	125	- ^h^	-	-	-	-	-	-	-	-	-
**3g**	-	-	-	-	-	-	-	-	-	-	-	-
**3k**	7.80	7.80	-	-	-	-	-	-	3.90	7.80	0.98	3.90
**3p**	250	>500	>500	>500	>500	>500	>500	>500	>500	>500	>500	>500
**3t**	125	125	-	-	-	-	-	-	-	-	-	-
**3u**	125	500	-	-	-	-	-	-	-	-	-	-
**3z**	>250	>250	>250	>250	>250	>250	>250	>250	>250	>250	>250	>250
**3ab**	500	-	-	-	-	-	-	-	-	-	-	-
**3ai**	62.50	-	-	-	-	-	-	-	-	-	-	-
**5a**	250	500	-	-	-	-	-	-	-	-	-	-
**6u**	125	-	-	-	-	-	-	-	-	-	-	-
Cefotaxime	n.d. ^i^	n.d.	n.d.	n.d.	0.038	0.038	0.038	0.038	0.31	0.61	19.53	39.06
Cefazolin	n.d.	n.d.	n.d.	n.d.	2.44	2.44	2.44	2.44	0.15	0.61	9.77	39.06
Amikacin	n.d.	n.d.	n.d.	n.d.	19.53	19.53	19.53	19.53	4.88	9.77	9.77	9.77
Fluconazole	1.94	7.8	n.d.	n.d.	n.d.	n.d.	n.d.	n.d.	n.d.	n.d.	n.d.	n.d.
Isoniazid	n.d.	n.d.	4.58	9.16	n.d.	n.d.	n.d.	n.d.	n.d.	n.d.	n.d.	n.d.
Rifampicin	n.d.	n.d.	1.22	19.53	n.d.	n.d.	n.d.	n.d.	n.d.	n.d.	n.d.	n.d.

^a^ In the table, the Mode values from 3–5 independent experiments are presented; ^b^ *Candida albicans* ATCC 10231; ^c^ *Mycobacterium smegmatis* ATCC 70084; ^d^ *Escherichia coli* ATCC 25922; ^e^ *Escherichia coli* ATCC 8739; ^f^ *Staphylococcus aureus* ATCC 25923; ^g^ *Staphylococcus aureus* ATCC 43300 (MRSA); ^h^ (-) >1000 µg/mL; ^i^ n.d.—not determined.

**Table 2:** Activity of indolylquinazolinone **3k** against *Mycobacterium tuberculosis* H_37_Rv.

Conc., µg/mL	Fluorescence Change (BACTEC MGIT 960 Growth System), Days															
	4	5	6	7	8	9	10	11	12	13	14	18	25	30	35	41
Control	+	+	+	+	+	+	+	+	+	+	+	+	+	+	+	+
100.0	-	-	-	-	-	-	-	-	-	-	-	-	-	-	-	-
50.0	-	-	-	-	-	-	-	-	-	-	-	-	-	-	-	-
25.0	-	-	-	-	-	-	-	-	-	-	-	-	-	-	-	-
10.0	-	-	-	-	-	-	-	-	-	-	-	-	-	-	-	-
5.0	-	-	-	-	-	-	-	-	-	-	-	-	+	+	+	+
2.5	-	-	+	+	+	+	+	+	+	+	+	+	+	+	+	+
1.0	-	-	+	+	+	+	+	+	+	+	+	+	+	+	+	+

Control—*M. tuberculosis* H_37_Rv inoculum without chemical compounds; - lack of growth; + growth of mycobacteria.

**Table 3:** The IC_50_ or IC_50abs_ (µM) for the cytotoxicity of the compounds **3b**–**3g**, **3i**, **3k**, **3o**, **3q**–**3ab**, and **6u** to cells MCF7′, A549, HEK293T, and VA13 via MTT assay ^a^.

Compounds	MCF7′		VA13		A549		HEK293T	
	IC_50_	IC_50abs_	IC_50_	IC_50abs_	IC_50_	IC_50abs_	IC_50_	IC_50abs_
**3b**	2.1 ± 0.4	27.1 ± 4.7	11.4 ± 1.2	29.4 ± 4.3	2.1 ± 0.3	3.6 ± 0.4	6.2 ± 0.7	8.3 ± 0.7
**3c**	4.1 ± 0.6	10.9 ± 1.1	3.7 ± 0.4	4.0 ± 0.2	3.7 ± 0.4	1.7 ± 0.2	1.8 ± 0.7	3.4 ± 0.6
**3d**	le ^b^	x > 100	47.0 ± 11.8	69.3 ± 3.1	43.0 ± 5.1	40.5 ± 2.7	5.5 ± 0.9	56.8 ± 7.9
**3e**	x > 100	x > 100	x > 100	x > 100	~1	8.0 ± 2.3	~1	x > 100
**3f**	4.9 ± 0.7	7.1 ± 0.8	3.4 ± 0.4	3.8 ± 0.3	0,96 ± 0,18	1.3 ± 0.09	0.95 ± 0.28	1.9 ± 0.16
**3g**	0.57 ± 0.05	27.6 ± 7.4	0.84 ± 0.38	13.5 ± 3.6	0.75 ± 0.07	3.3 ± 0,6	0.73 ± 0.15	9.8 ± 2.5
**3i**	11.8 ± 0.7	10.9 ± 0.6	6.6 ± 0.5	7.1 ± 0.4	11.7± 1.2	9.0 ± 0.7	3.4 ± 0.2	4.1 ± 0.3
**3k**	5.9 ± 0.3	6.1 ± 0.3	7.9 ± 0.4	8.5 ± 0.3	6.5 ± 0.2	6.5± 0.2	3.4 ± 0.1	3.5 ± 0.1
**3o**	n.a. ^c^	57.1 ± 6.2	~20	67.8 ± 21.1	9.1 ± 1.2	38.1 ± 6.4	n.a.	19.8 ± 3.4
**3q**	~20	19.7 ± 2.5	16.5 ± 2.5	23.2 ± 2.9	6.1 ± 1.1	6.9 ± 0.6	8.2 ± 1.1	9.0 ± 0.7
**3r**	5.4 ± 2.2	x > 100	2.5 ± 0.7	60.1 ± 23.0	3.5 ± 03	27.6 ± 4.9	1.8 ± 0.4	48.9 ± 13.6
**3s**	13.6 ± 1.2	10.1 ± 1.0	13.5 ± 1.8	12.9 ± 0.8	9.4 ± 03	9.3 ± 0.3	12.4 ± 0.5	12,7 ± 0.4
**3t**	x > 33.3	x > 33.3	x > 33.3	x > 33.3	x > 333	x > 33.3	x > 33.3	x > 33.3
**3u**	0.30 ± 0.14 (le)	x > 100	x > 100	x > 100	n.a.	107.4 ± 27.7	n.a.	66.8 ± 3.6
**3v**	n.a.	93.0 ± 3.1	n.a.	80.6 ± 3.9	n.a.	57.0 ± 3.9	n.a.	62.9 ± 2.8
**3w**	0.53 ± 0.44 (le)	x > 100	x > 100	115.8 ± 0.2	n.a.	105.1 ± 15.8	n.a.	104.8 ± 2.1
**3x**	le	x > 100	le	x > 100	le	x > 100	57.3 ± 34.5	152.3 ± 30.3
**3y**	x > 100	x > 100	x > 100	x > 100	61.4 ± 58.1	x > 100	x > 100	x > 100
**3z**	8.4 ± 0.70	16.1 ± 0.9	3.0 ± 0.4	4.2 ± 0.4	2.6 ± 0.3	3.2 ± 0.3	3.9 ± 0.1	4.0 ± 0.1
**3aa**	x > 100	x > 100	x > 100	163.4 ± 19.7	x > 100	65.5 ± 5.8	x > 100	195.8 ± 51.0
**3ab**	x > 100	x > 100	44.9 ± 11.7	90.8 ± 8.7	n.a.	169.1 ± 47.2	30.5 ± 1.1	32.2 ± 1.6
**6u**	x > 100	x > 100	x > 100	129.3 ± 23.4	x > 100	215.7 ± 35.8	x > 100	x > 100
**doxorubicin**	0.195 ± 0.01	0.195 ± 0.01	0.087 ± 0.04	0.087 ± 0.04	0.038 ± 0.01	0.038 ± 0.01	0.018 ± 0.001	0.018 ± 0.001

^a^ Data over 100 µM—extrapolation result; ^b^ le—the effect does not reach 50%, but there is some cytotoxicity; ^c^ n.a.—not active.

**Table 4:** Docking results for the compounds **3a**–**3g**, **3k**, **3l**, **3o**–**3r**, **3ab**, **3ah**, **3am**, **3ak**, and **7a**, **7b** into Rel_Seq_ and Rel_Mtb_ models reported as docking scores.

Rel_Seq_ SYN Active Site		Rel_Mtb_ SYN Active Site	
Ligand	Docking Score (kcal mol^−1^)	Ligand	Docking Score (kcal mol^−1^)
Indole-5-carboxylic acid (COO^−^)	−6.42	**3b**	−7.61
**3o**	−4.76	**3ab**	−7.43
**3ab**	−4.72	**3p**	−6.71
**3a**	−4.64	**3a**	−6.70
**3b**	−4.53	**3ah**	−6.67
**3d**	−4.48	**3r**	−6.66
**3e**	−4.48	**3am**	−6.62
**3ak**	−4.45	**3o**	−6.56
**7a**	−4.39	Indole-5-carboxylicacid (COO^−^)	−6.55
**3ah**	−4.34	**7a**	−6.52
**3r**	−4.33	**3l**	−6.51
**3p**	−4.32	DMNP ^a^ (COO^−^)	−6.41
DMNP ^a^ (COO^−^)	−4.28	**3k**	−6.17
**3q**	−4.28	**3c**	−6.10
**3g**	−4.15	**3d**	−6.07
**3c**	−4.11	**3q**	−6.05
**3l**	−4.05	**3ai**	−5.82
**3i**	−4.01	**3f**	−5.77
**3ai**	−3.97	**3i**	−5.57
**3f**	−3.84	**3ak**	−5.50
**3am**	−3.78	**3g**	−5.33
**3k**	−3.77	**7b**	−4.96
**7b**	−3.73	**3e**	−4.25

^a^ DMNP—4-(4,7-dimethyl-1,2,3,4-tetrahydronaphthalen-1-yl)pentanoic acid.

## Data Availability

The supporting information includes NMR spectral and HRMS charts.

## Supplementary Materials

The following supporting information can be downloaded at: https://www.mdpi.com/article/10.3390/molecules28145348/s1, Table S1: Antimicrobial data (MIC and MBC/MFC, µg/mL) for the quinazolinone derivatives **3**, **5**, **6**, **7**; Scheme S1: Synthesis of *N*,*N*-dimethyl-1*H*-indole-3-carbothioamides **7a**, **b**; copies of ^1^H, ^13^C NMR spectra of target compounds; copies of HRMS of new compounds; X-ray crystallography data, Table S2: Experimental details for *N*,*N*-dimethyl-1*H*-indole-3-carbothioamide (**7a**, CCDC 2269768); Figure S1. Structure of the *N*,*N*-dimethyl-1*H*-indole-3-carbothioamide (**7a**, CCDC 2269768) according to the X-ray diffraction data; non-hydrogen atoms are shown as thermal vibration ellipsoids with a probability of 50%.
