# Supplementary material for: The Synthesis and Biological Evaluation of 2-(1H-Indol-3-yl)quinazolin-4(3H)-One Derivatives
Source: Molecules. 2023 Jul 11;28(14):5348. doi: 10.3390/molecules28145348 (PMC10384628; doi:10.3390/molecules28145348)
Supplement: Supplementary file 1 [file molecules-28-05348-s001.zip › molecules-2482623-supplementary.pdf]

The Synthesis and Biological Evaluation of 2-(1*H*-Indol-3-yl)quinazolin-4(3*H*)-one Derivatives

Elena Y. Mendogralo [1,*], Larisa Y. Nesterova [1,2], Ekaterina R. Nasibullina [1], Roman O. Shcherbakov [1], Alexander G. Tkachenko [1,2], Roman Y. Sidorov [1,2], Maxim A. Sukonnikov [3], Dmitry A. Skvortsov [3], and Maxim G. Uchuskin [1]

[1] Department of Chemistry, Perm State University, Bukireva St. 15, 614990 Perm, Russia; larisa.nesterova@bk.ru (L.Y.N.); kat.nasibullina@yandex.ru (E.R.N.); romanshcherbakov00@gmail.com (R.O.S.); agtkachenko@iegm.ru (A.G.T.); sidorov.r@iegm.ru (R.Y.S.); mu@psu.ru (M.G.U.)

[2] Institute of Ecology and Genetics of Microorganisms, Ural Branch of the Russian Academy of Sciences, Goleva St. 13, 614081 Perm, Russia

[3] Department of Chemistry, M.V. Lomonosov Moscow State University, Leninskie Gory 1-3, 119991 Moscow, Russia; sukonnikoff.maxim@yandex.ru (M.A.S.); skvorratd@mail.ru (D.A.S.)

* Correspondence: zelina.e@psu.ru

Supporting Information

Table of contents

1. Antimicrobial data (MIC and MBC/MFC, μg/mL) for the quinazolinone derivatives **3**, **5**, **6**, **7**…..S2
2. Synthesis of *N*,*N*-dimethyl-1*H*-indole-3-carbothioamides **7a**, **b**……………………………..…S3
3. Copies of ^{1}H, ^{13}C NMR spectra of target compounds…………………………...…………….S4
4. Copies of HRMS of new compounds …………….…………………………………………...….S39
5. X-ray crystallography data……………………………………………………………………...S48

1. **Table S1**: Antimicrobial data (MIC and MBC/MFC, μg/mL) for the quinazolinone derivatives **3**, **5**, **6**, **7** [a]

Compounds	*C.a.* 10231 [b]		*M. s.* 70084 [c]		*E. c.* 25922 [d]		*E. c.* 8739 [e]		*S. a.* 25923 [f]		MRSA [g]	
	MIC	MFC	MIC	MBC	MIC	MBC	MIC	MBC	MIC	MBC	MIC	MBC
**3a**	-	-	-	-	-	-	-	-	-	-	-	-
**3b**	>50	>50	>50	>50	>50	>50	>50	>50	>50	>50	>50	>50
**3c**	-	-	-	-	-	-	-	-	-	-	-	-
**3d**	125	125	-	-	-	-	-	-	-	-	-	-
**3e**	>50	>50	>50	>50	>50	>50	>50	>50	>50	>50	>50	>50
**3f**	-	-	-	-	-	-	-	-	-	-	-	-
**3g**	-	-	-	-	-	-	-	-	-	-	-	-
**3i**	>250	>250	>250	>250	>250	>250	>250	>250	>250	>250	>250	>250
**3k**	7.80	7.80	-	-	-	-	-	-	3.90	7.80	0.98	3.9
**3l**	-	-	-	-	-	-	-	-	-	-	-	-
**3o**	>500	-	-	-	-	-	-	-	-	-	-	-
**3p**	250	>500	>500	>500	>500	>500	>500	>500	>500	>500	>500	>500
**3q**	-	-	-	-	-	-	-	-	-	-	-	-
**3r**	-	-	-	-	-	-	-	-	-	-	-	-
**3s**	-	-	-	-	-	-	-	-	-	-	-	-
**3t**	125	125	-	-	-	-	-	-	-	-	-	-
**3u**	125	500	-	-	-	-	-	-	-	-	-	-
**3v**	-	-	-	-	-	-	-	-	-	-	-	-
**3w**	-	-	-	-	-	-	-	-	-	-	-	-
**3x**	-	-	-	-	-	-	-	-	-	-	-	-
**3y**	>330	>330	>330	>330	>330	>330	>330	>330	>330	>330	>330	>330
**3z**	>250	>250	>250	>250	>250	>250	>250	>250	>250	>250	>250	>250
**3aa**	-	-	-	-	-	-	-	-	-	-	-	-
**3ab**	500	-	-	-	-	-	-	-	-	-	-	-
**3ah**	>1000	>1000	>1000	>1000	>1000	>1000	>1000	>1000	>1000	>1000	>1000	>1000
**3ai**	62.5	-	-	-	-	-	-	-	-	-	-	-
**3ak**	-	-	-	-	-	-	-	-	-	-	-	-
**3am**	>50	>50	>50	>50	>50	>50	>50	>50	>50	>50	>50	>50
**5a**	250	500	-	-	-	-	-	-	-	-	-	-
**6u**	125	1000	-	-	-	-	-	-	-	-	-	-
**7a**	-	-	-	-	-	-	-	-	-	-	-	-
**7b**	-	-	-	-	-	-	-	-	-	-	-	-
Cefotaxime	n.d.	n.d.	n.d.	n.d.	0.038	0.038	0.038	0.038	0.31	0.61	19.53	39.06
Cefazolin	n.d.	n.d.	n.d.	n.d.	2.44	2.44	2.44	2.44	0.15	0.61	9.77	39.06
Chloramphenicol	n.d.	n.d.	n.d.	n.d.	1.22	4.88	2.44	4.88	9.77	9.77	39.06	39.06
Amikacin	n.d.	n.d.	n.d.	n.d.	19.53	19.53	19.53	19.53	4.88	9.77	9.77	9.77
Fluconazole	1.94	7.8	n.d.	n.d.	n.d.	n.d.	n.d.	n.d.	n.d.	n.d.	n.d.	n.d.
Amphotericin B	0.39	1.56	n.d.	n.d.	n.d.	n.d.	n.d.	n.d.	n.d.	n.d.	n.d.	n.d.
Isoniazid	n.d.	n.d.	4.58	9.16	n.d.	n.d.	n.d.	n.d.	n.d.	n.d.	n.d.	n.d.
Rifampicin	n.d.	n.d.	1.22	19.53	n.d.	n.d.	n.d.	n.d.	n.d.	n.d.	n.d.	n.d.

[a] In the table present the Mode values from 3-5 independent experiments; [b] *Candida albicans* ATCC 10231; [c] *Mycobacterium smegmatis* ATCC 70084; [d] *Escherichia coli* ATCC 25922; [e] *Escherichia coli* ATCC 8739; [f] *Staphylococcus aureus* ATCC 25923; [g] *Staphylococcus aureus* ATCC 43300 (MRSA); [h] (-) >1000 μg/mL; [i] n.d. – not determined.

2. **Scheme S1**. Synthesis of *N*,*N*-dimethyl-1*H*-indole-3-carbothioamides **7a**, **b**

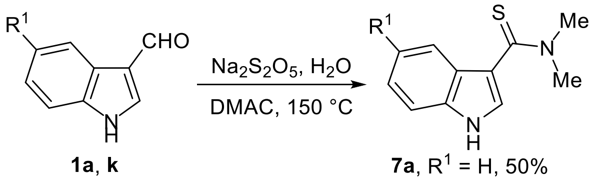

To confirm the process of thioamidation of the starting indole-3-carbaldehydes **1a**, **k** with prolonged heating of the reaction mixture, a control experiment was carried out. To a suspension of aldehyde **1a** or 1**k** (1.3 mmol) and $Na_2S_2O_5$ (4.5 mmol, 0.855 g) in *N*,*N*-dimethylacetamide (2.5 mL) H_2O (4.5 mmol, 81 μL) was added. The reaction mixture was heated at 150 °C for 54 h (TLC control). Then the reaction mixture was poured into H_2O (50 mL). The formed precipitate was filtered. The products **7a,b** were purified by column chromatography on silica gel using the mixture of petroleum ether/EtOAc (10:1) as an eluent and recrystallized from the mixture of petroleum ether/EtOAc.

N,N-dimethyl-1H-indole-3-carbothioamide (**7a**). Yield 0.133 g (50%), pale beige solid. Mp. ≥ 180 °C, decomposition (petroleum ether/EtOAc). All spectral data of *N*,*N*-dimethyl-1*H*-indole-3-carbothioamide (**7a**) are consistent with those described above.

*5-Iodo-N,N-dimethyl-1H-indole-3-carbothioamide (**7b**)*. Yield 0.219 g (51%), pale beige solid. Mp. = 197 – 198 °C (petroleum ether/EtOAc). ^{1}H NMR (400 MHz, DMSO-d_6) δ = 11.67 (br.s, 1H), 8.12 (s, 1H), 7.61 (d, *J* = 2.0 Hz, 1H), 7.41 (br.d, *J* = 8.4 Hz, 1H), 7.28 (br.d, *J* = 8.4 Hz, 1H), 3.42 (br.s, 6H) ppm; $^{13}C\{^{1}H\}$ NMR (100 MHz, DMSO-d_6) δ = 192.1, 134.7, 129.7, 128.9, 127.9, 127.0, 117.0, 114.2, 83.9, 43.4 (br.s, 2C) ppm. HRMS (ESI) Calcd for $C_{11}H_{12}IN_2S$ $[M+H]^+$ 330.9771; Found 330.9763.

3. Copies of ^{1}H, ^{13}C NMR spectra of target compounds

^{1}H, DMSO-d_6, 400 MHz

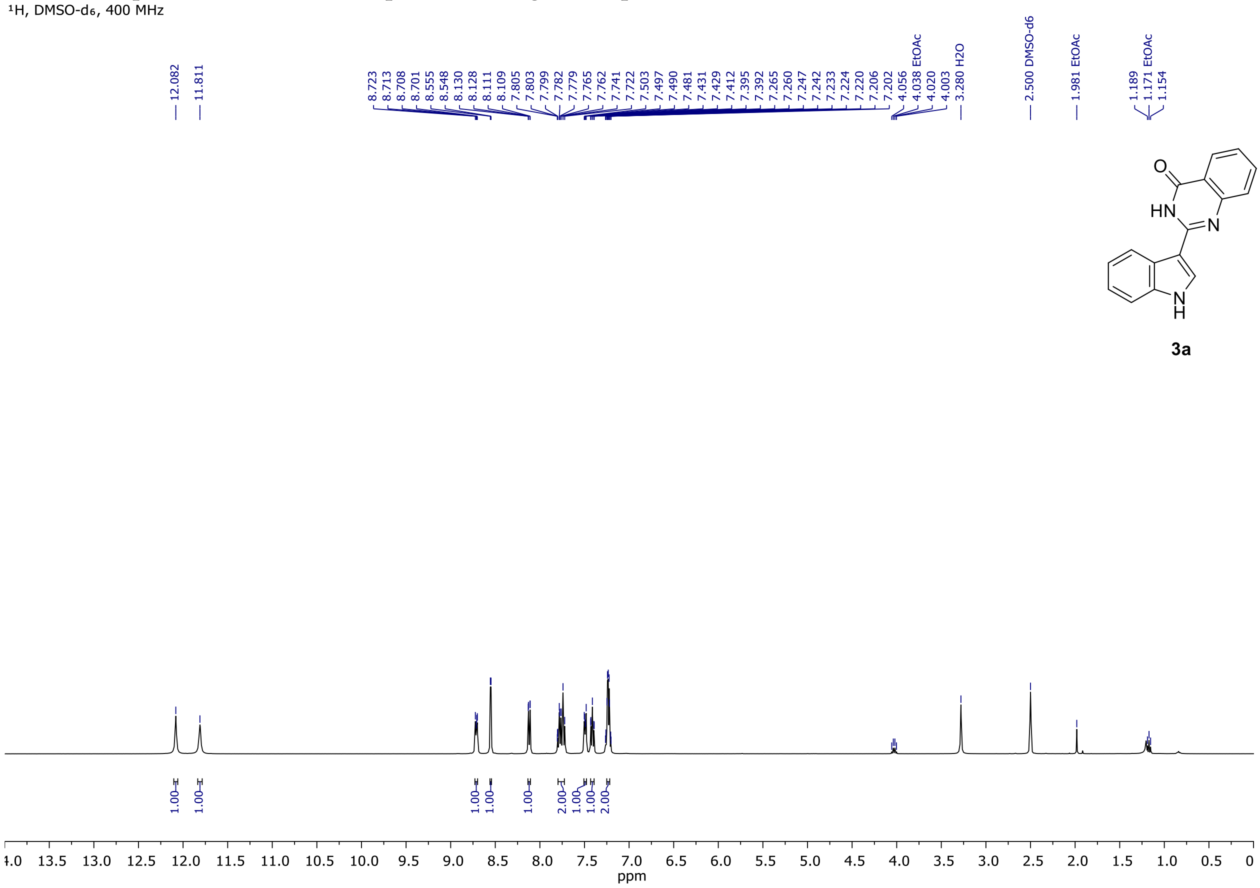

$^{13}C\{^{1}H\}$, DMSO-d_6, 100 MHz

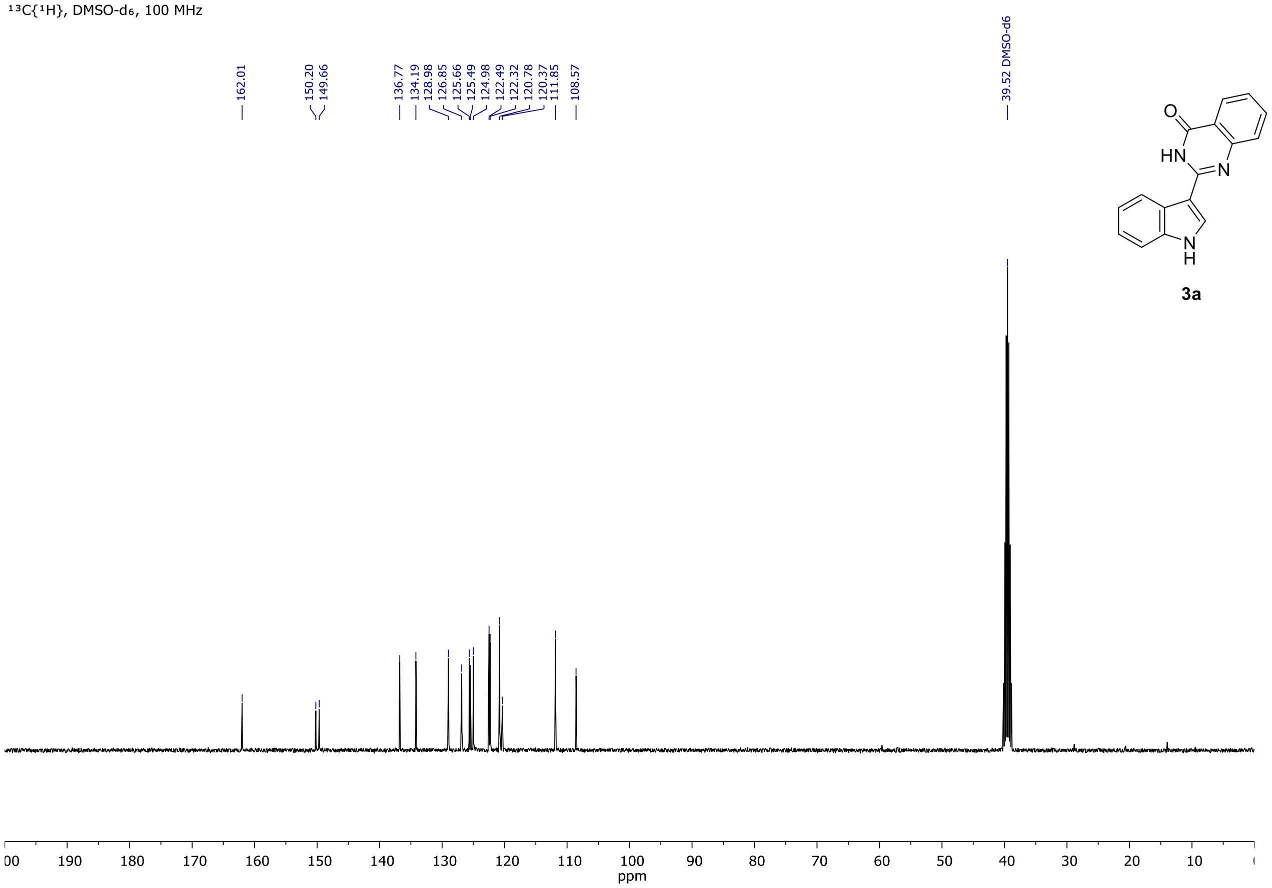

^1H, DMSO-d$_6$, 400 MHz

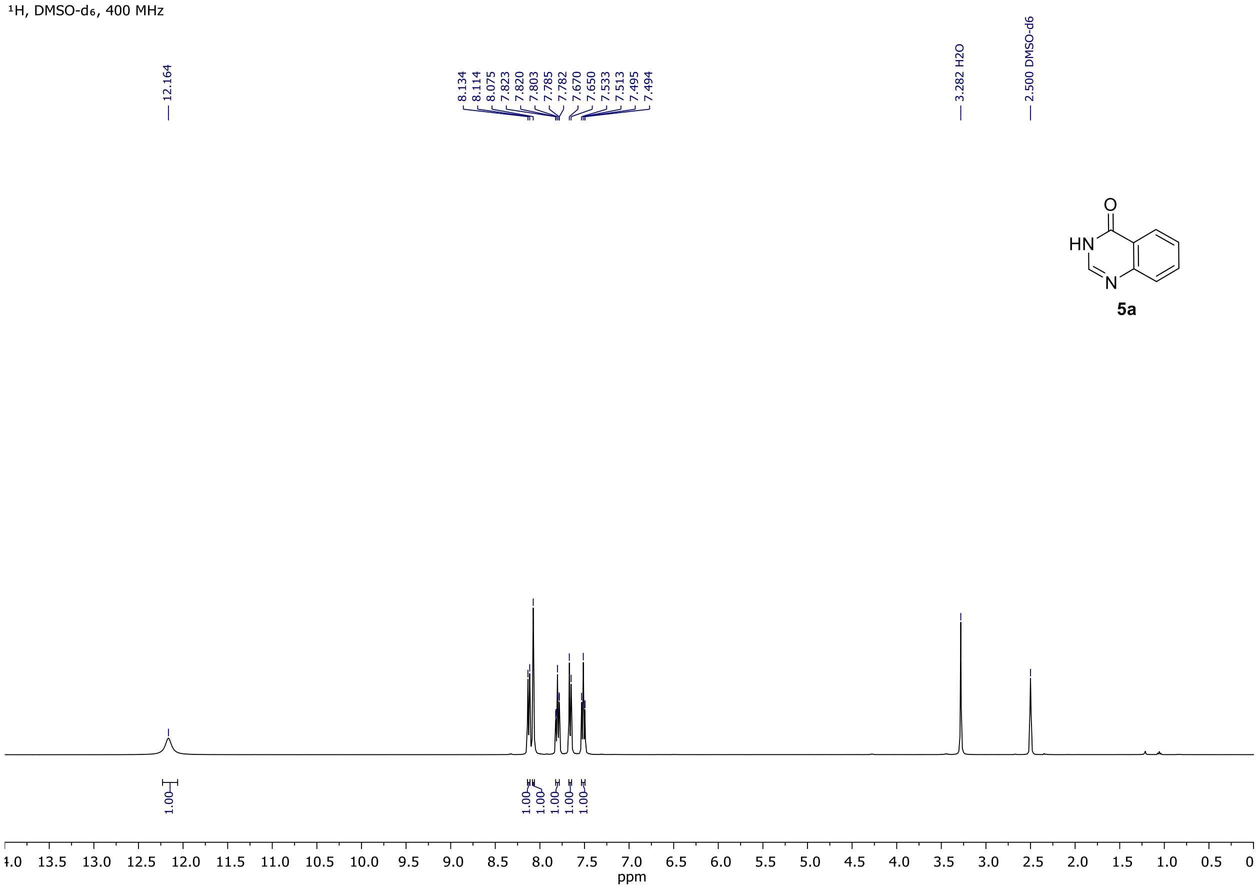

$^{13}C\{^1H\}$, DMSO-d$_6$, 100 MHz

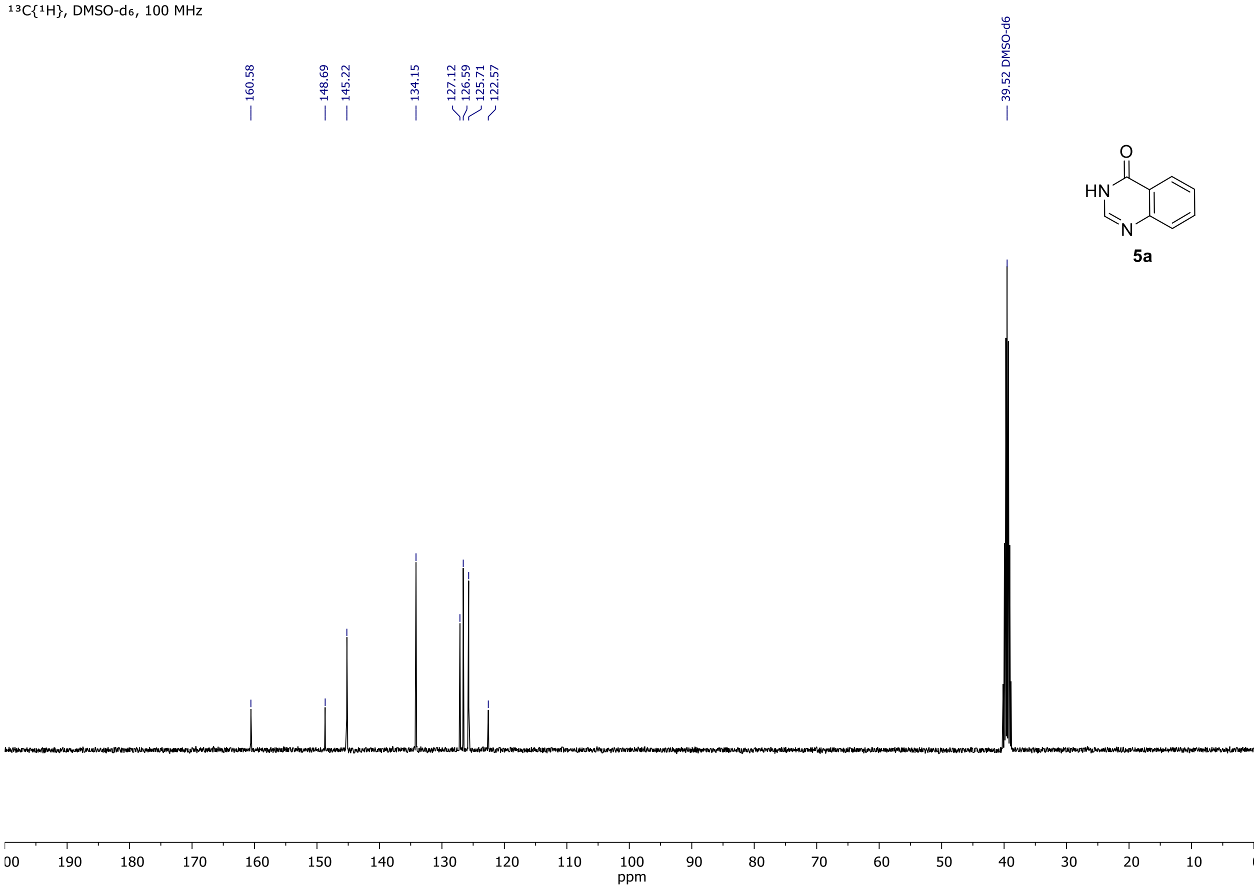

¹H, DMSO-d₆, 400 MHz

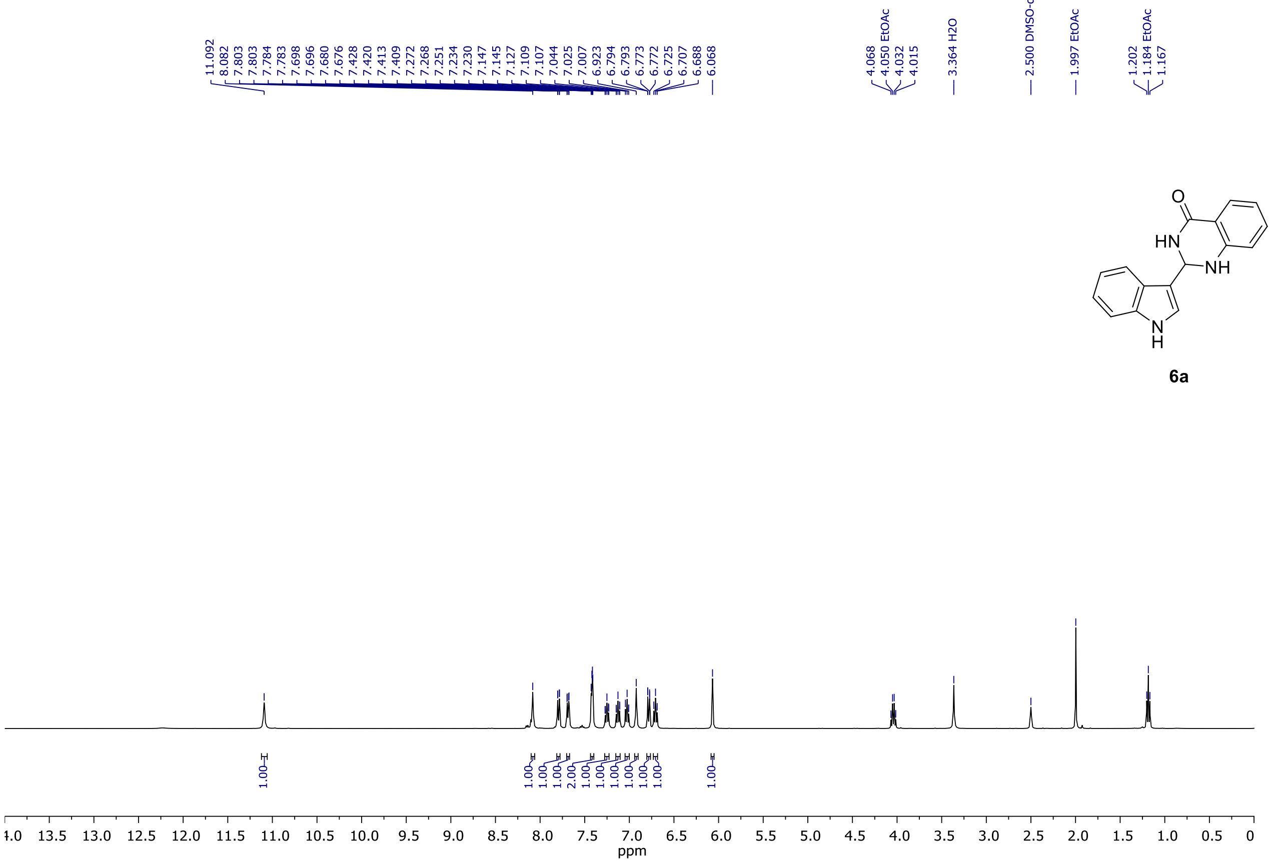

¹³C{¹H}, DMSO-d₆, 100 MHz

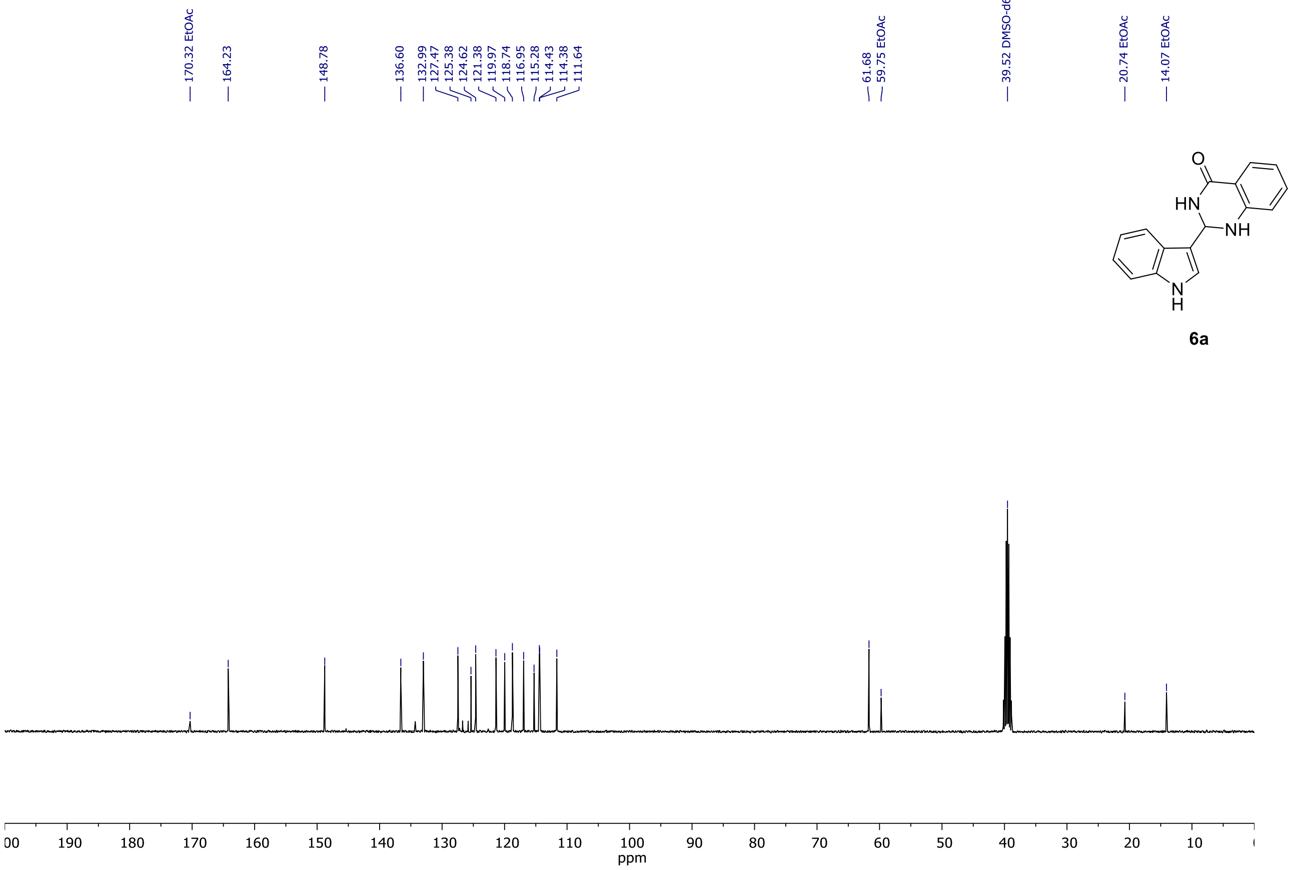

^{1}H, DMSO-d_6, 400 MHz

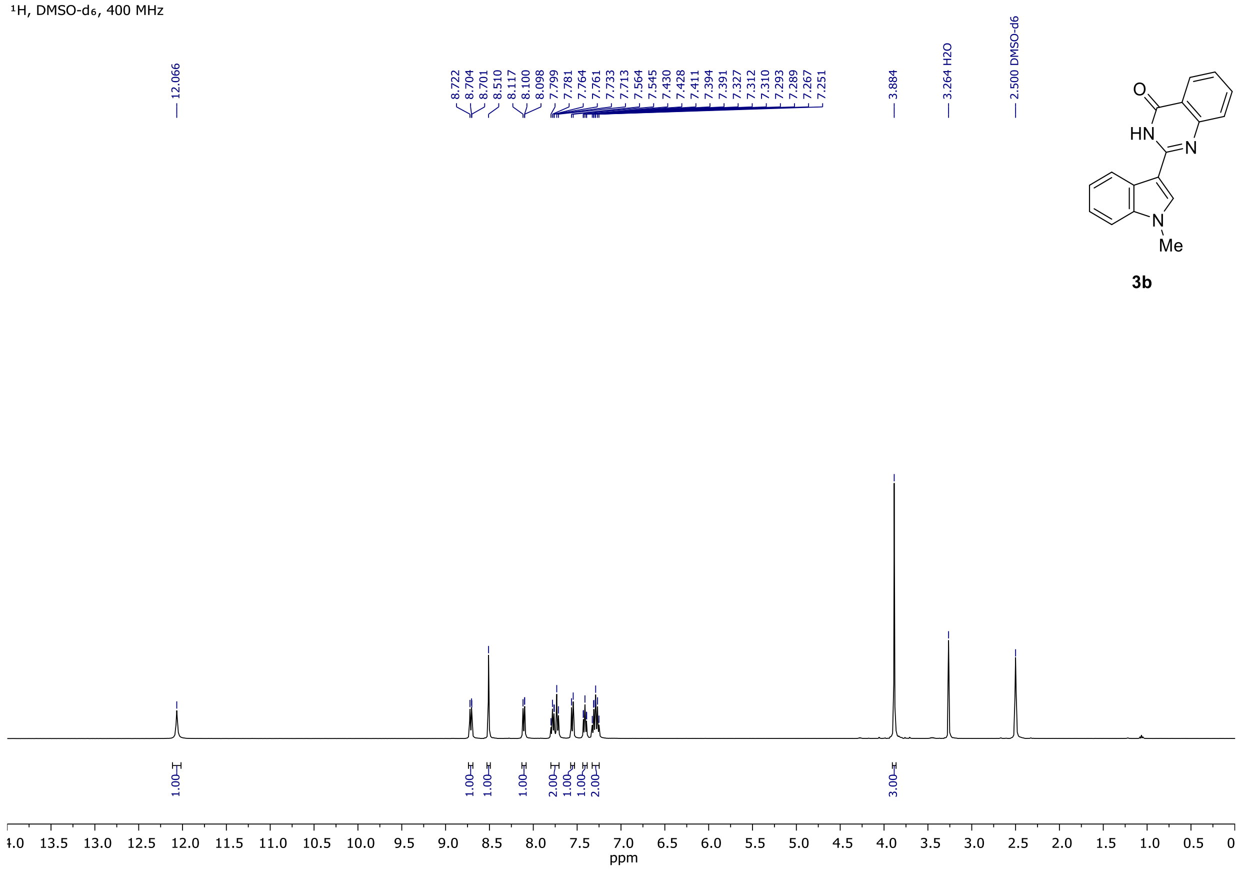

$^{13}C\{^{1}H\}$, DMSO-d_6, 100 MHz

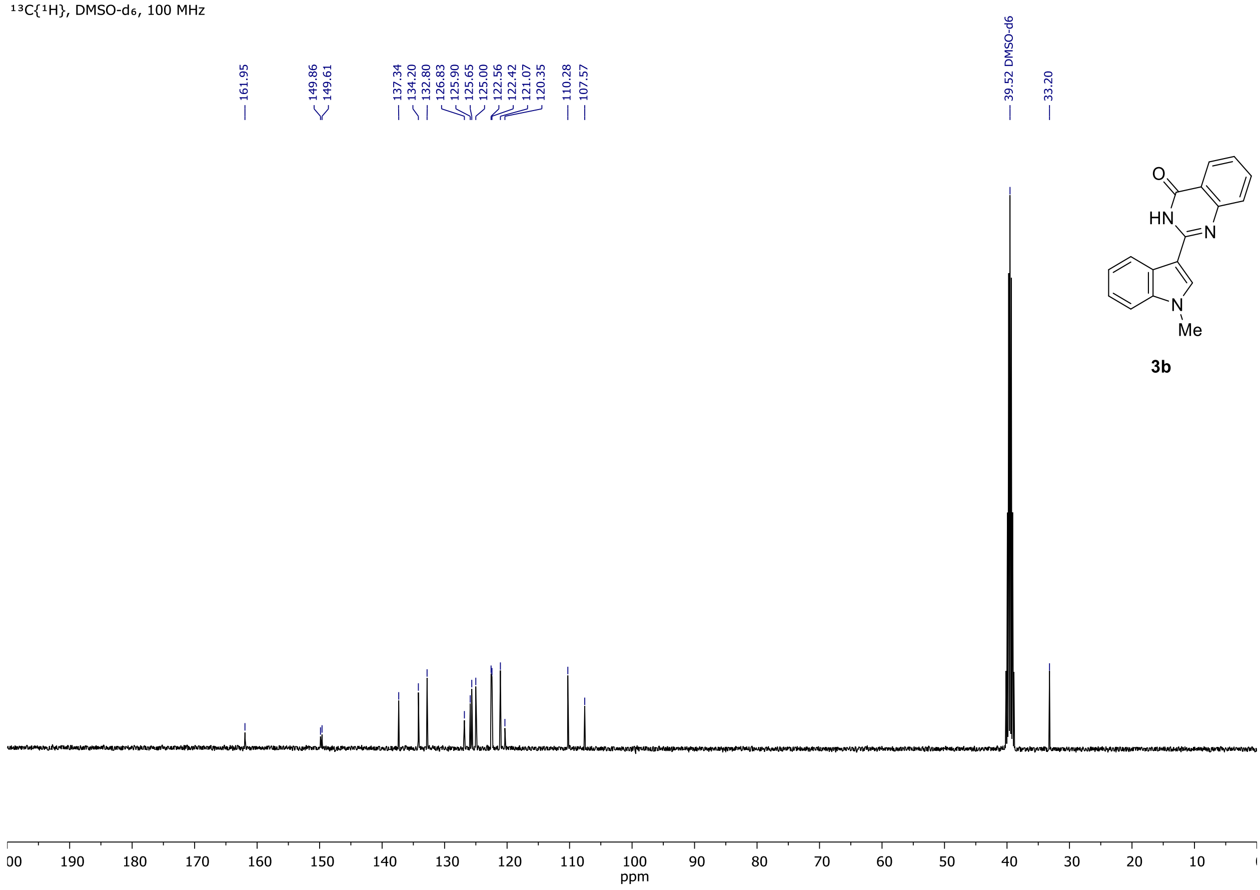

¹H, DMSO-d₆, 400 MHz

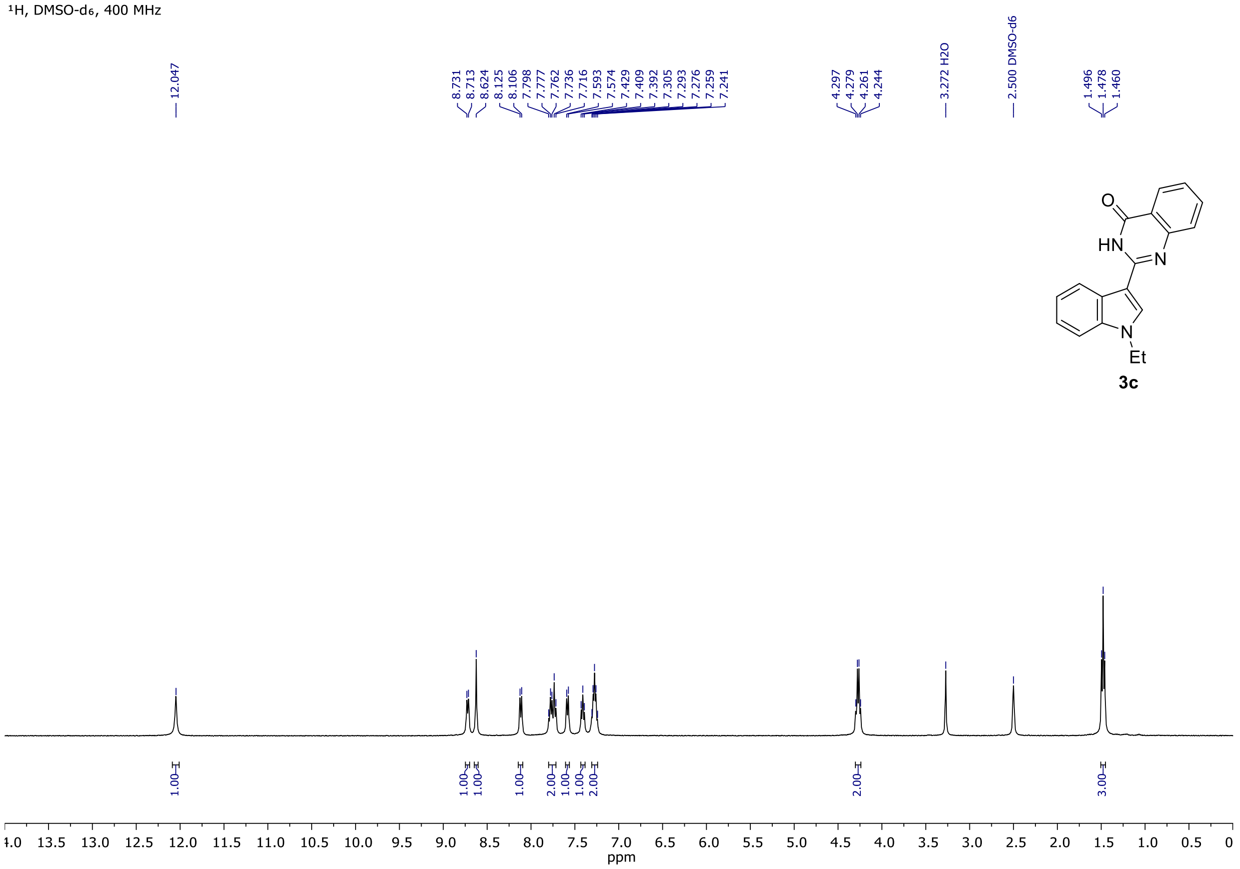

¹³C{¹H}, DMSO-d₆, 100 MHz

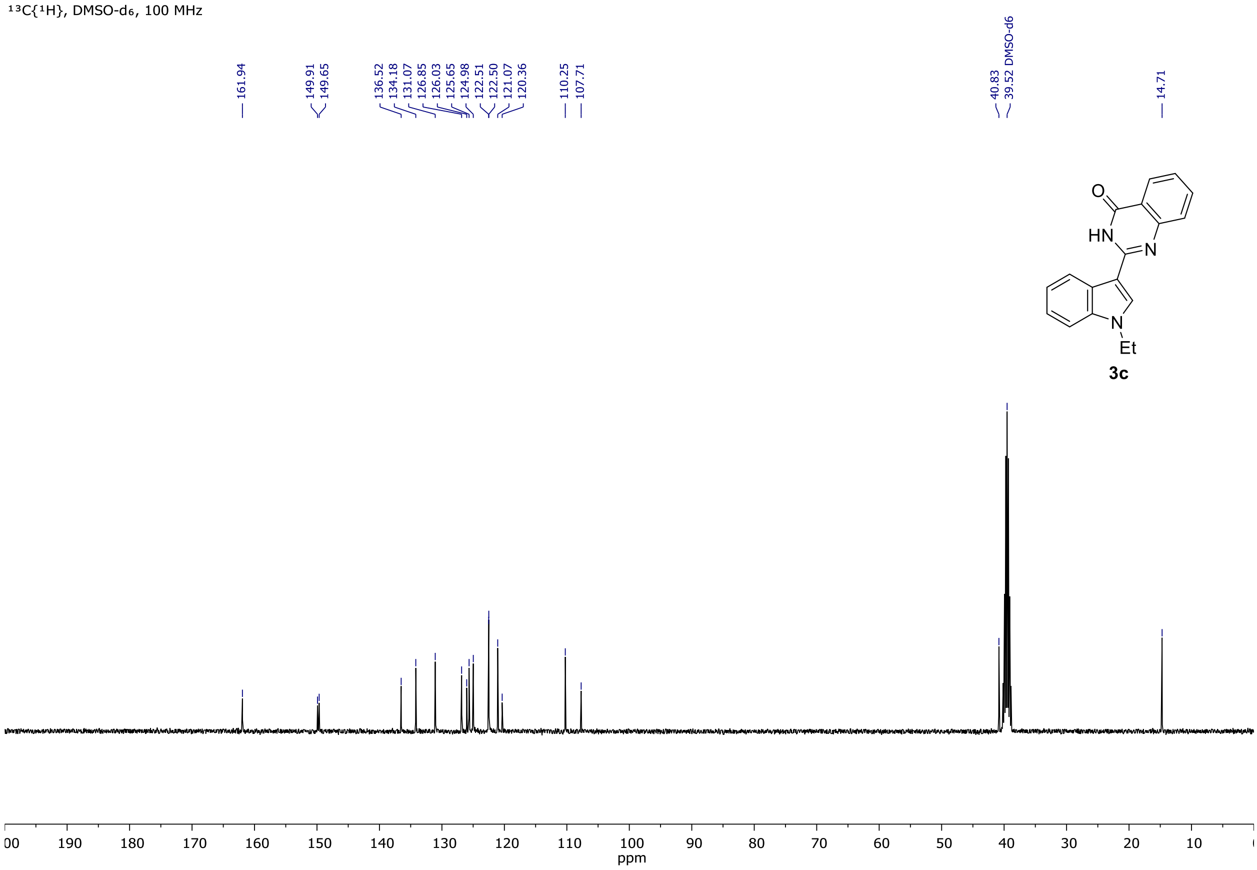

^1H, DMSO-d_6, 400 MHz

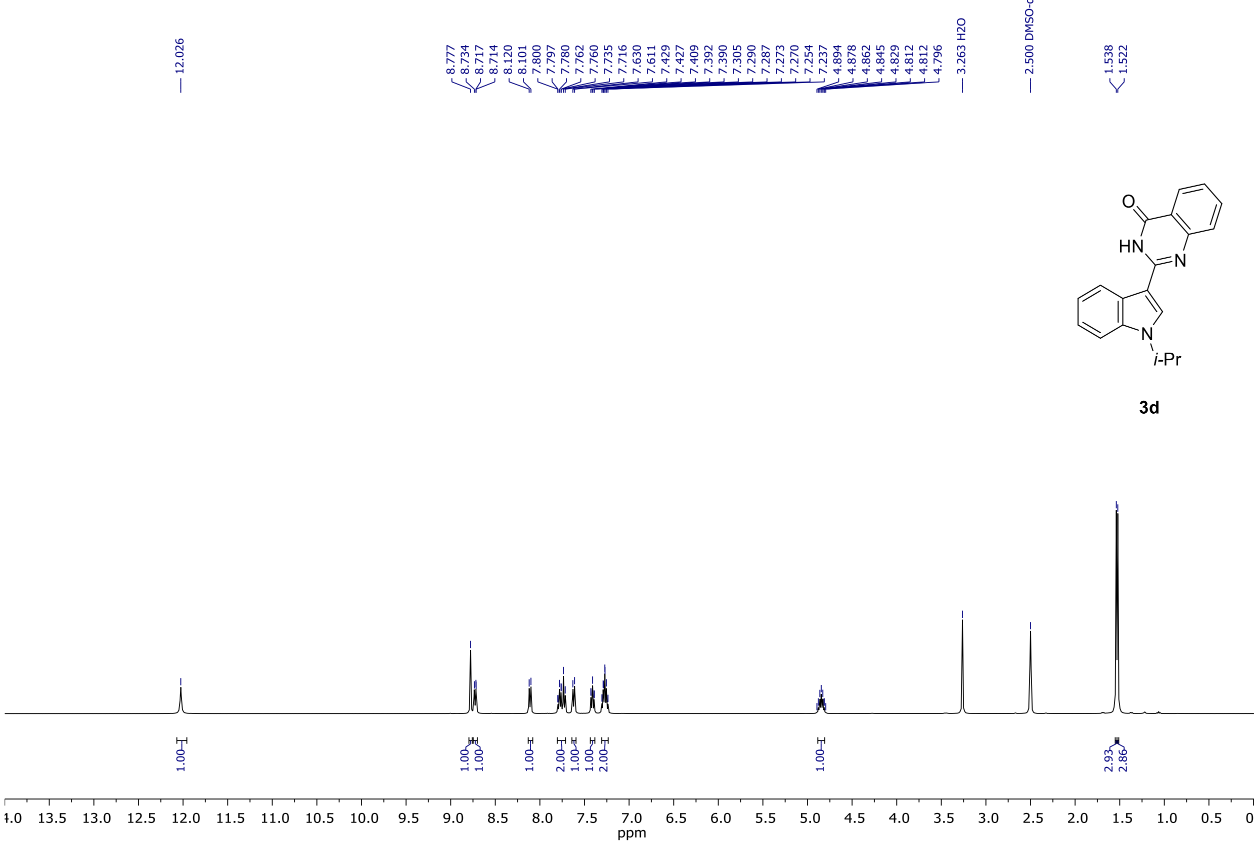

$^{13}C\{^1H\}$, DMSO-d_6, 100 MHz

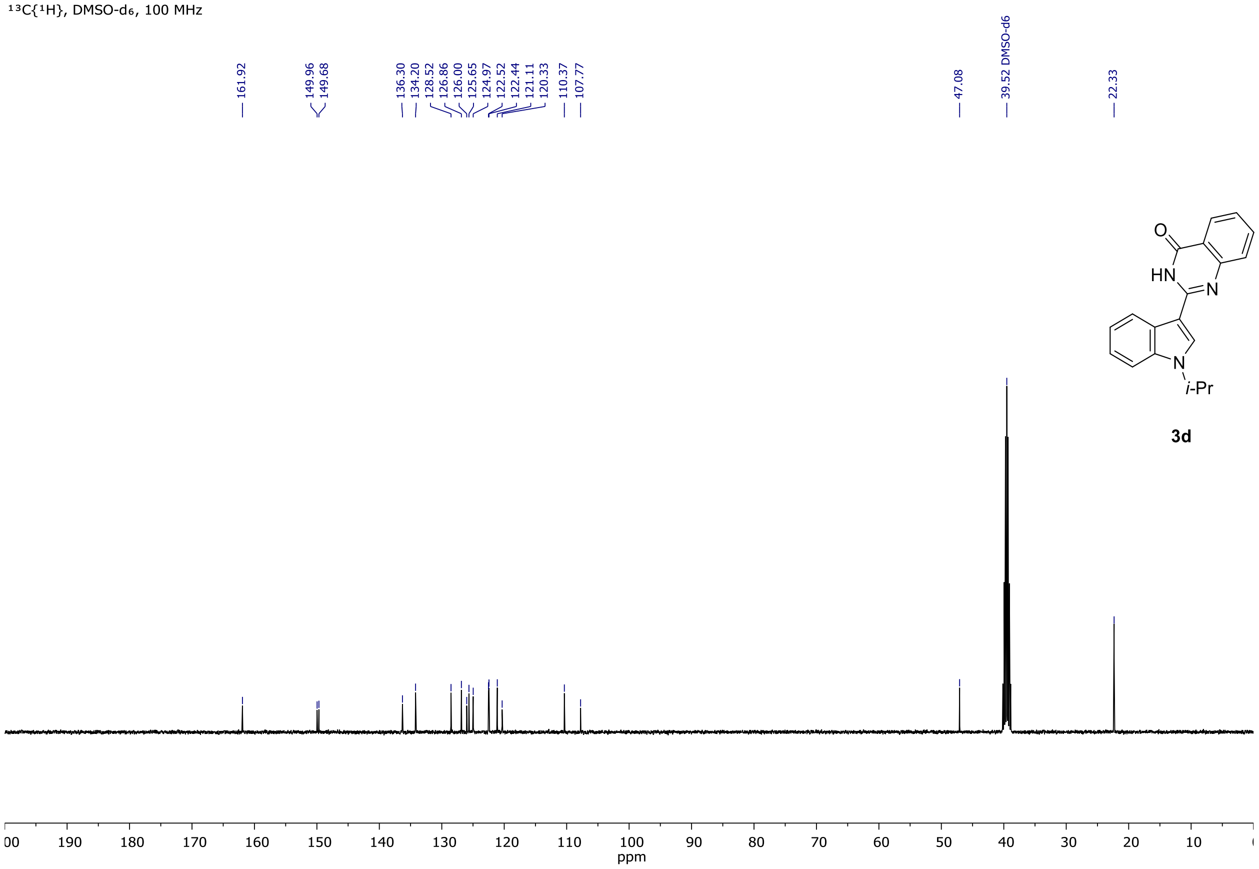

^1H, DMSO-d_6, 400 MHz

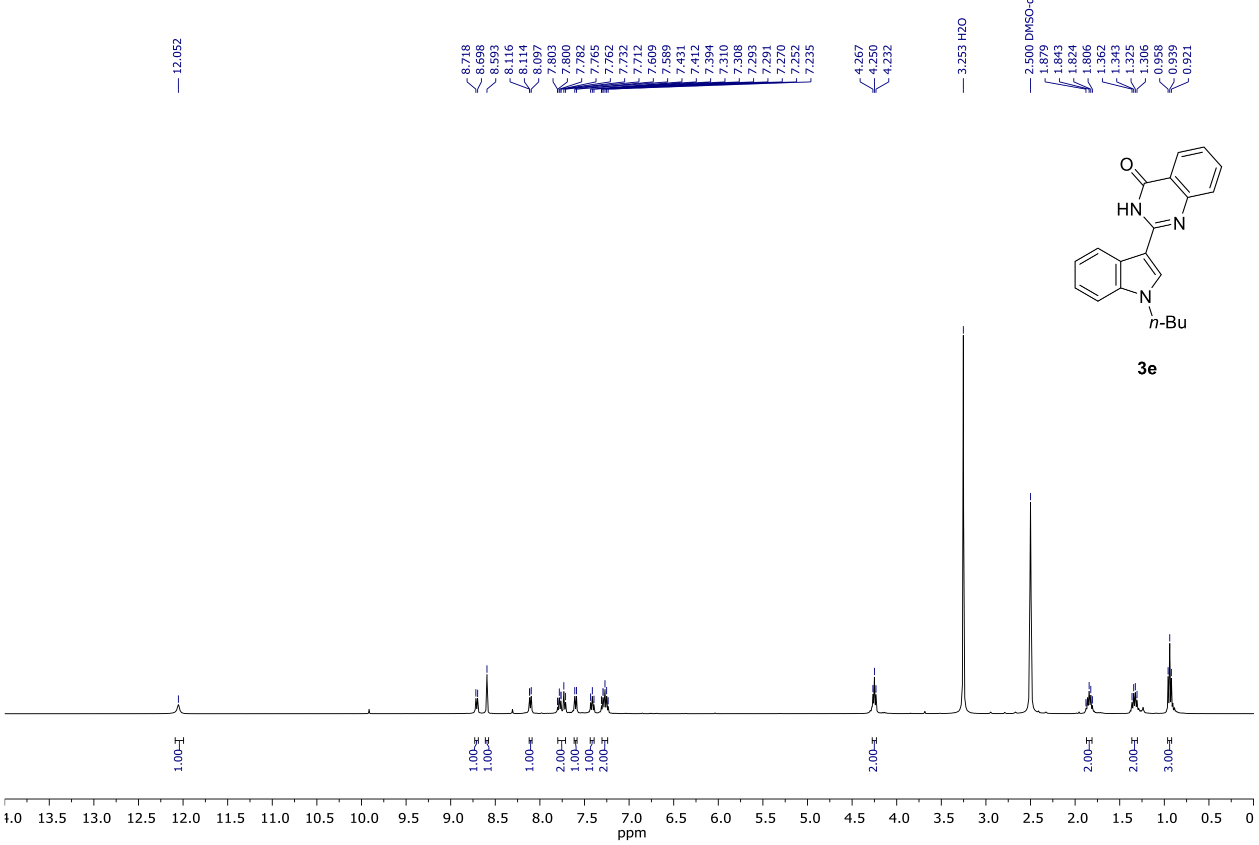

$^{13}C\{^1H\}$, DMSO-d_6, 100 MHz

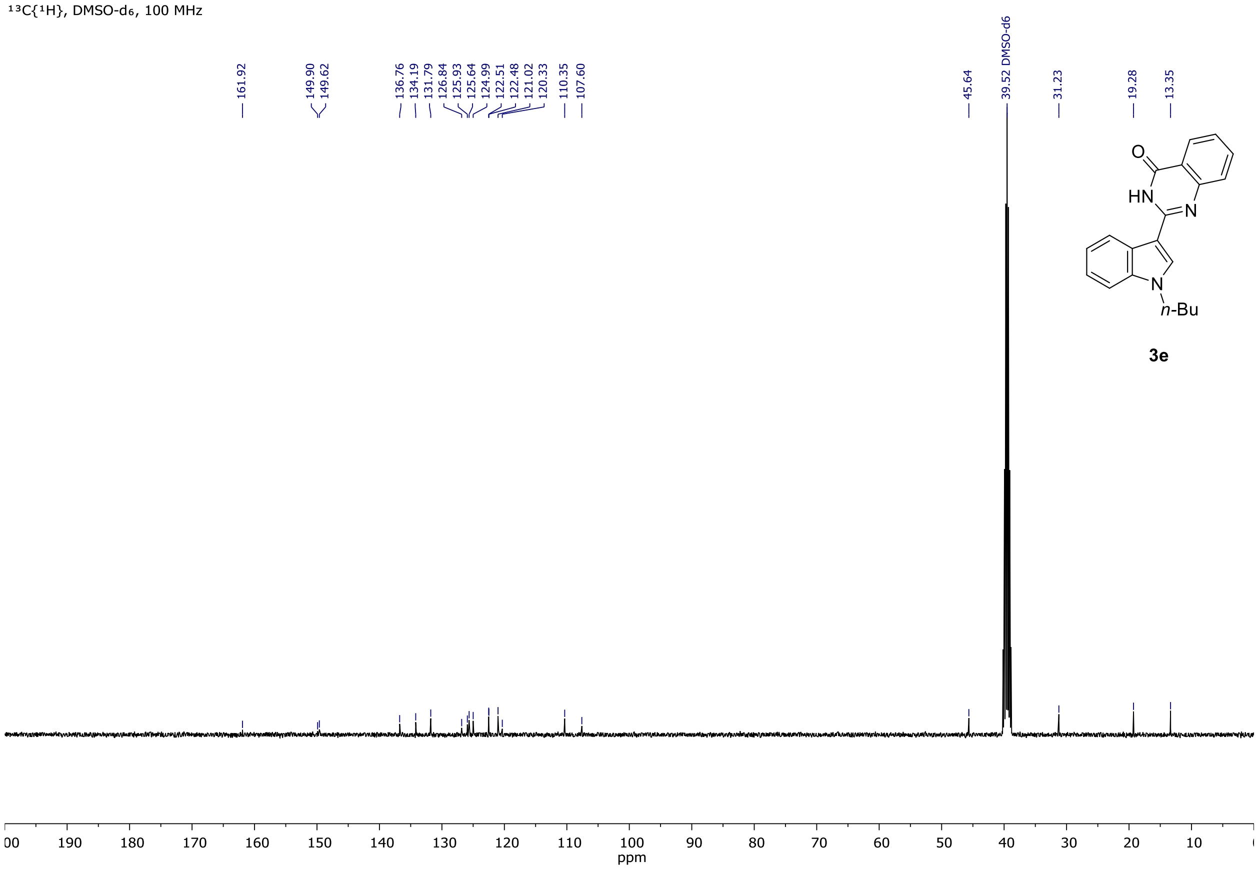

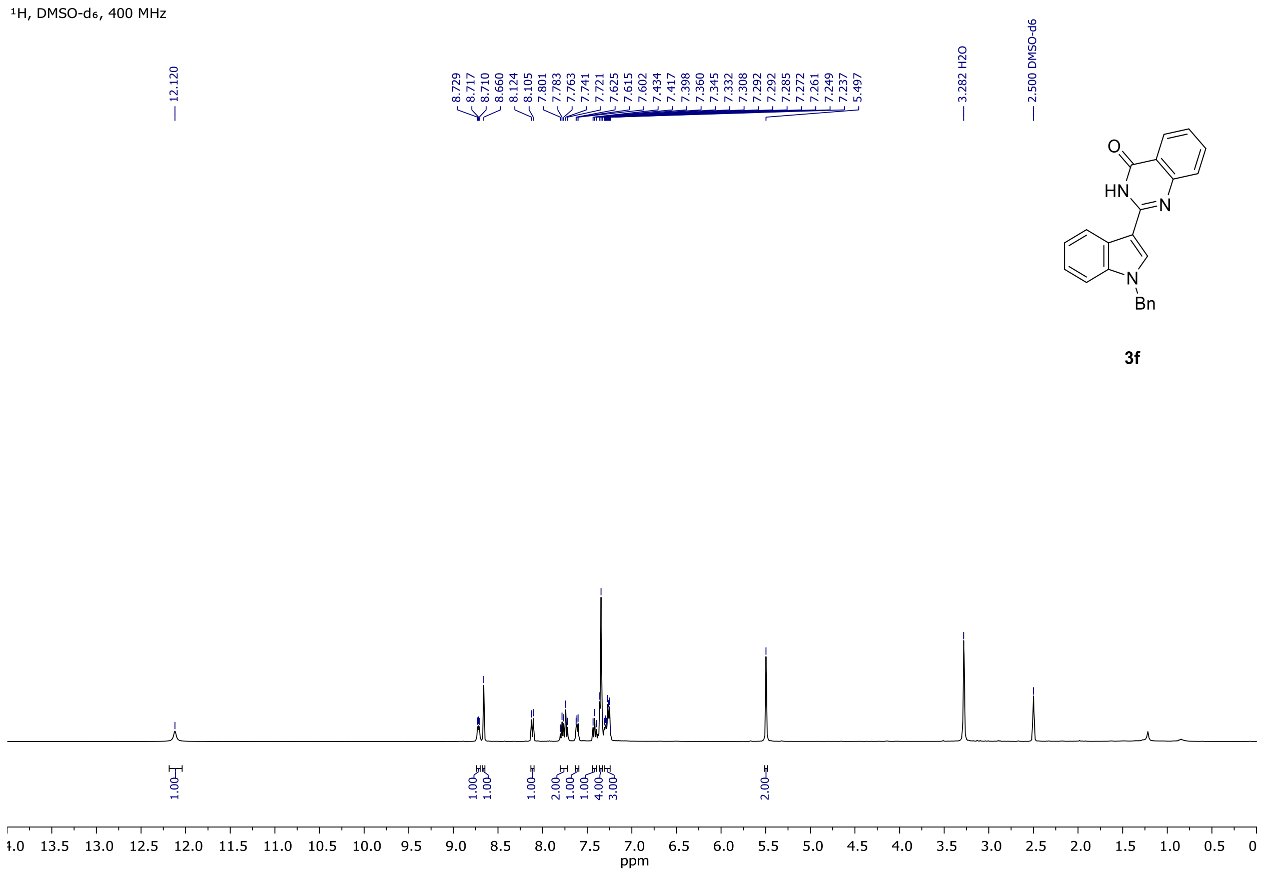

¹H, DMSO-d₆, 400 MHz
12.120
8.729
8.717
8.710
8.660
8.124
8.105
7.801
7.783
7.763
7.741
7.721
7.625
7.615
7.602
7.434
7.417
7.398
7.360
7.345
7.332
7.308
7.292
7.292
7.285
7.272
7.261
7.249
7.237
5.497
3.282 H2O
2.500 DMSO-d6
O
HN
N
N
Bn
3f
1.00
1.00
1.00
1.00
2.00
1.00
1.00
4.00
3.00
2.00
ppm

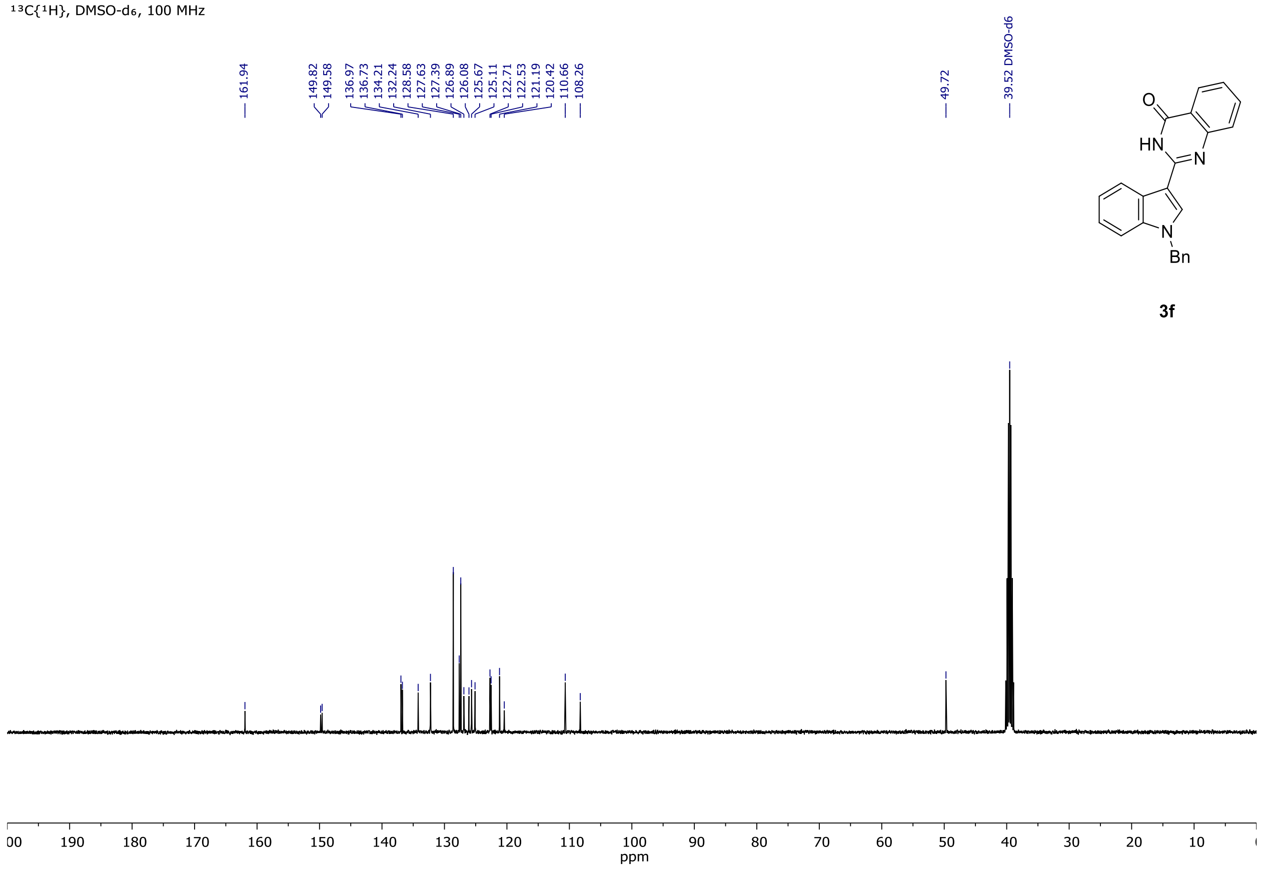

¹³C{¹H}, DMSO-d₆, 100 MHz
161.94
149.82
149.58
136.97
136.73
134.21
132.24
128.58
127.63
127.39
126.89
126.08
125.67
125.11
122.71
122.53
121.19
120.42
110.66
108.26
49.72
39.52 DMSO-d6
O
HN
N
N
Bn
3f
ppm

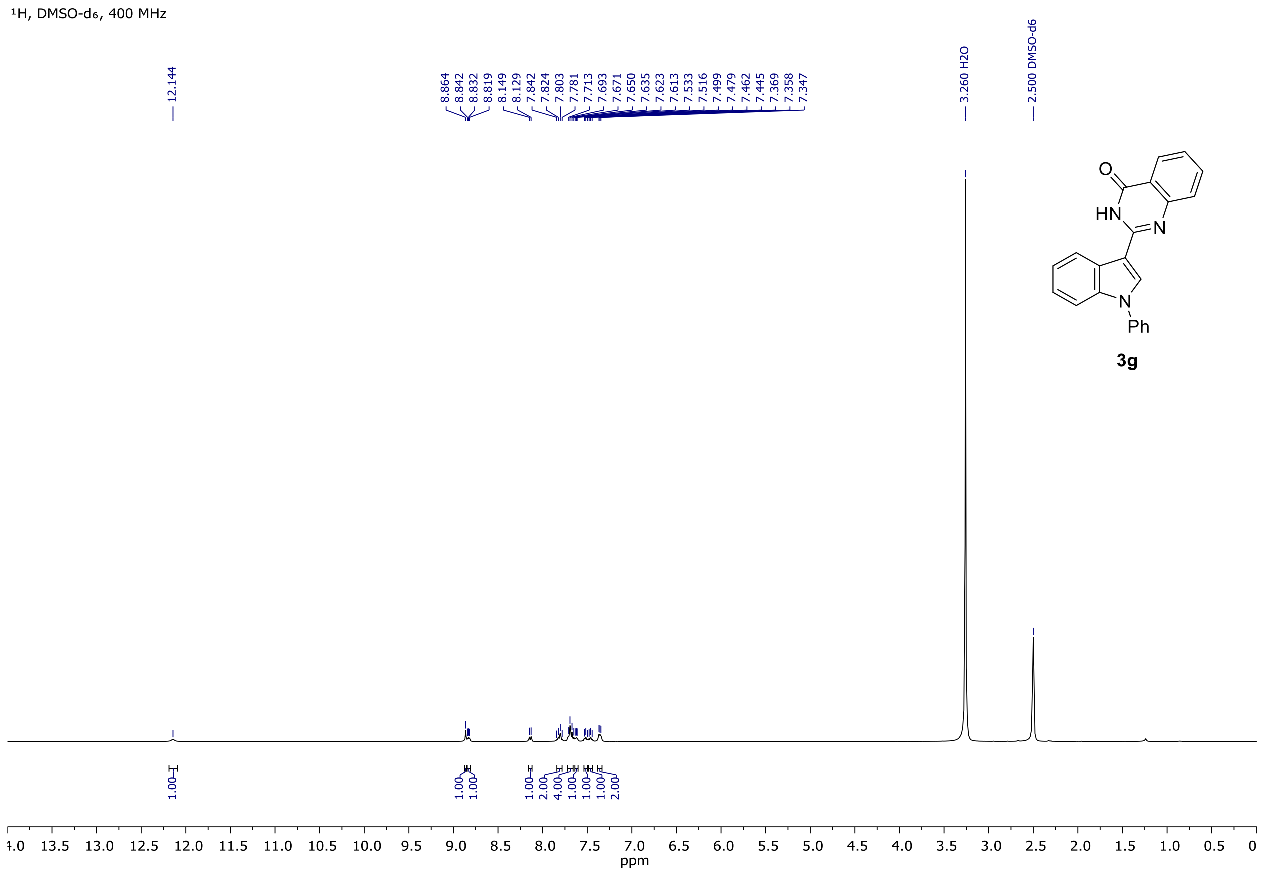
¹H, DMSO-d₆, 400 MHz
12.144
8.864
8.842
8.832
8.819
8.149
8.129
7.842
7.824
7.803
7.781
7.713
7.693
7.671
7.650
7.635
7.623
7.613
7.533
7.516
7.499
7.479
7.462
7.445
7.369
7.358
7.347
3.260 H2O
2.500 DMSO-d6
1.00
1.00
1.00
1.00
2.00
4.00
1.00
1.00
1.00
2.00
ppm
HN
N
O
N
Ph
3g

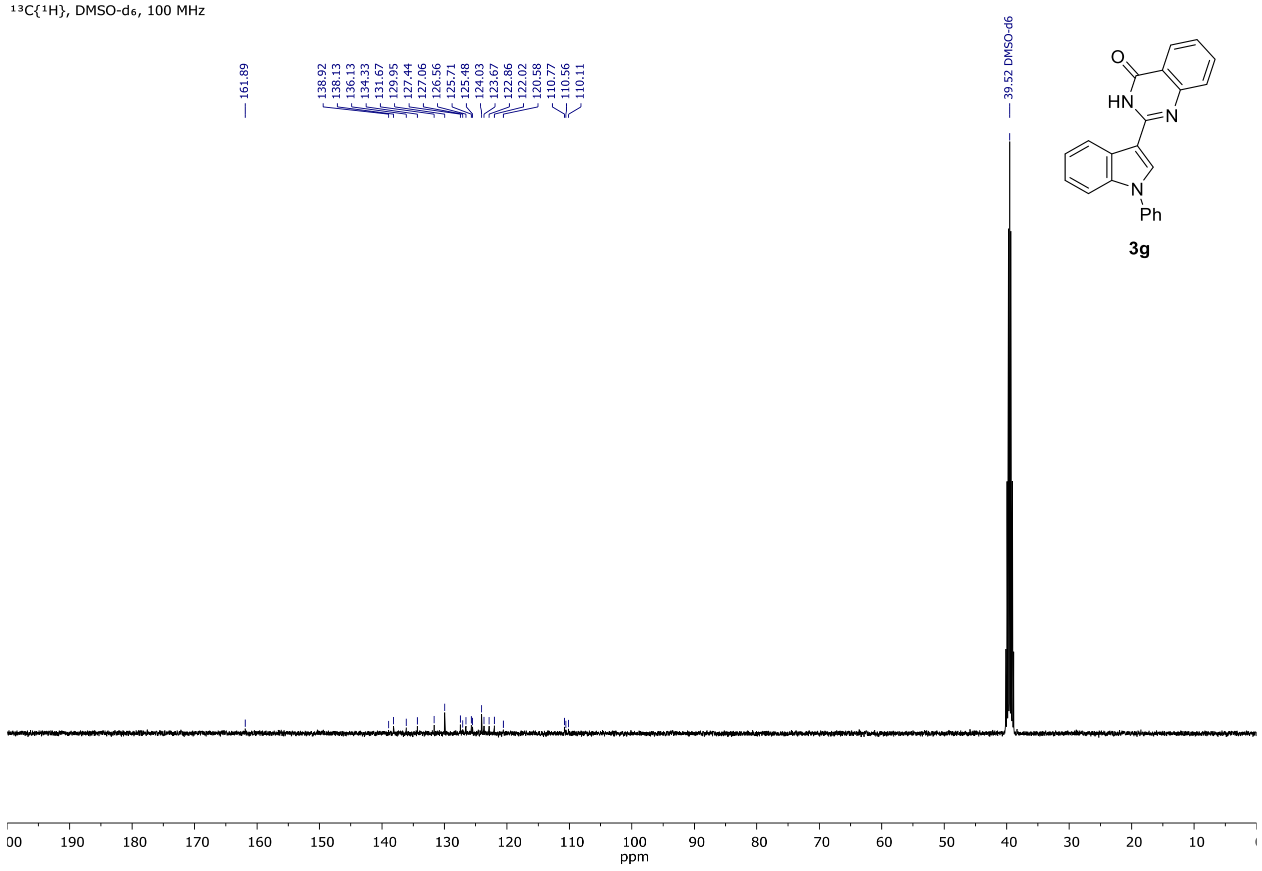
¹³C{¹H}, DMSO-d₆, 100 MHz
161.89
138.92
138.13
136.13
134.33
131.67
129.95
127.44
127.06
126.56
125.71
125.48
124.03
123.67
122.86
122.02
120.58
110.77
110.56
110.11
39.52 DMSO-d6
ppm
HN
N
O
N
Ph
3g

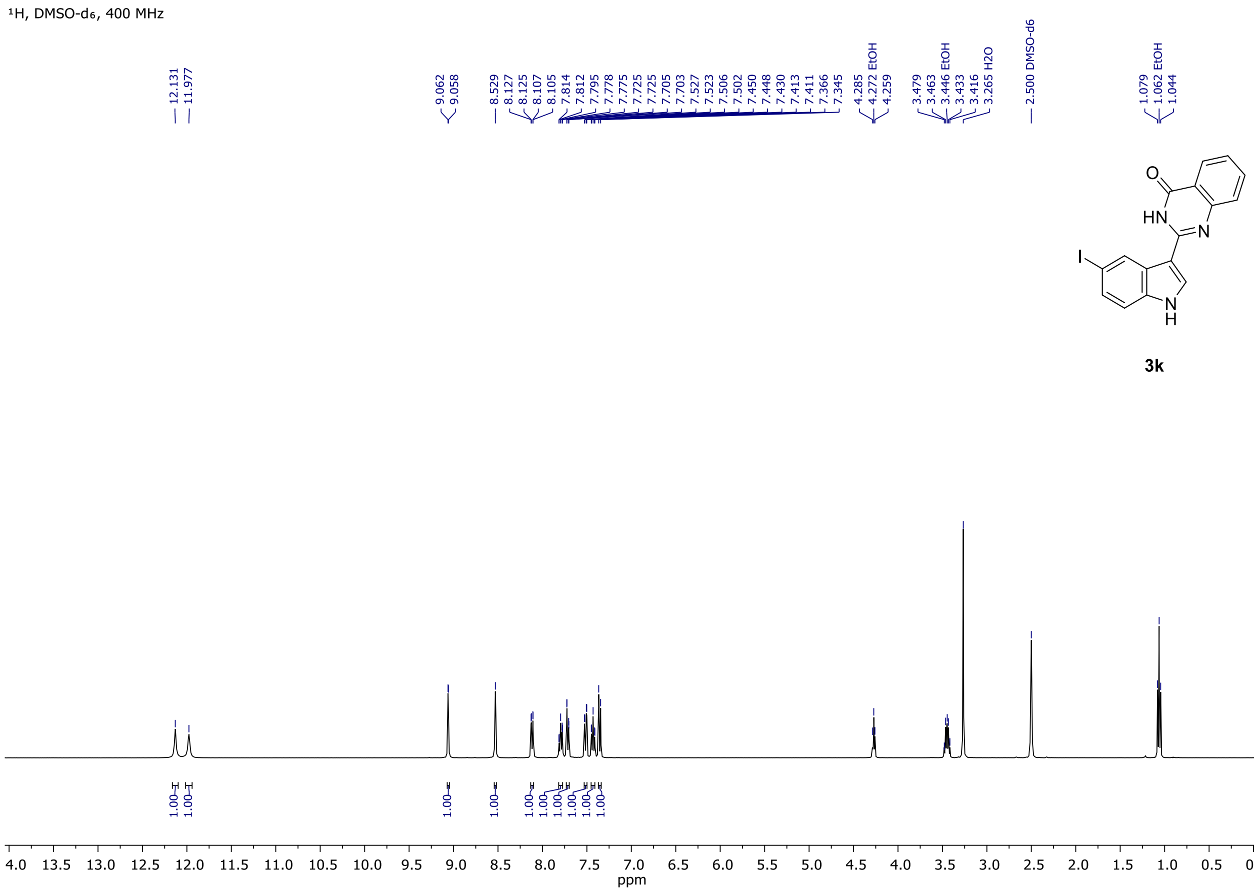

¹H, DMSO-d₆, 400 MHz
12.131
11.977
9.062
9.058
8.529
8.127
8.125
8.107
8.105
7.814
7.812
7.795
7.778
7.775
7.725
7.725
7.705
7.703
7.527
7.523
7.506
7.502
7.450
7.448
7.430
7.413
7.411
7.366
7.345
4.285
4.272 EtOH
4.259
3.479
3.463
3.446 EtOH
3.433
3.416
3.265 H2O
2.500 DMSO-d6
1.079
1.062 EtOH
1.044
O
HN
N
I
N
H
3k
ppm

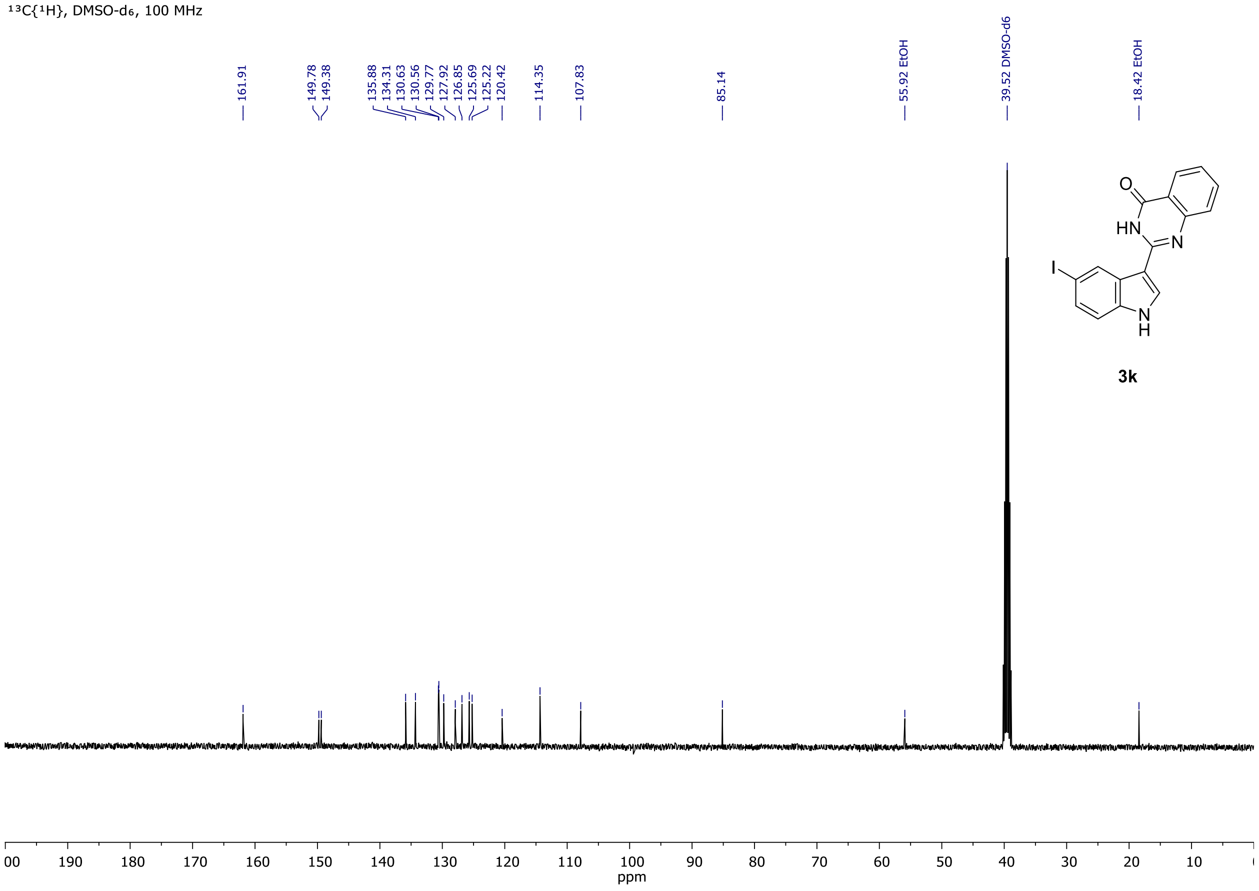

¹³C{¹H}, DMSO-d₆, 100 MHz
161.91
149.78
149.38
135.88
134.31
130.63
130.56
129.77
127.92
126.85
125.69
125.22
120.42
114.35
107.83
85.14
55.92 EtOH
39.52 DMSO-d6
18.42 EtOH
O
HN
N
I
N
H
3k
ppm

^1H, DMSO-d_6, 400 MHz

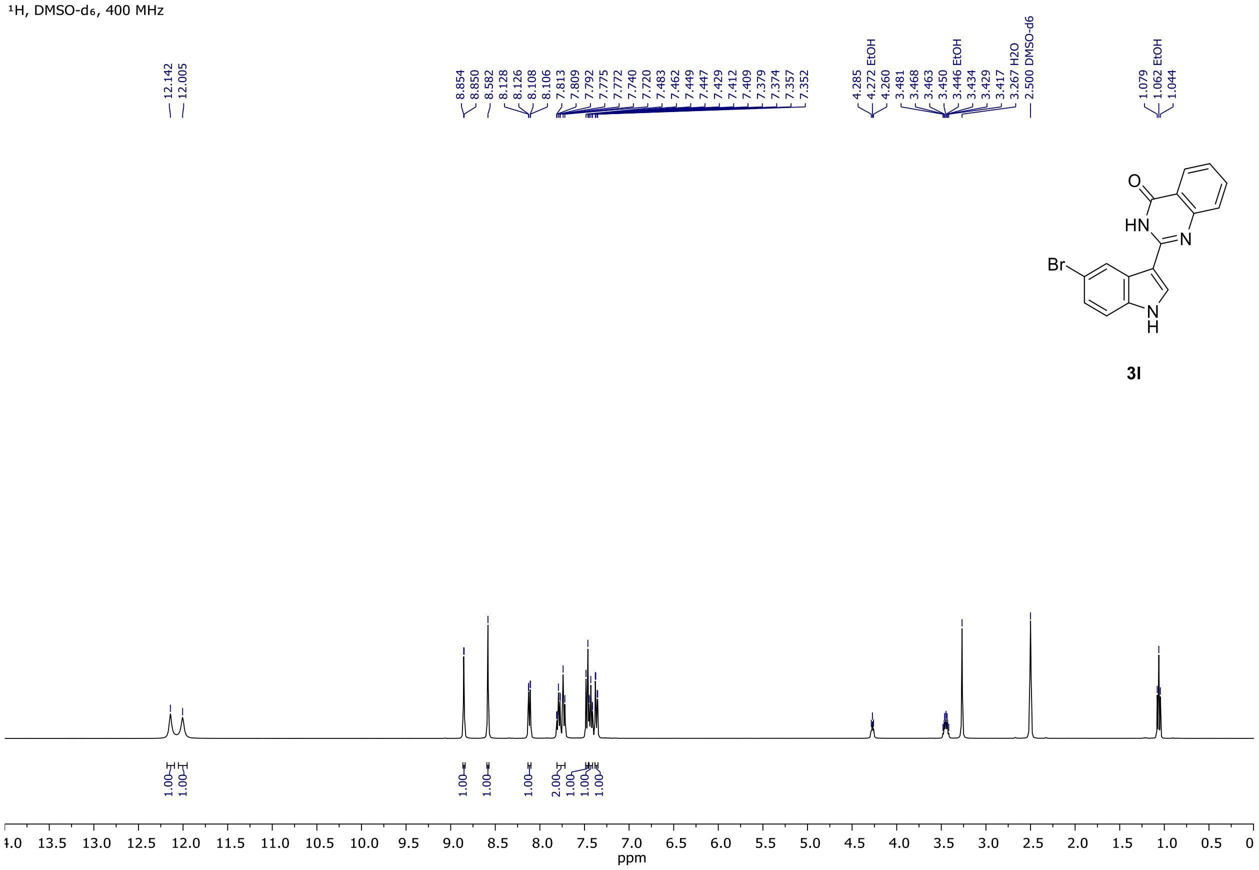

3l

$^{13}C\{^1H\}$, DMSO-d_6, 100 MHz

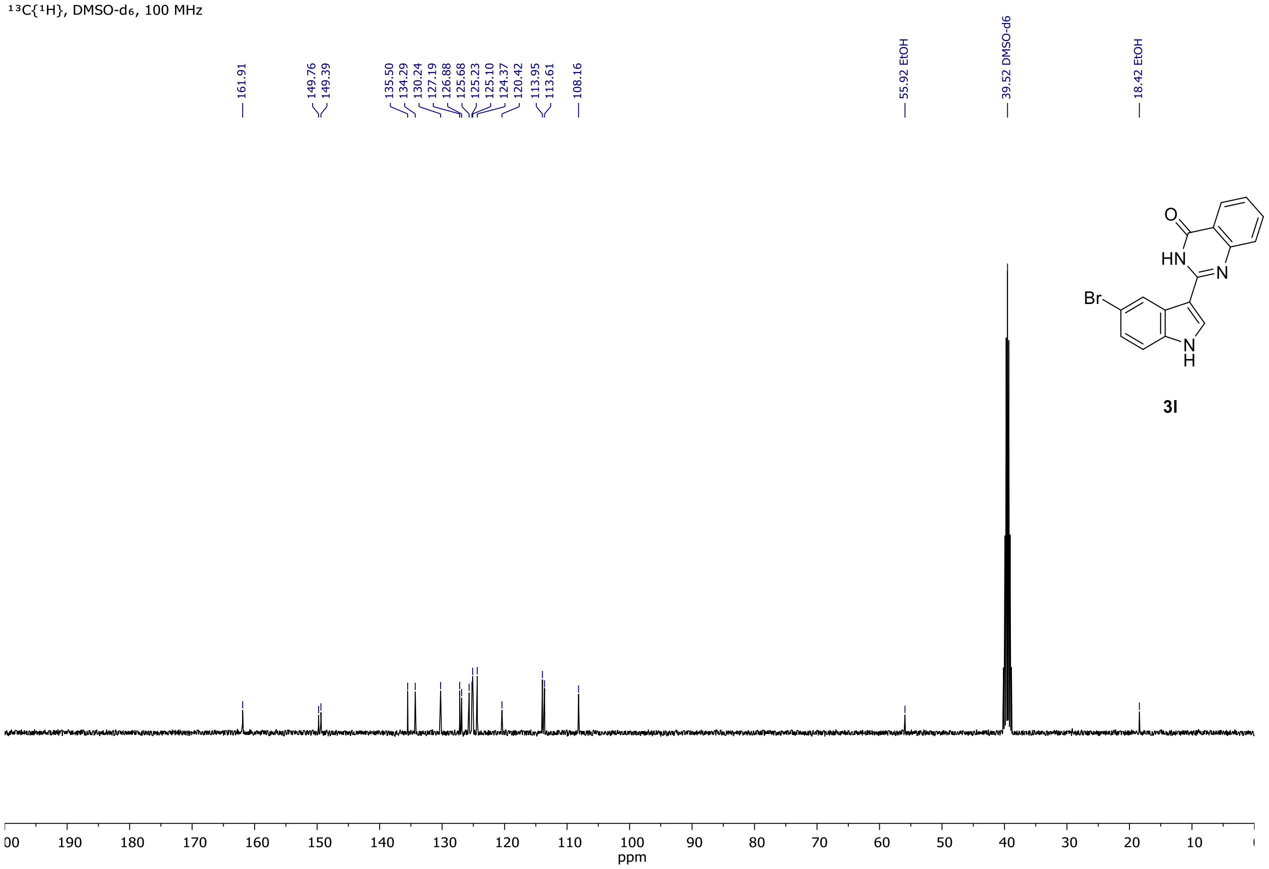

3l

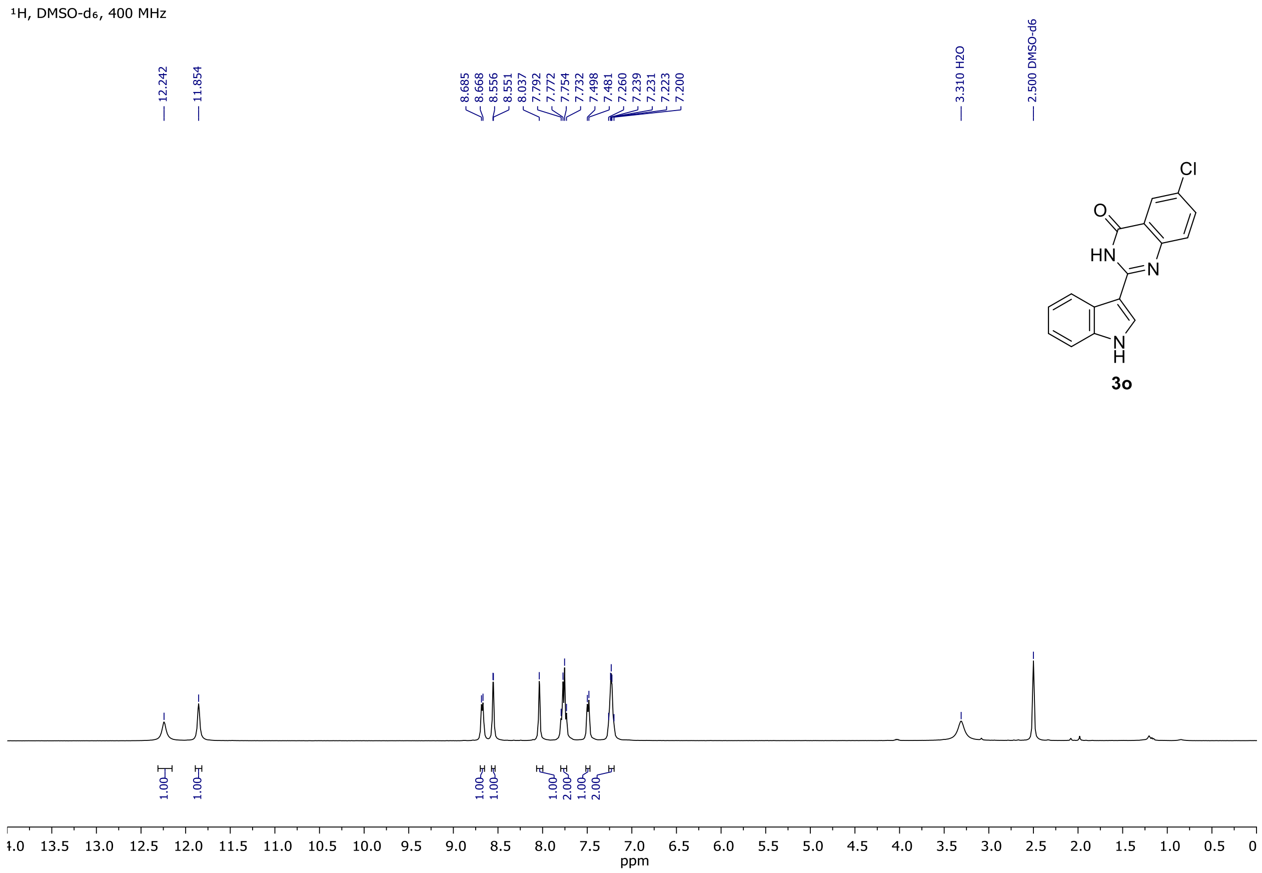
¹H, DMSO-d₆, 400 MHz
12.242
11.854
8.685
8.668
8.556
8.551
8.037
7.792
7.772
7.754
7.732
7.498
7.481
7.260
7.239
7.231
7.223
7.200
3.310 H2O
2.500 DMSO-d6
Cl
O
HN
N
N
H
3o
1.00
1.00
1.00
1.00
1.00
2.00
1.00
2.00
ppm

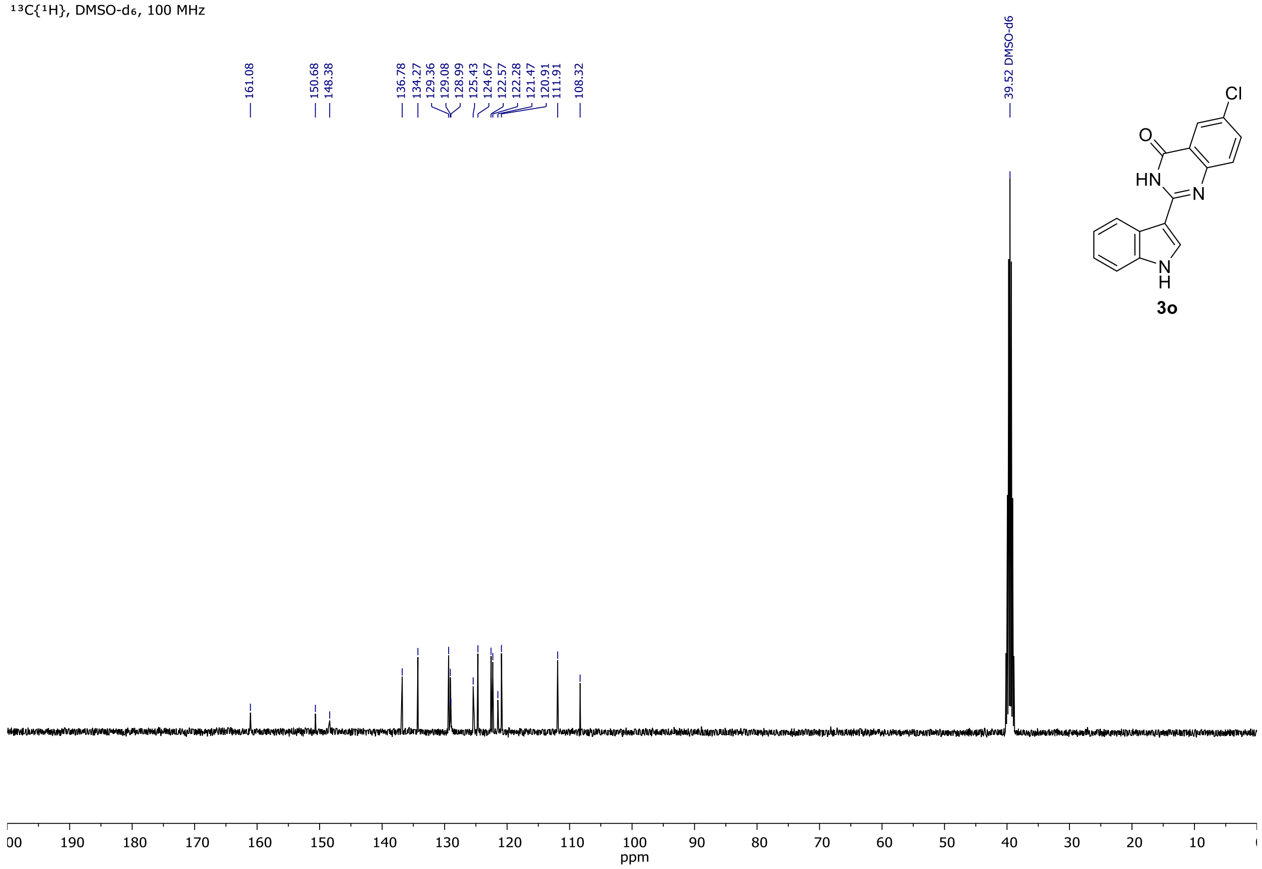
¹³C{¹H}, DMSO-d₆, 100 MHz
161.08
150.68
148.38
136.78
134.27
129.36
129.08
128.99
125.43
124.67
122.57
122.28
121.47
120.91
111.91
108.32
39.52 DMSO-d6
Cl
O
HN
N
N
H
3o
ppm

¹H, DMSO-d₆, 400 MHz

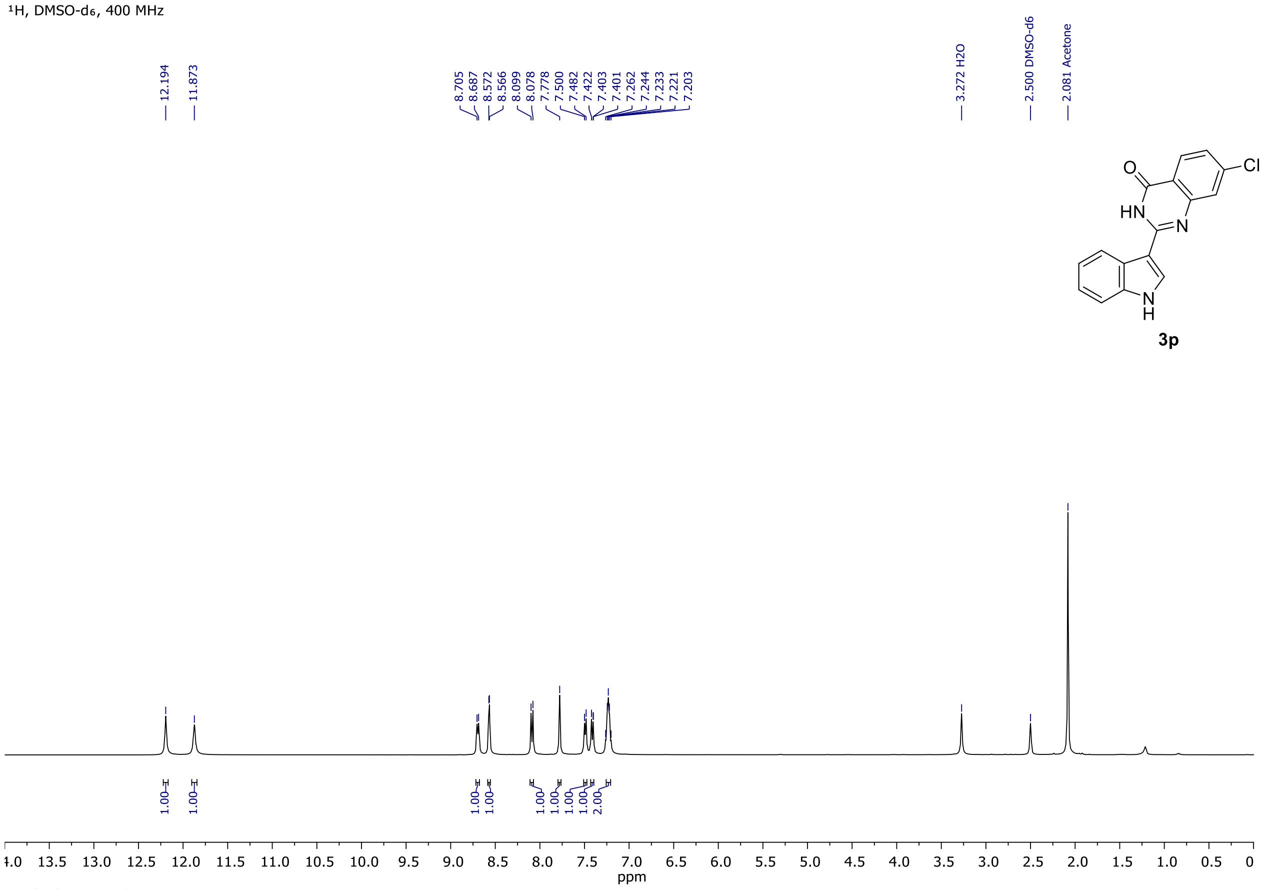

¹³C{¹H}, DMSO-d₆, 100 MHz

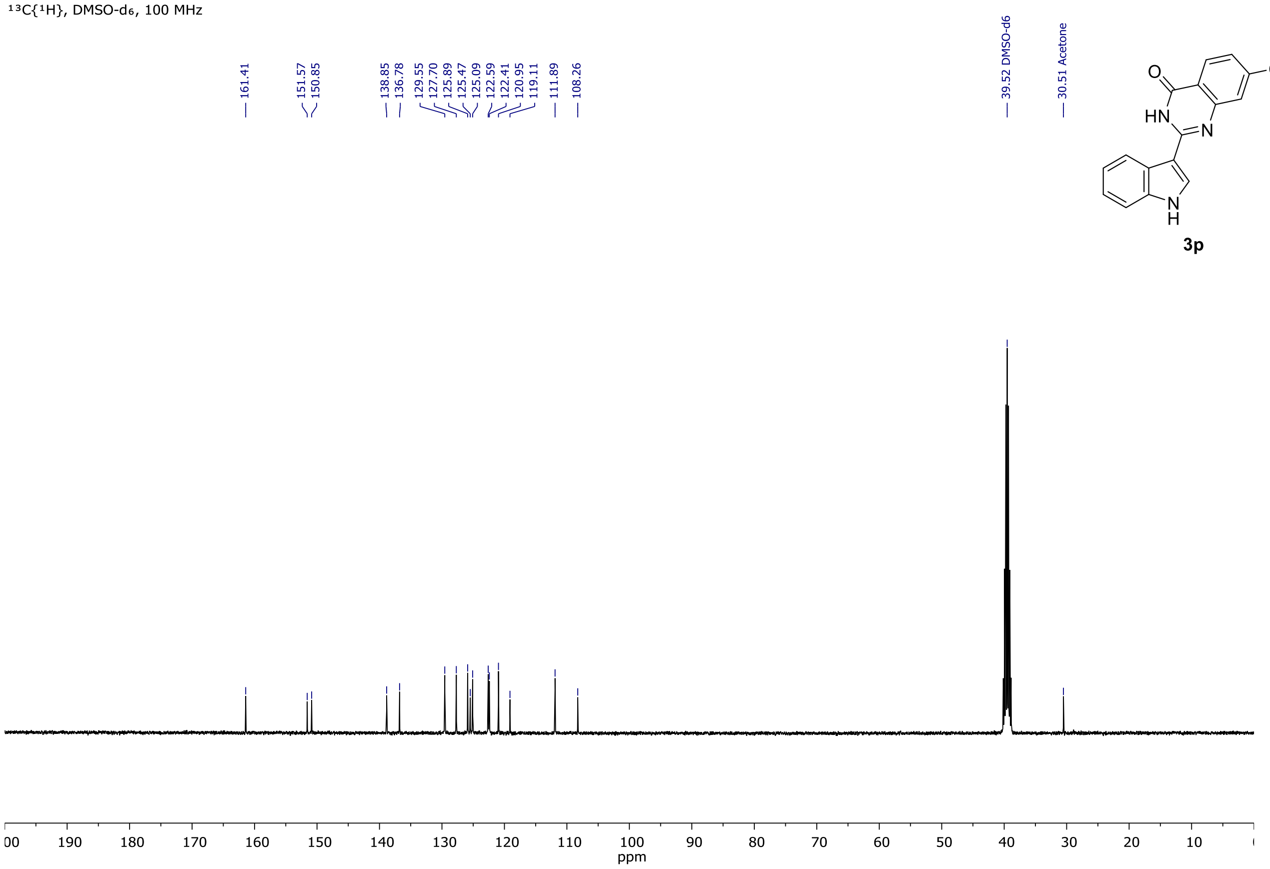

¹H, DMSO-d₆, 400 MHz

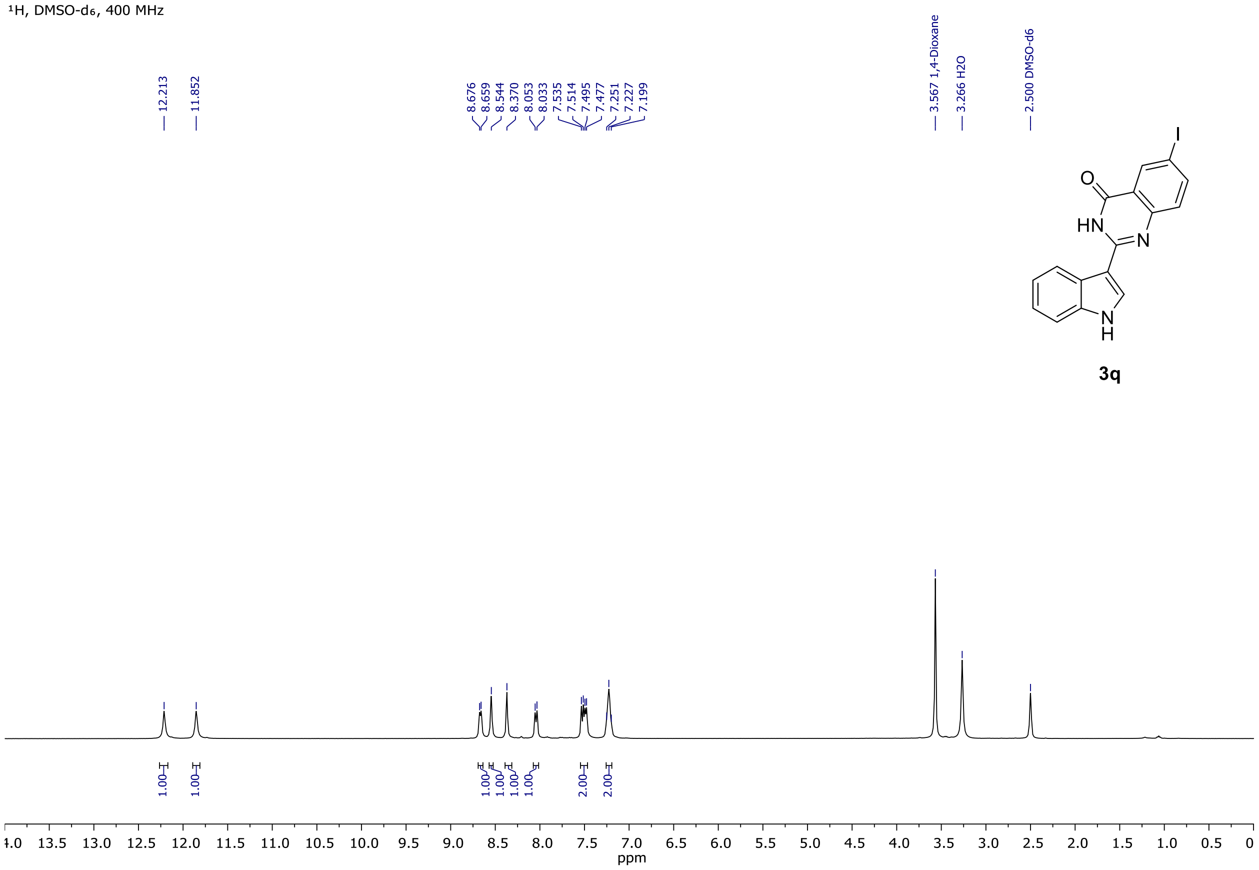

¹³C{¹H}, DMSO-d₆, 100 MHz

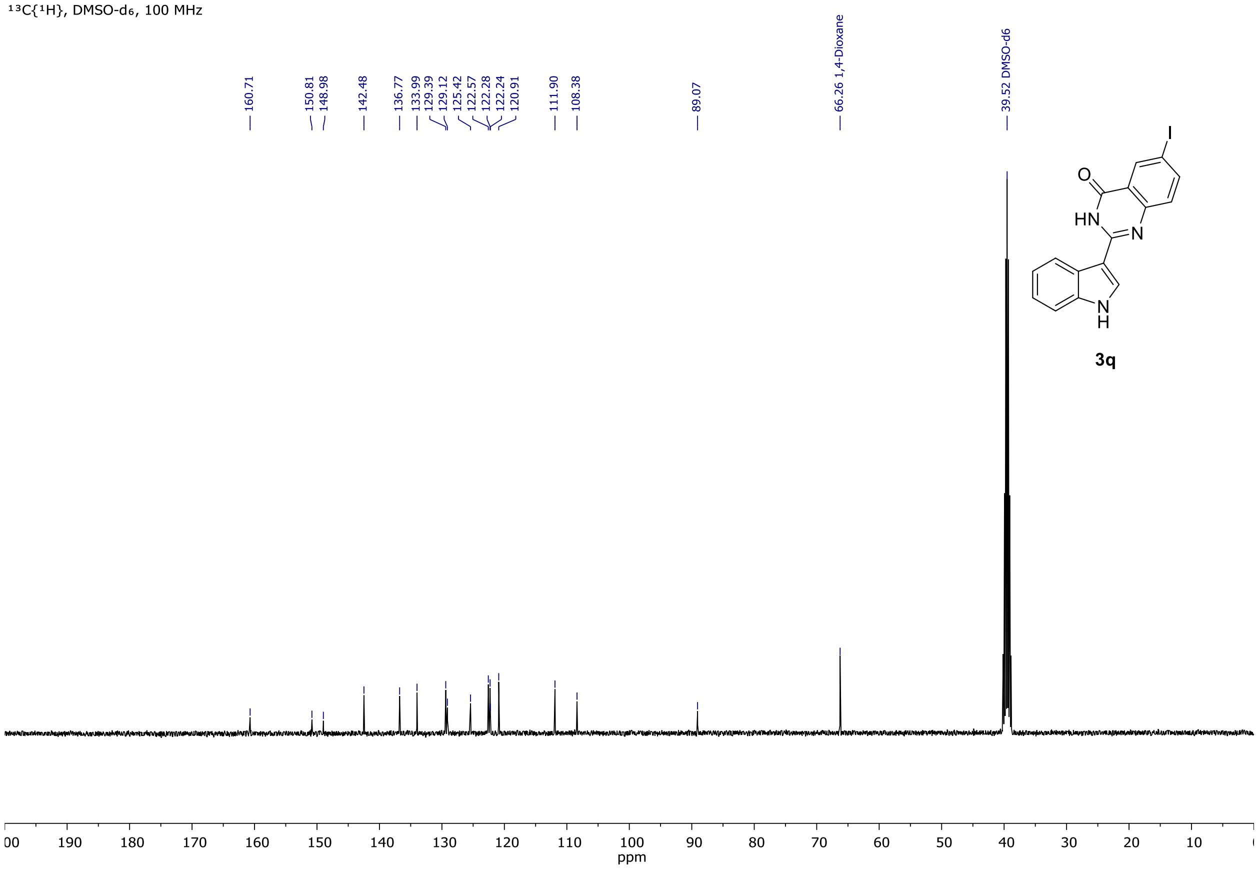

¹H, DMSO-d₆, 400 MHz

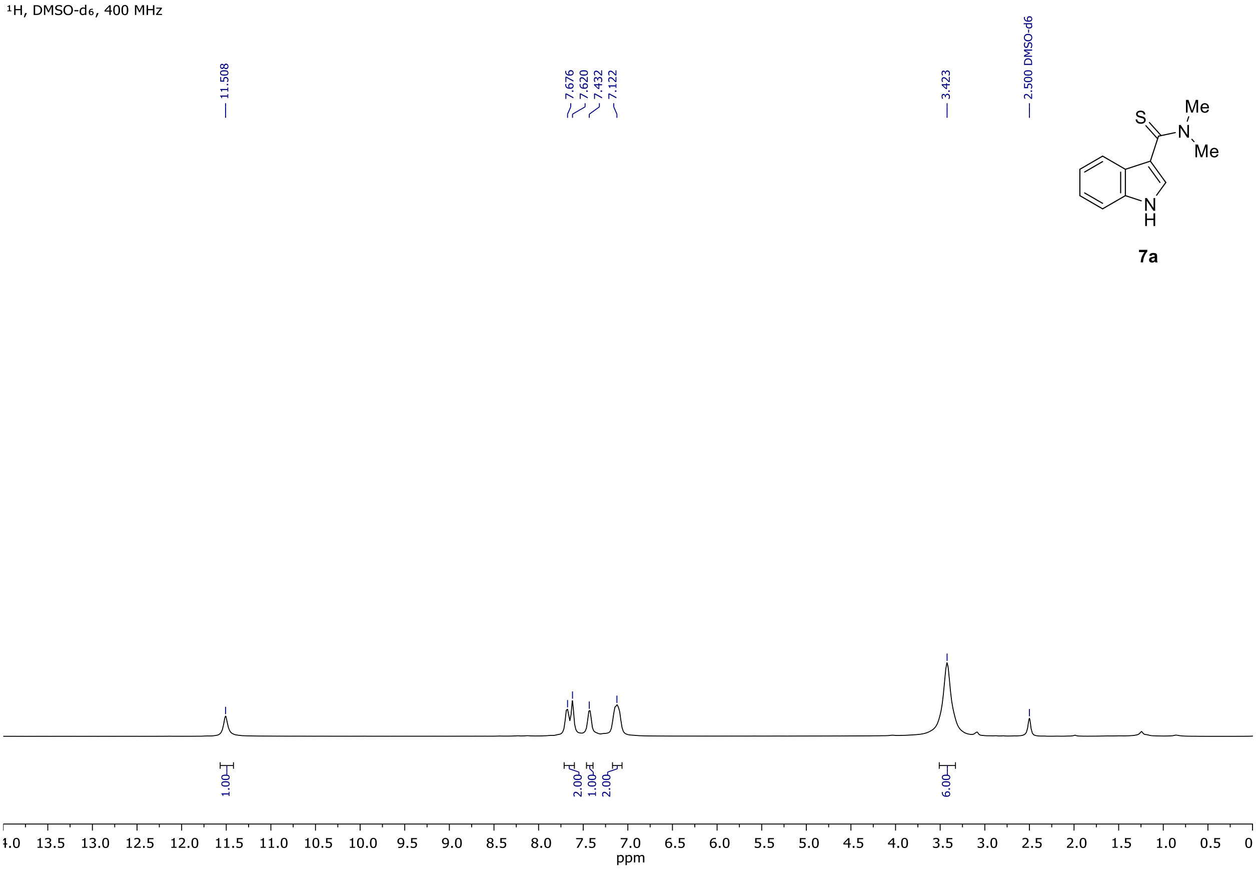

¹³C{¹H}, DMSO-d₆, 100 MHz

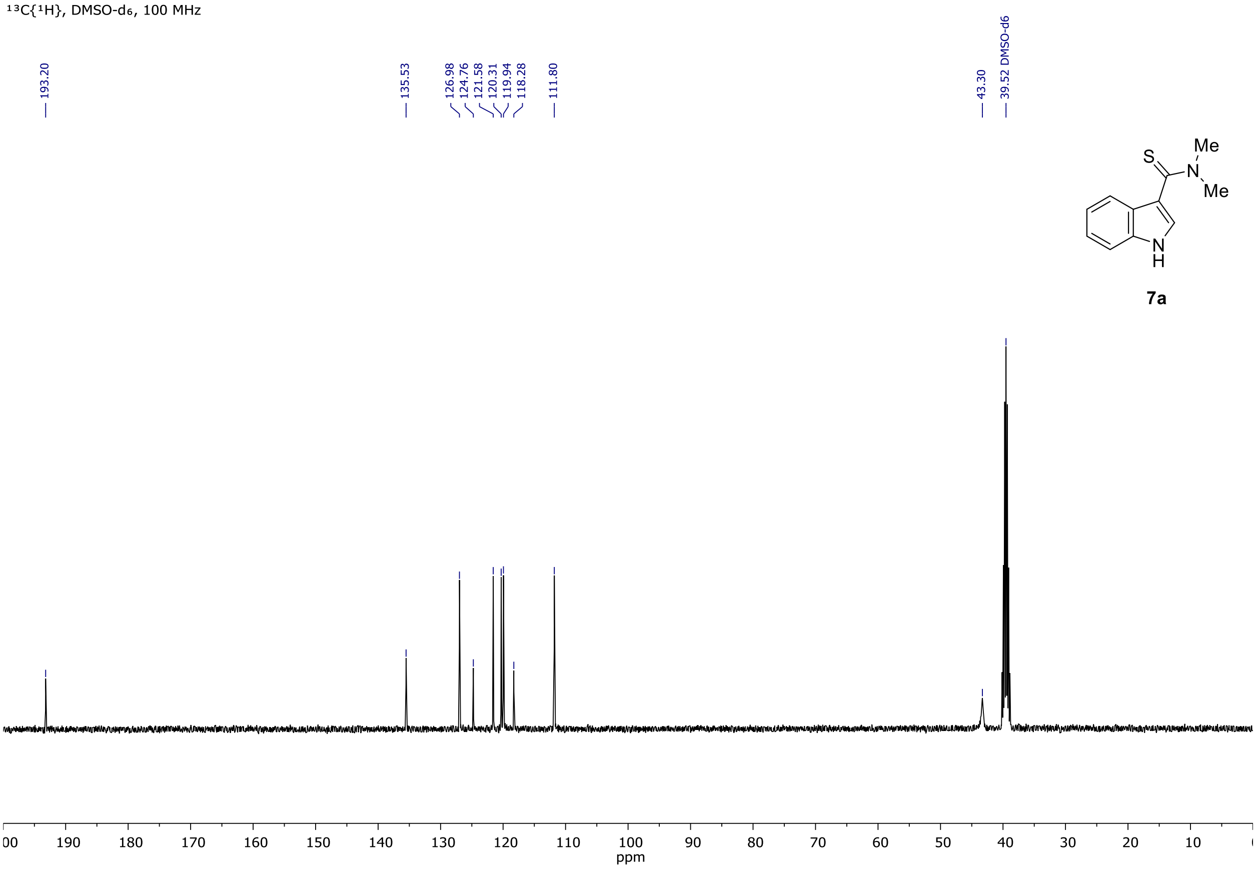

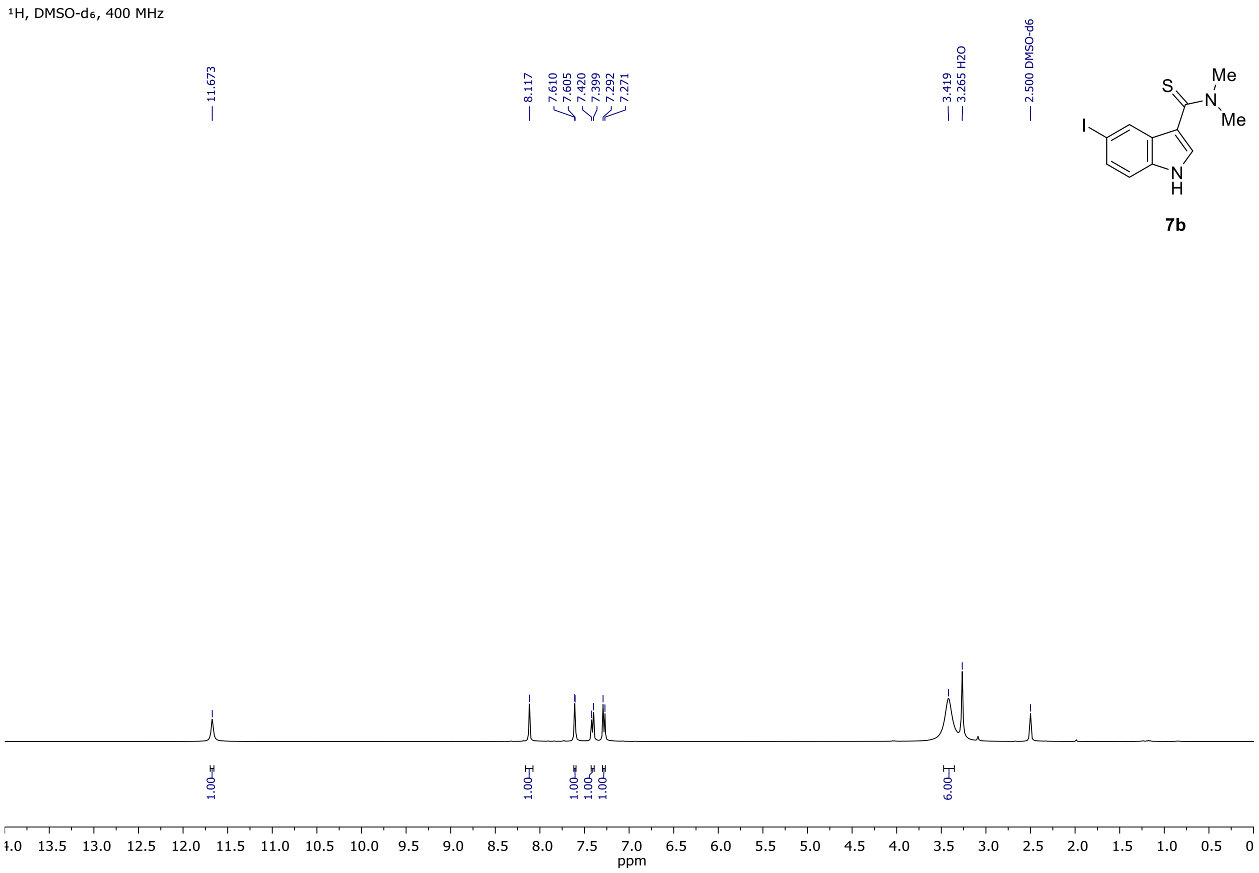
¹H, DMSO-d₆, 400 MHz
11.673
8.117
7.610
7.605
7.420
7.399
7.292
7.271
3.419
3.265 H2O
2.500 DMSO-d6
S
N
Me
Me
I
N
H
7b
1.00
1.00
1.00
1.00
1.00
6.00
ppm

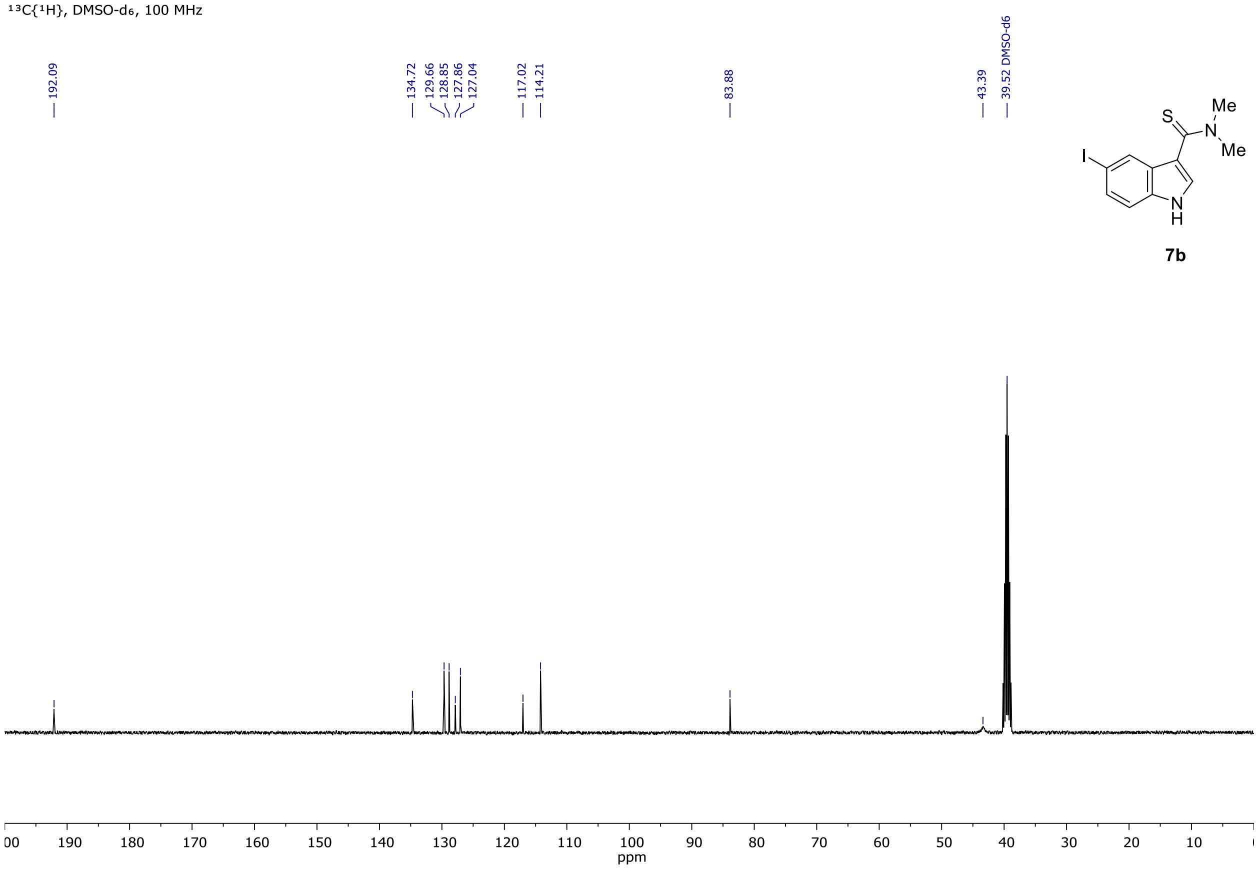
¹³C{¹H}, DMSO-d₆, 100 MHz
192.09
134.72
129.66
128.85
127.86
127.04
117.02
114.21
83.88
43.39
39.52 DMSO-d6
S
N
Me
Me
I
N
H
7b
ppm

^1H, DMSO-d_6, 400 MHz

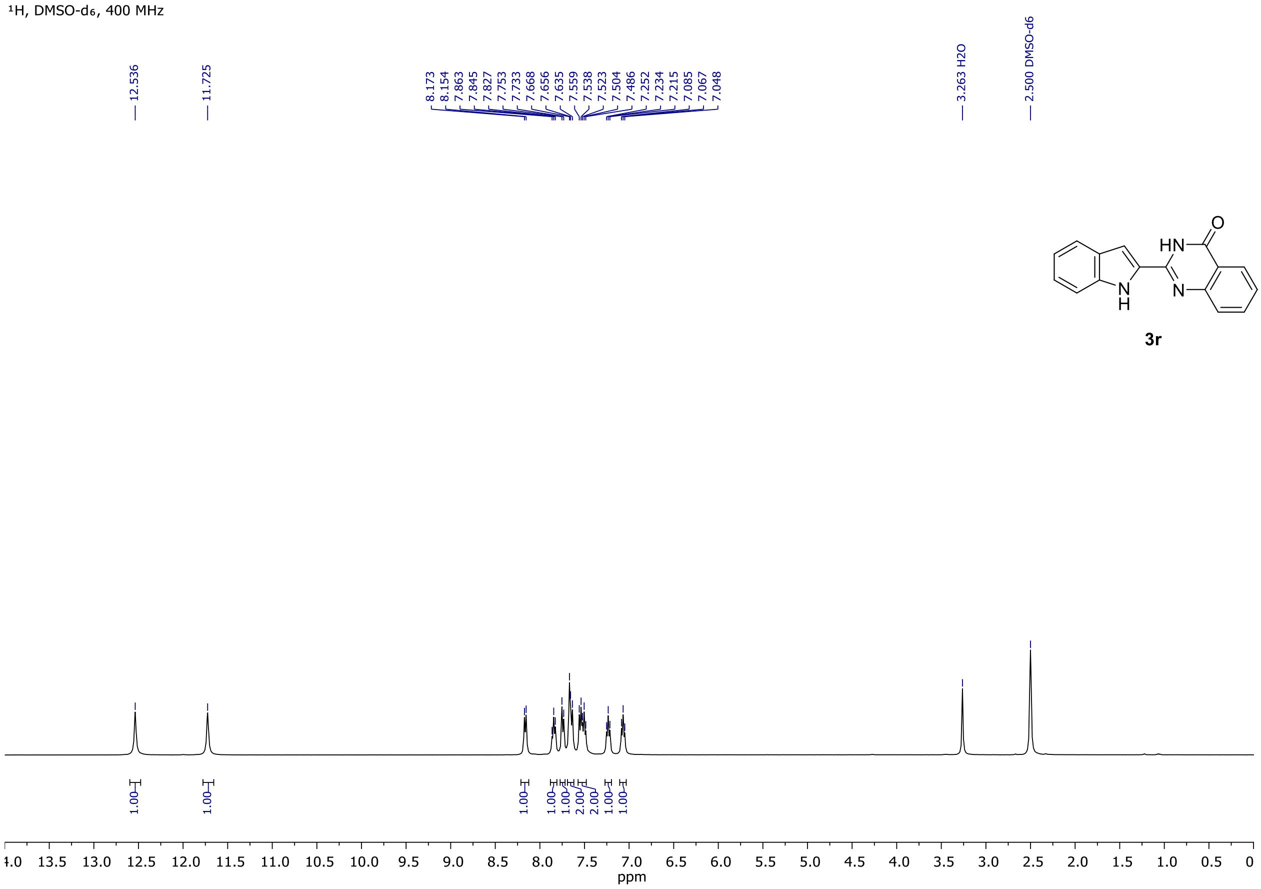

$^{13}C\{^1H\}$, DMSO-d_6, 100 MHz

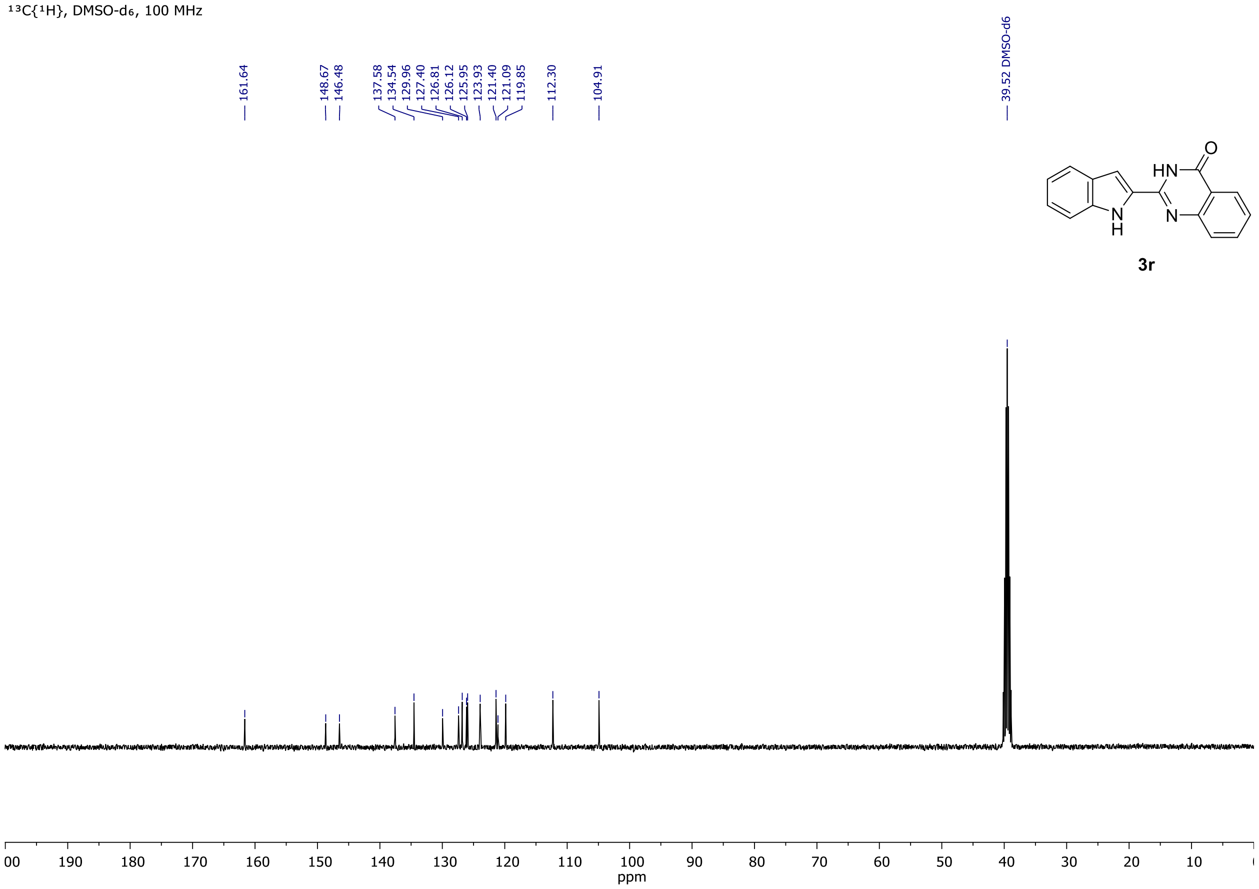

¹H, DMSO-d₆, 400 MHz

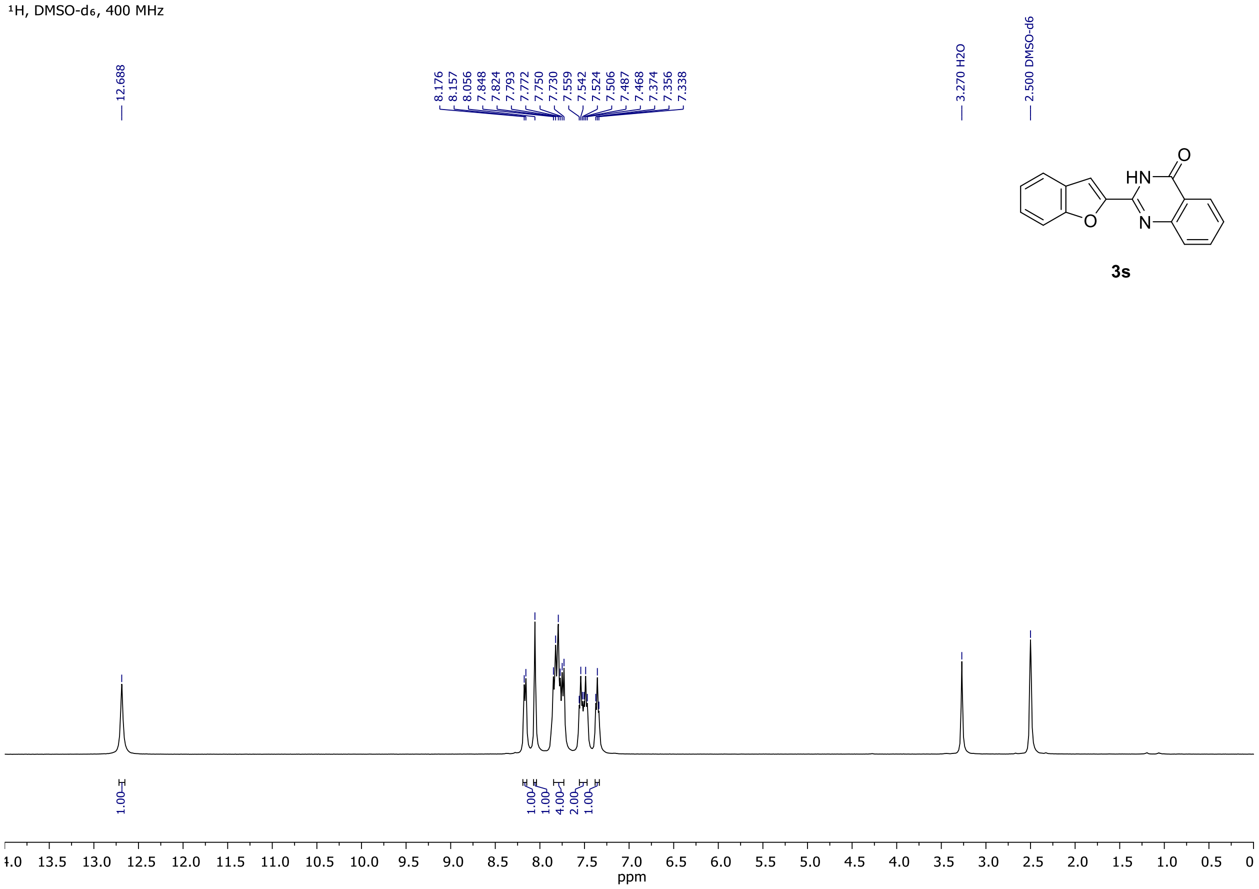

¹³C{¹H}, DMSO-d₆, 100 MHz

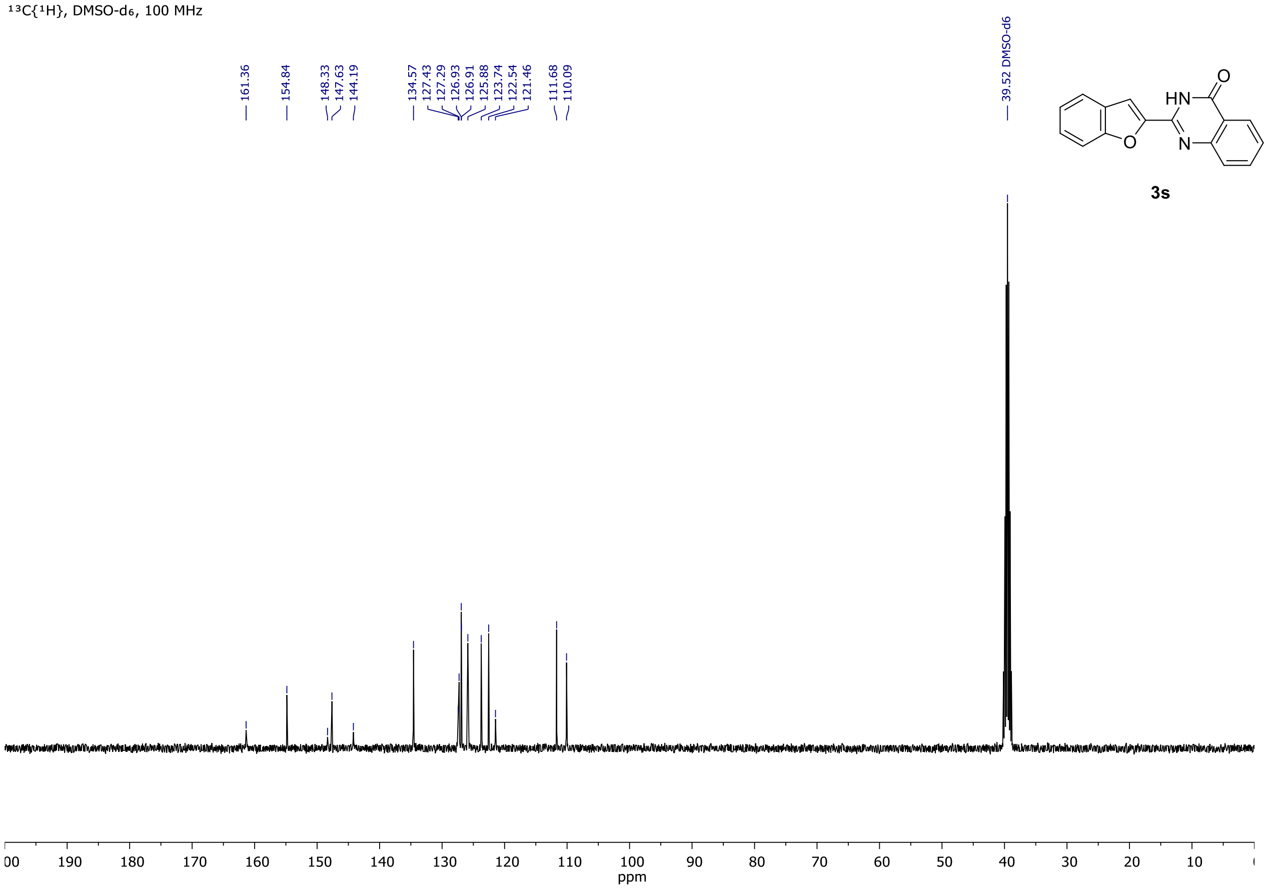

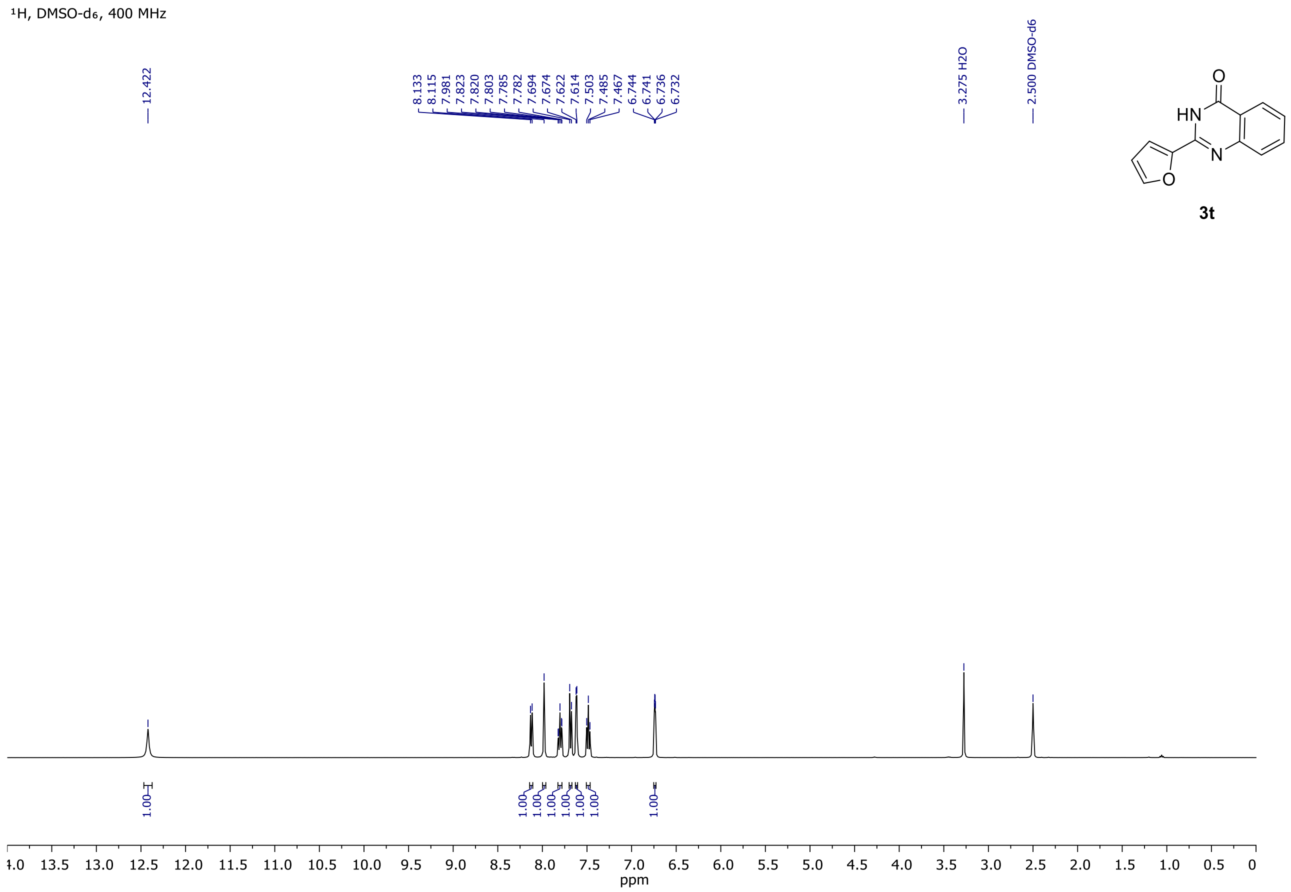
¹H, DMSO-d₆, 400 MHz
12.422
8.133
8.115
7.981
7.823
7.820
7.803
7.785
7.782
7.694
7.674
7.622
7.614
7.503
7.485
7.467
6.744
6.741
6.736
6.732
3.275 H2O
2.500 DMSO-d6
O
HN
N
O
3t
1.00
1.00
1.00
1.00
1.00
1.00
1.00
1.00
ppm

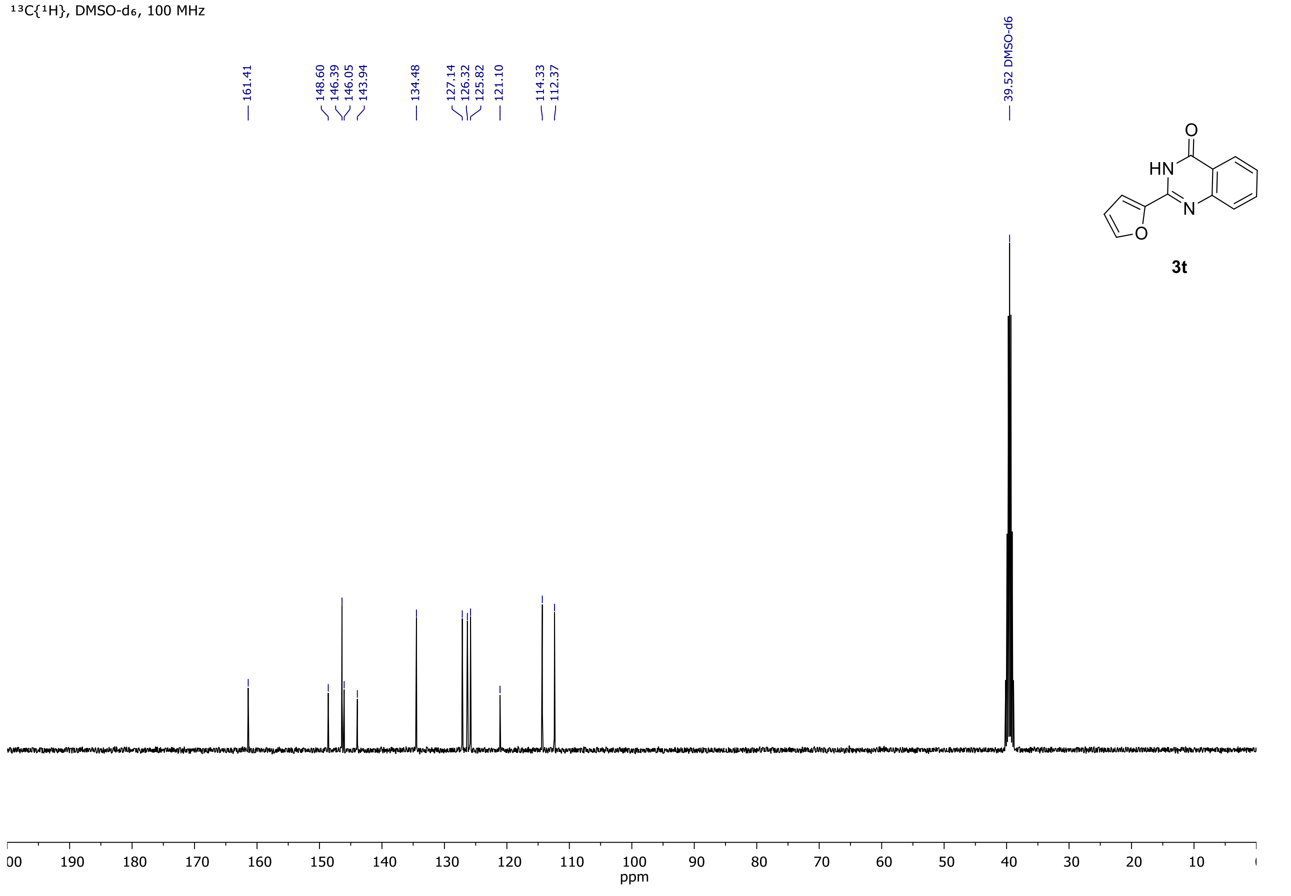
¹³C{¹H}, DMSO-d₆, 100 MHz
161.41
148.60
146.39
146.05
143.94
134.48
127.14
126.32
125.82
121.10
114.33
112.37
39.52 DMSO-d6
O
HN
N
O
3t
ppm

¹H, DMSO-d₆, 400 MHz

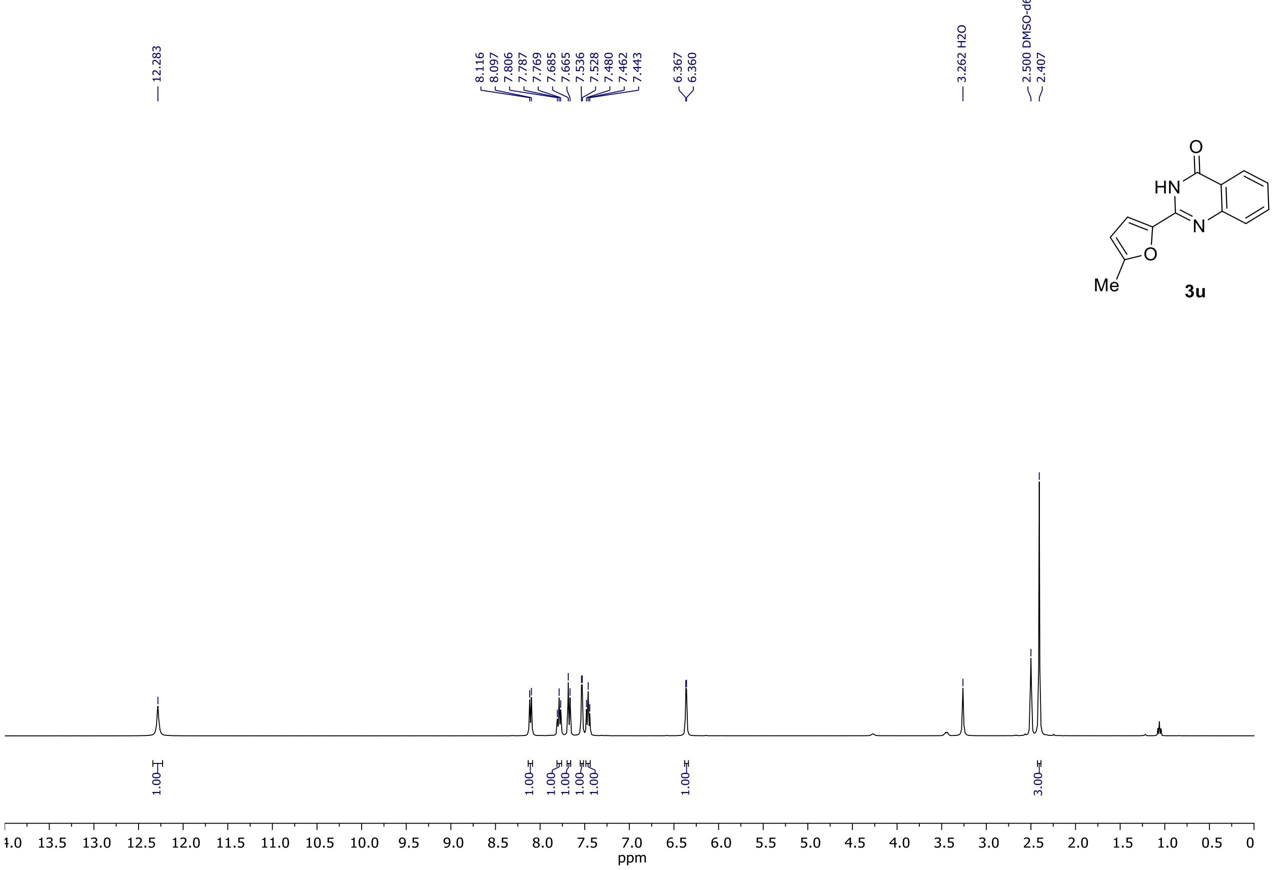

¹³C{¹H}, DMSO-d₆, 100 MHz

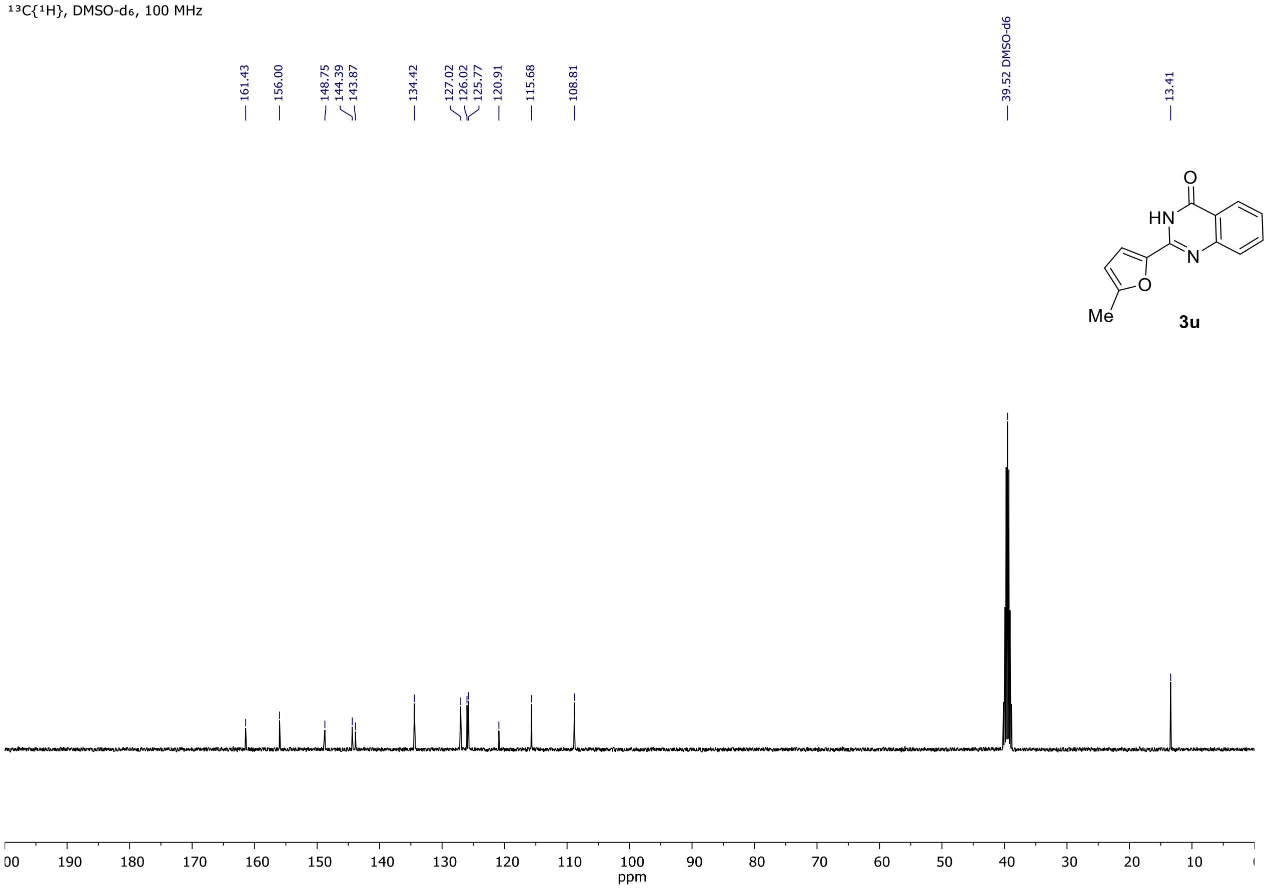

¹H, DMSO-d₆, 400 MHz

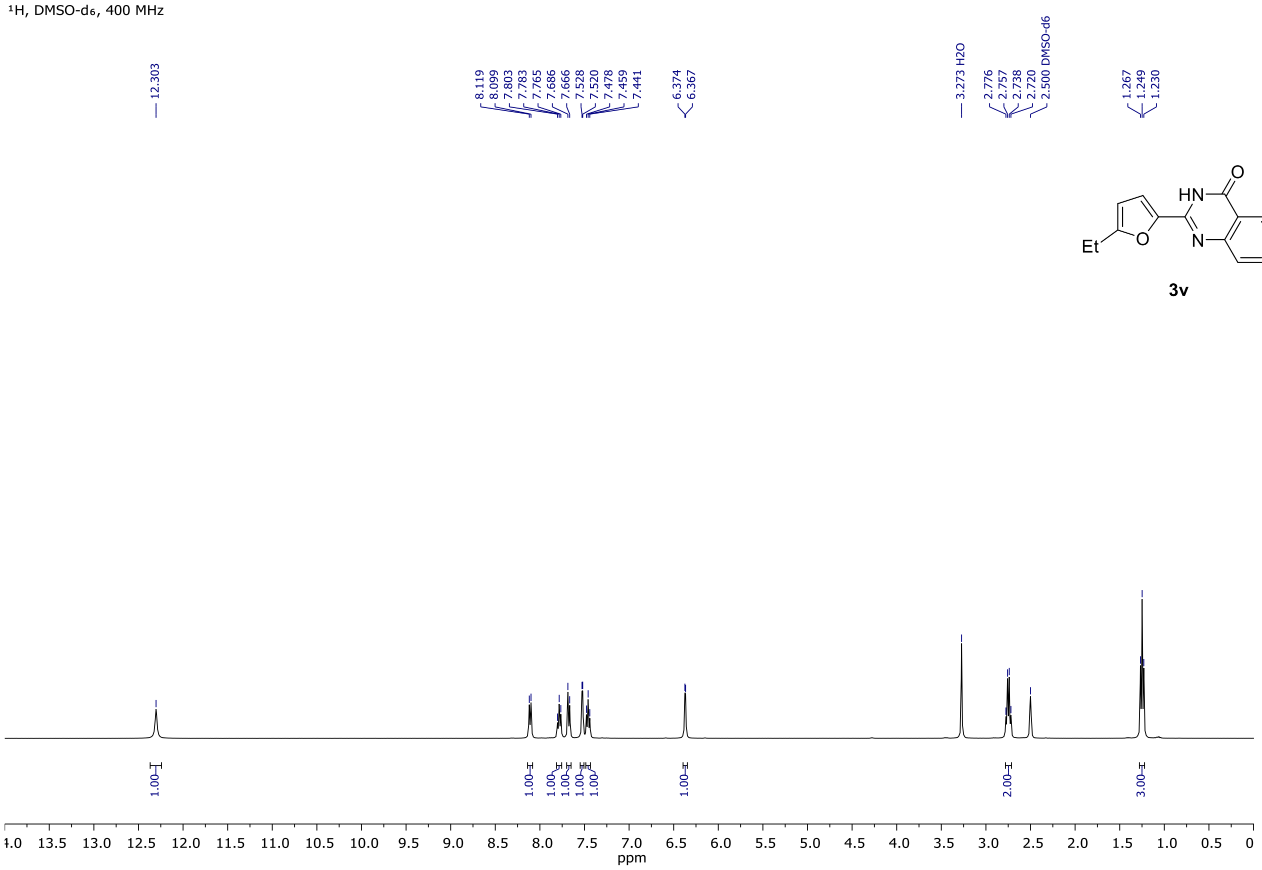

¹³C{¹H}, DMSO-d₆, 100 MHz

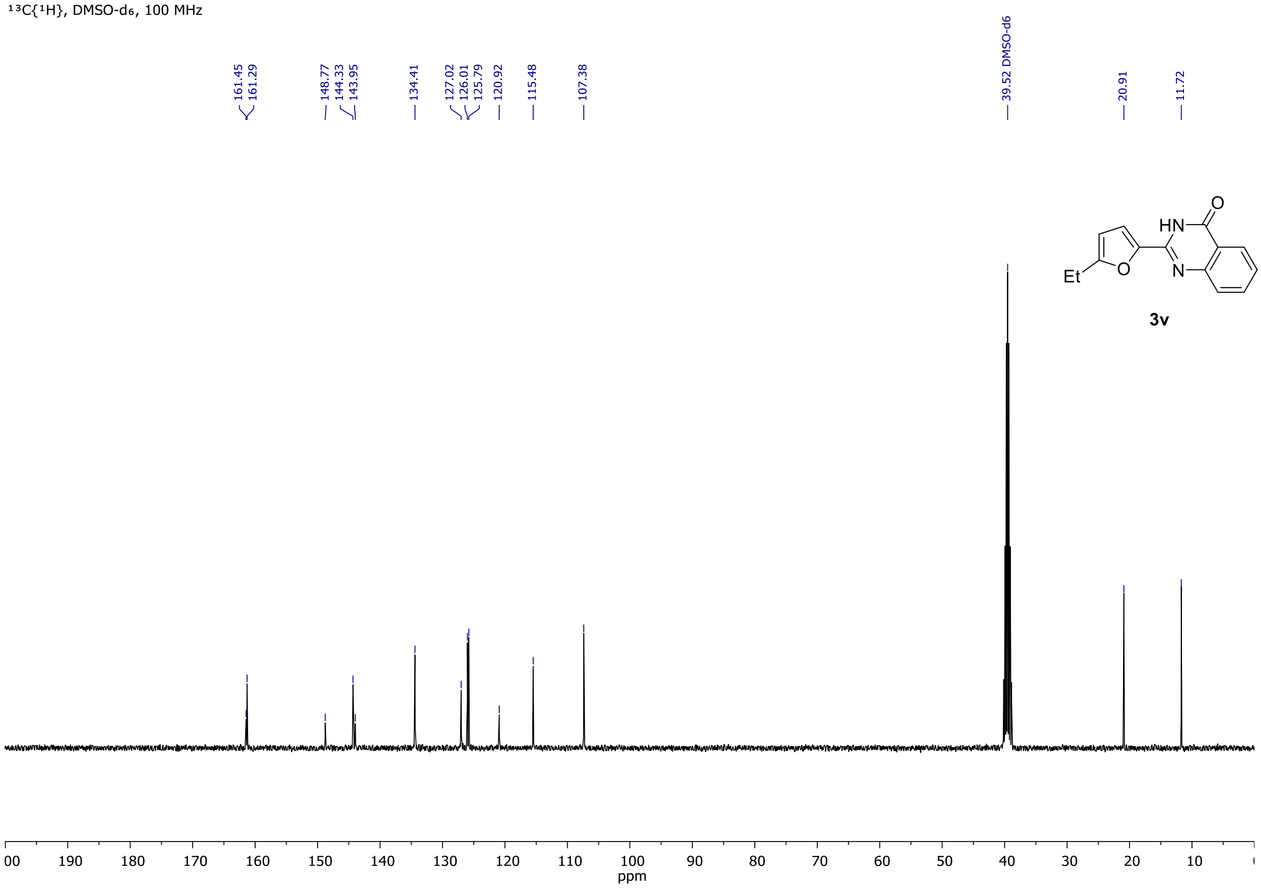

^1H, DMSO-d_6, 400 MHz

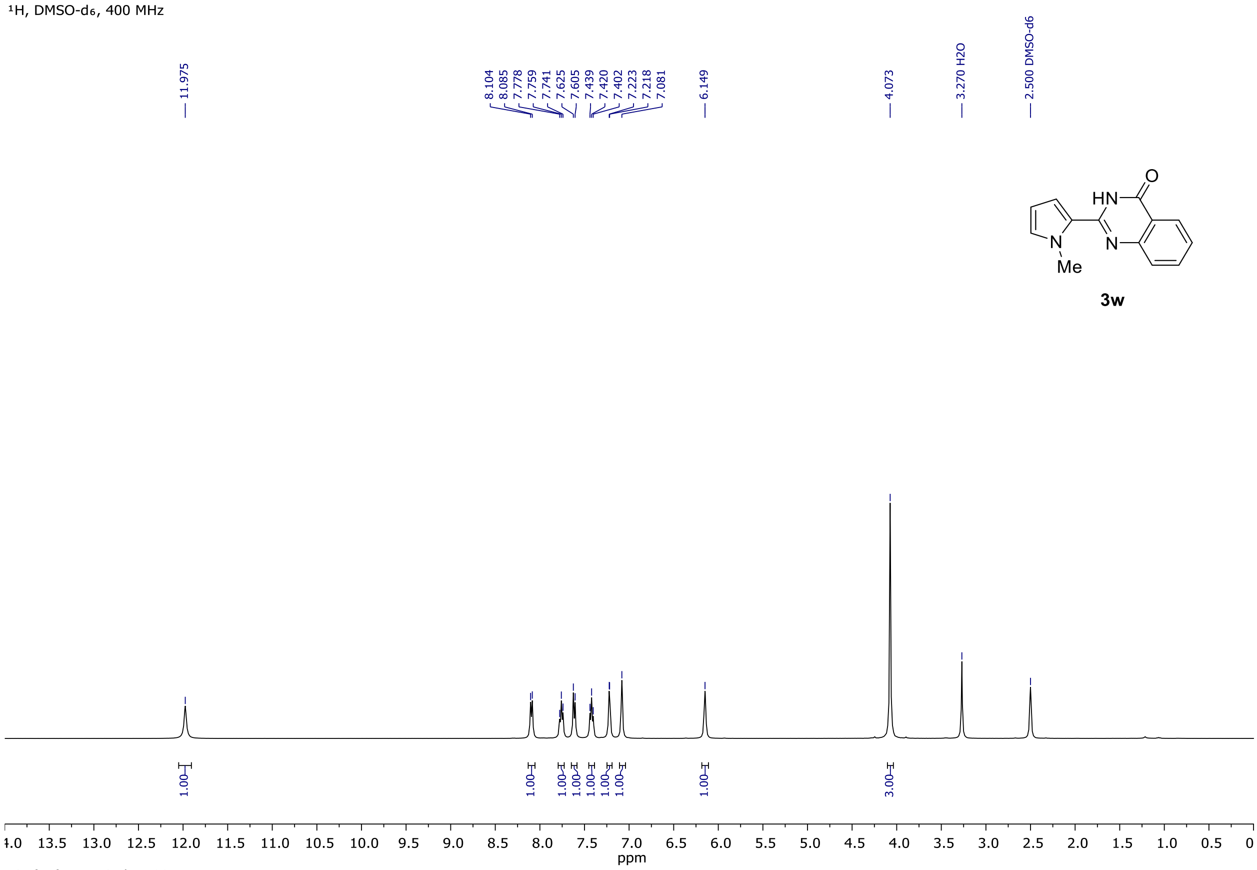

$^{13}C\{^1H\}$, DMSO-d_6, 100 MHz

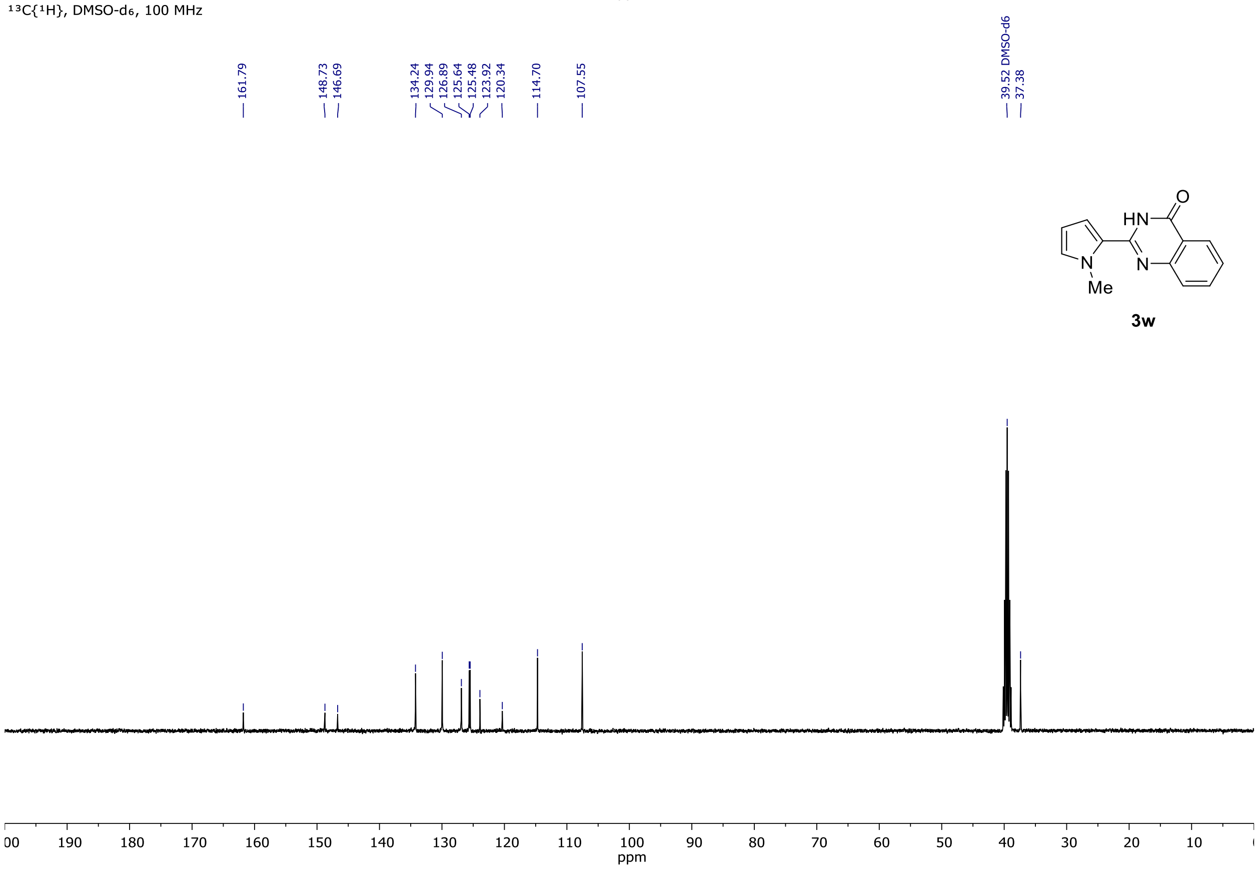

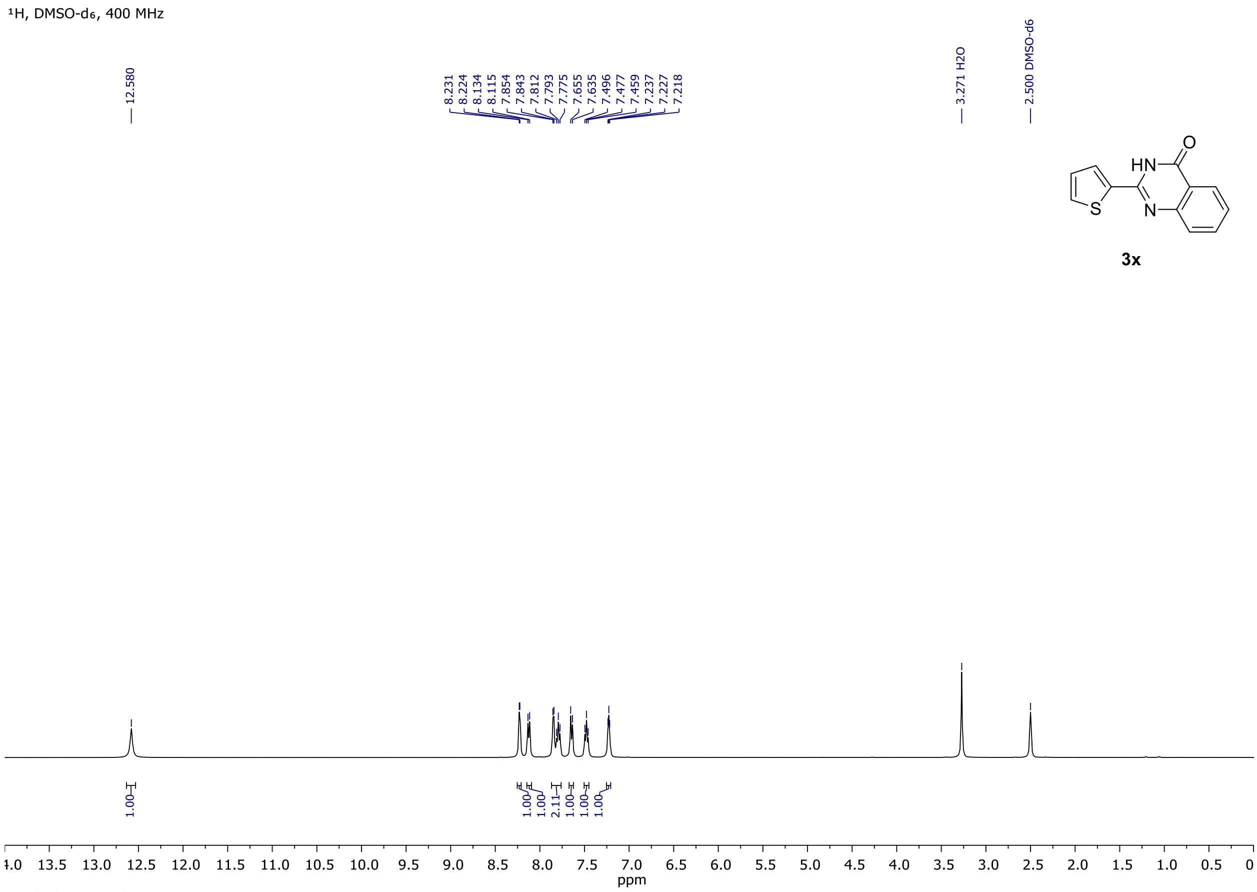

¹H, DMSO-d6, 400 MHz
12.580
8.231
8.224
8.134
8.115
7.854
7.843
7.812
7.793
7.775
7.655
7.635
7.496
7.477
7.459
7.237
7.227
7.218
3.271 H2O
2.500 DMSO-d6
1.00
1.00
1.00
2.11
1.00
1.00
1.00
ppm
HN
O
N
S
3x

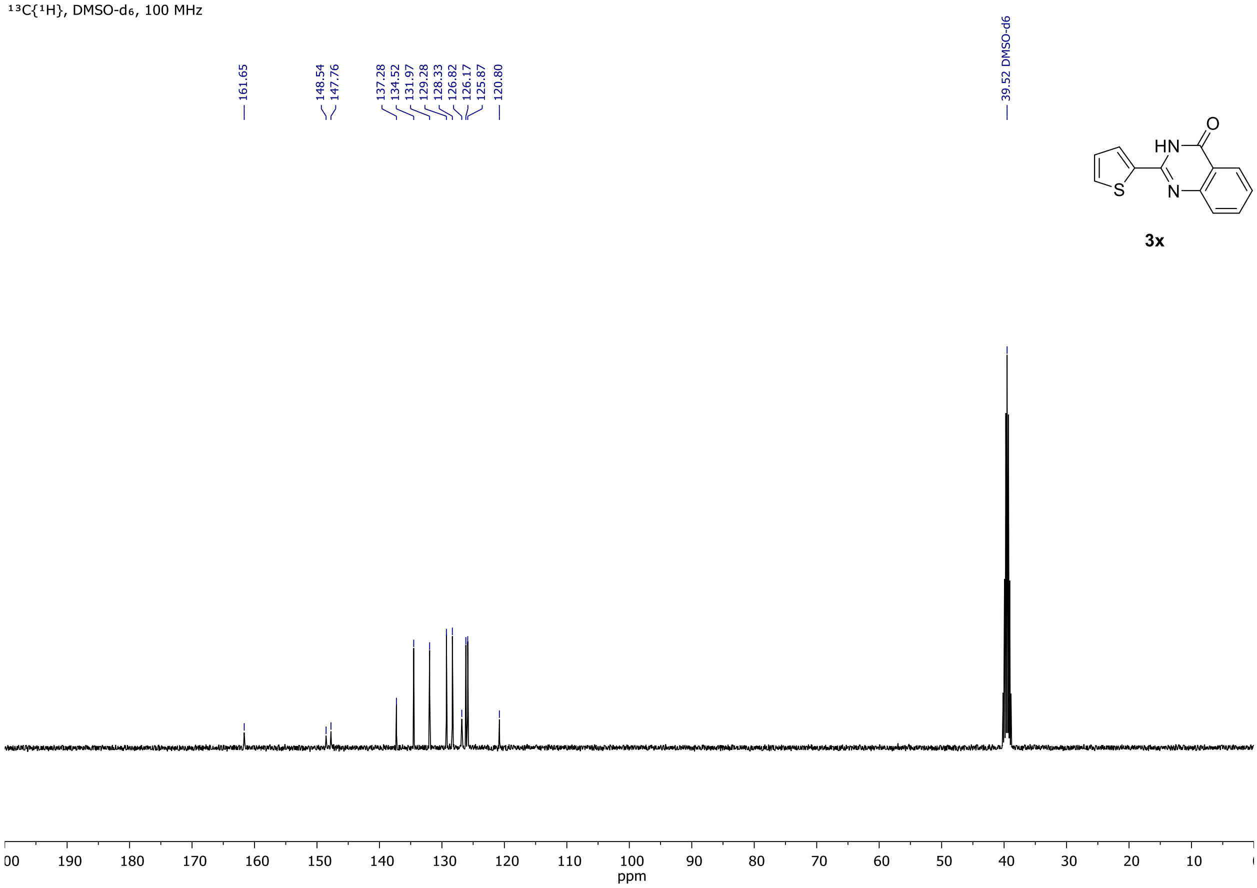

¹³C{¹H}, DMSO-d6, 100 MHz
161.65
148.54
147.76
137.28
134.52
131.97
129.28
128.33
126.82
126.17
125.87
120.80
39.52 DMSO-d6
ppm
HN
O
N
S
3x

^{1}H, DMSO-d_6, 400 MHz

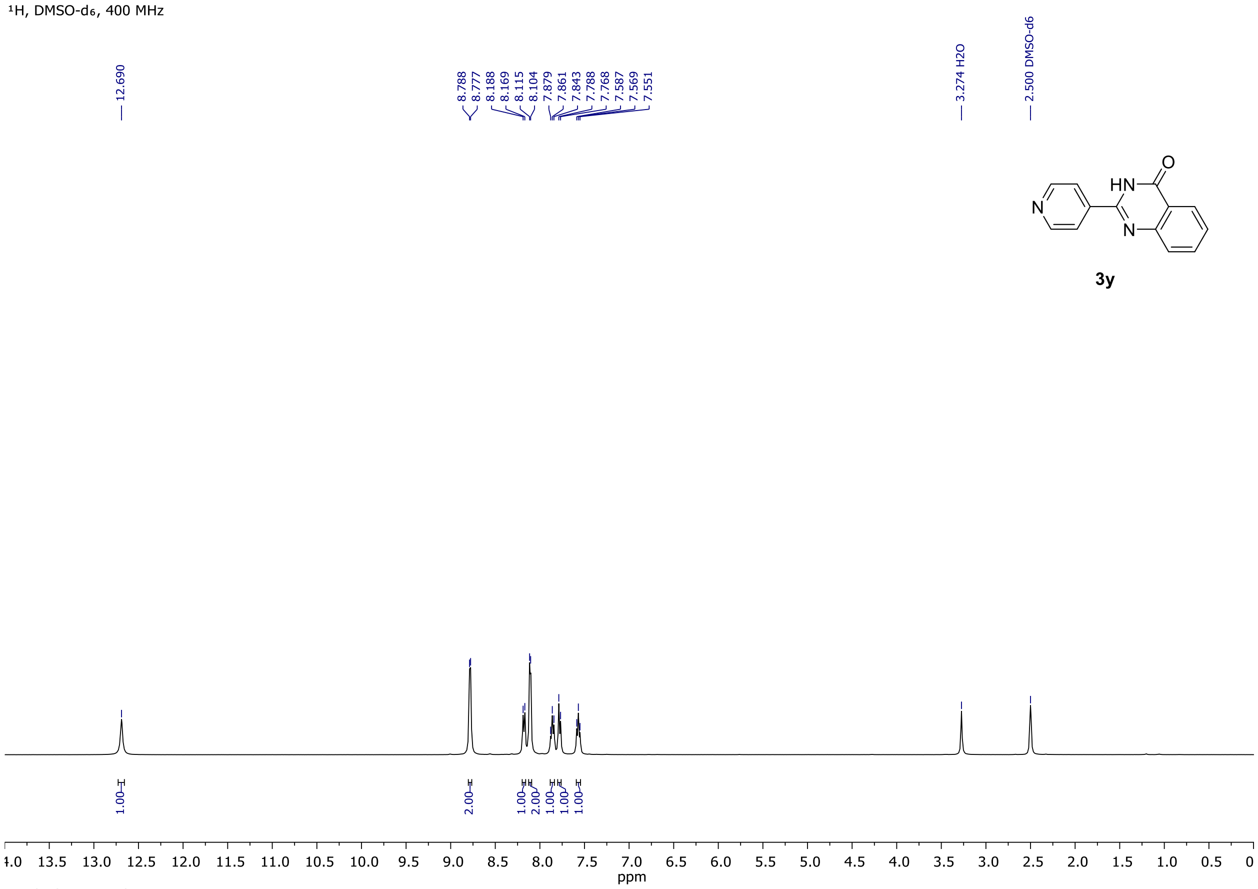

$^{13}C\{^{1}H\}$, DMSO-d_6, 100 MHz

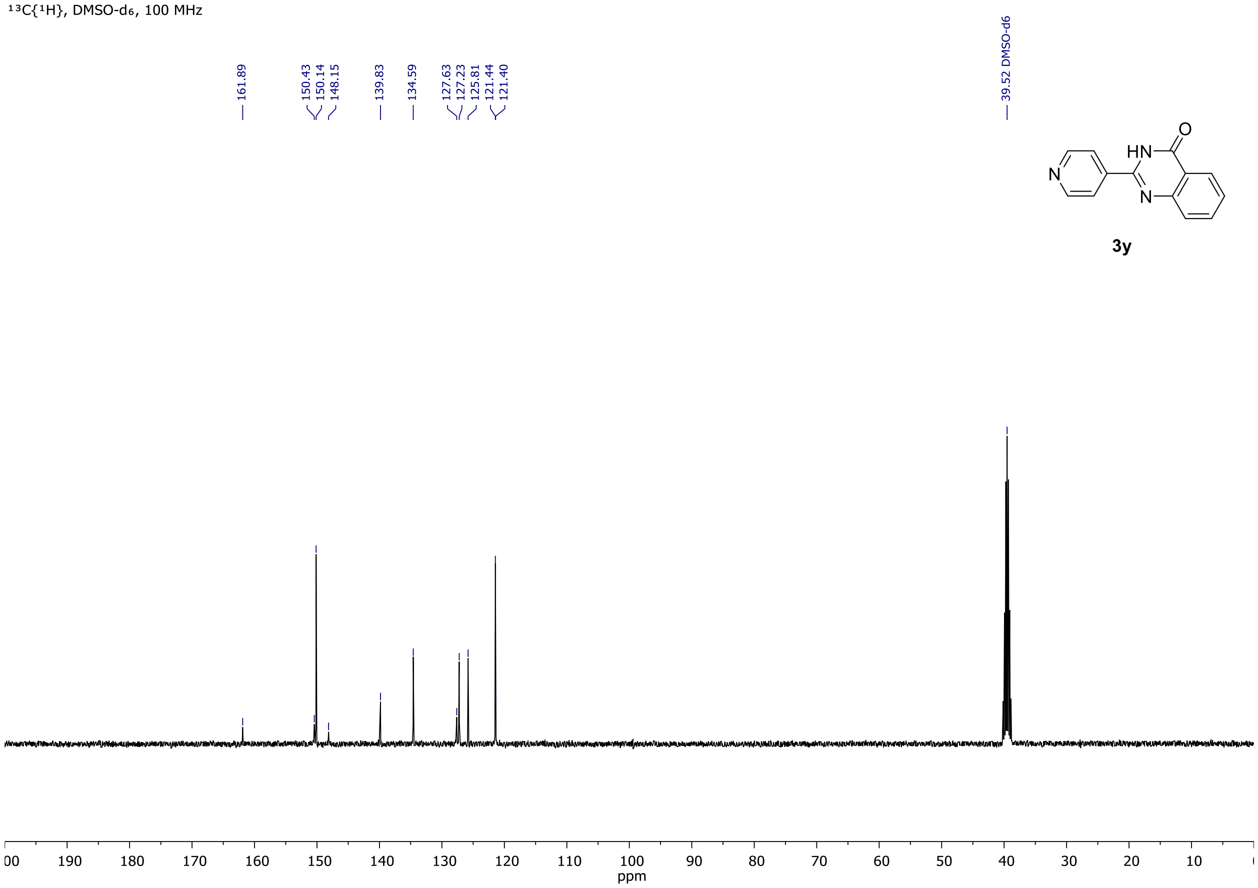

^{1}H, DMSO-d_6, 400 MHz

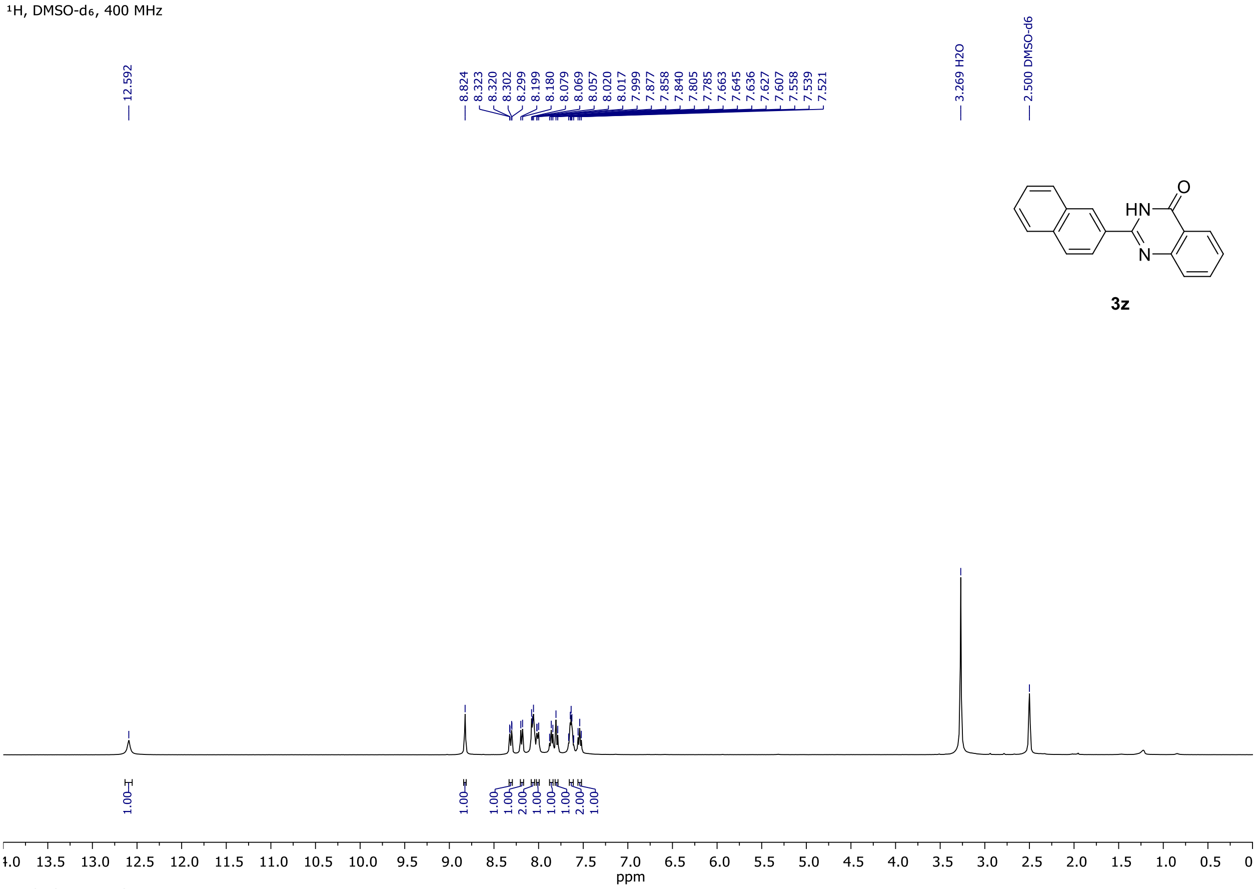

$^{13}C\{^{1}H\}$, DMSO-d_6, 100 MHz

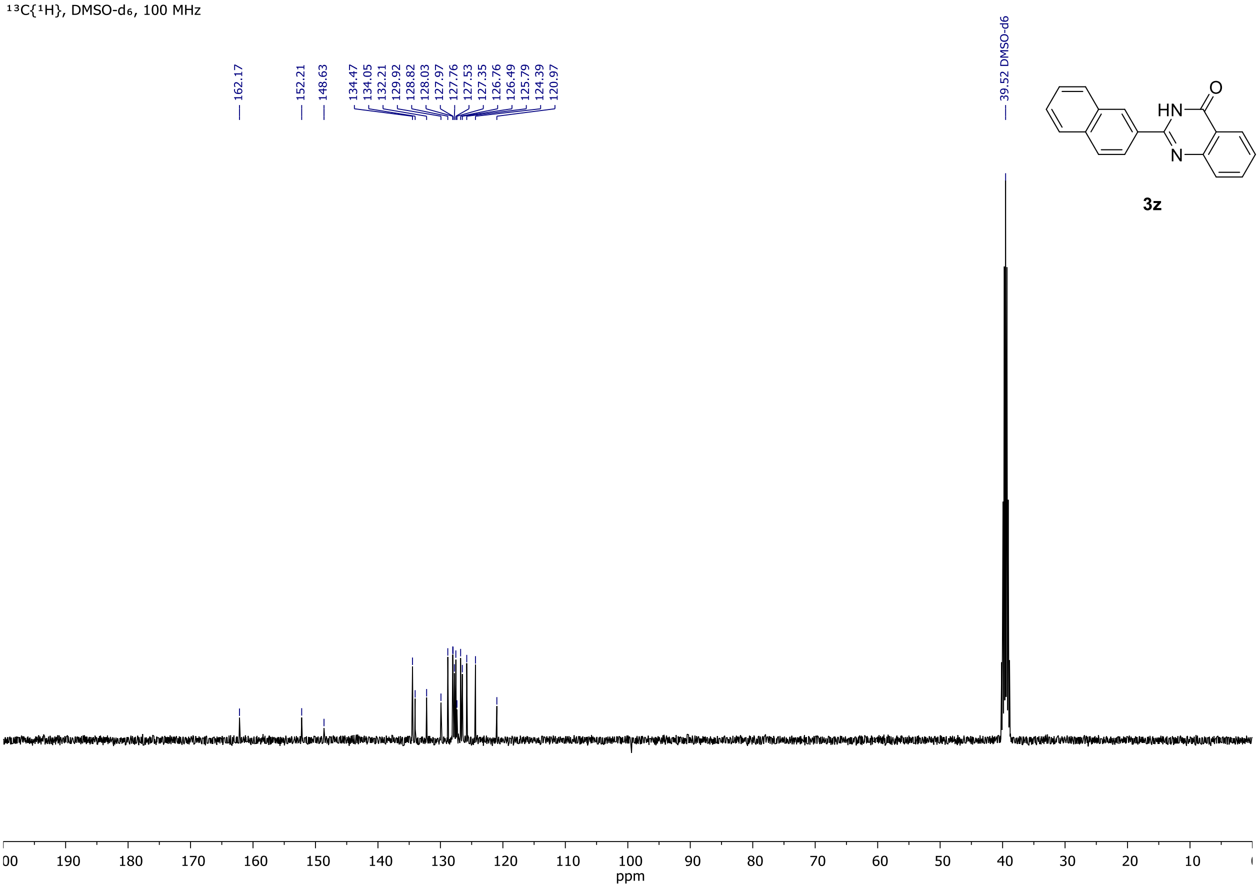

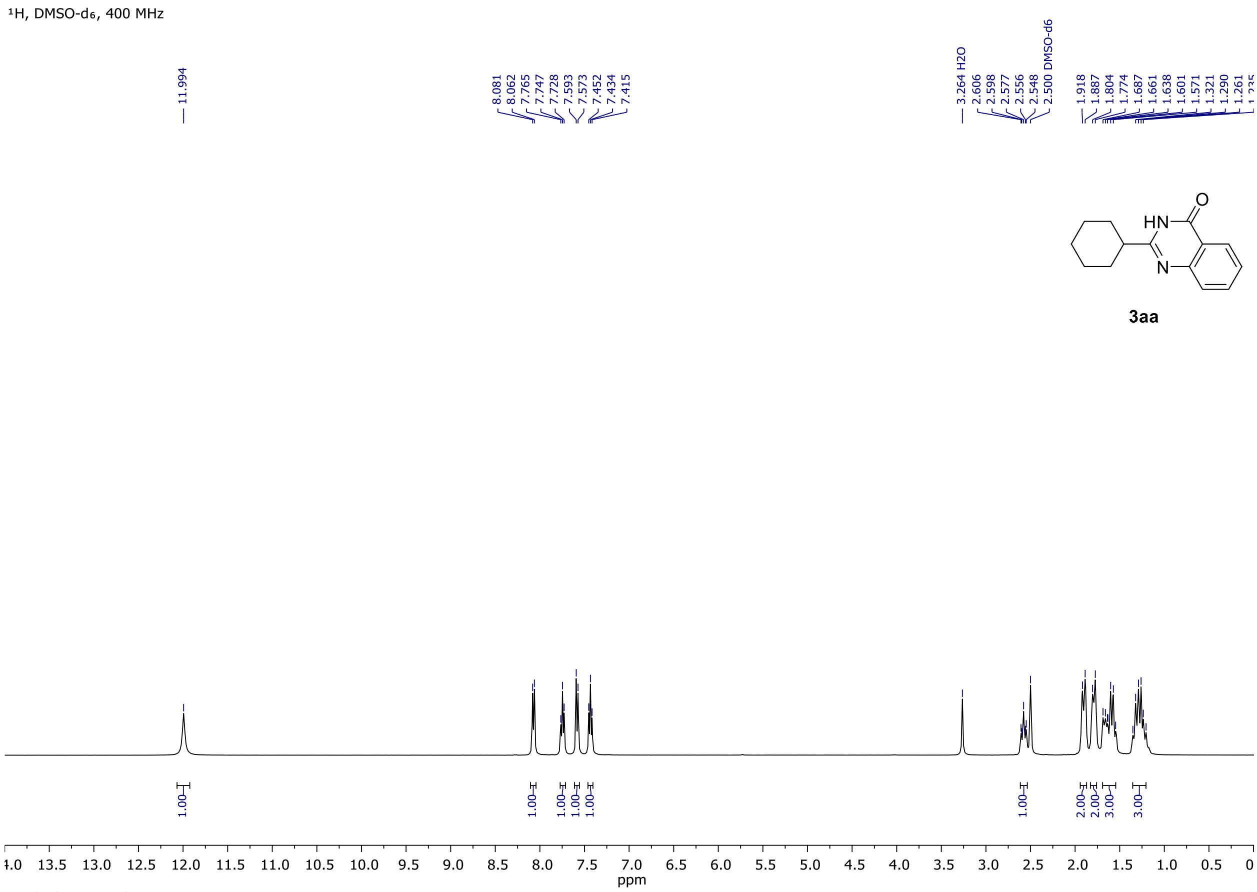

¹H, DMSO-d₆, 400 MHz
11.994
8.081 8.062 7.765 7.747 7.728 7.593 7.573 7.452 7.434 7.415
3.264 H2O
2.606 2.598 2.577 2.556 2.548 2.500 DMSO-d6
1.918 1.887 1.804 1.774 1.687 1.661 1.638 1.601 1.571 1.321 1.290 1.261
HN
O
N
3aa
1.00
1.00 1.00 1.00 1.00
1.00
2.00 2.00 3.00 3.00
ppm

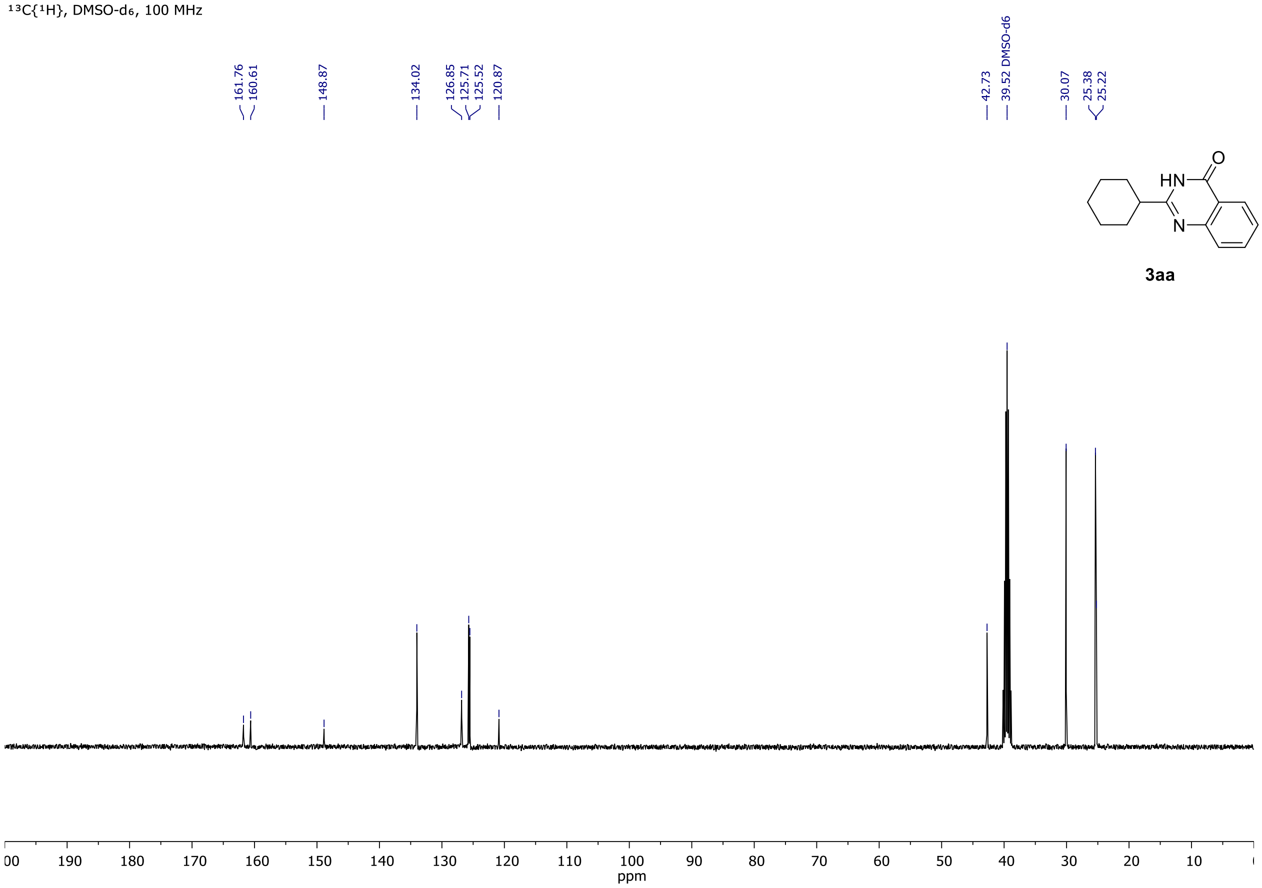

¹³C{¹H}, DMSO-d₆, 100 MHz
161.76 160.61
148.87
134.02
126.85 125.71 125.52 120.87
42.73
39.52 DMSO-d6
30.07
25.38 25.22
HN
O
N
3aa
ppm

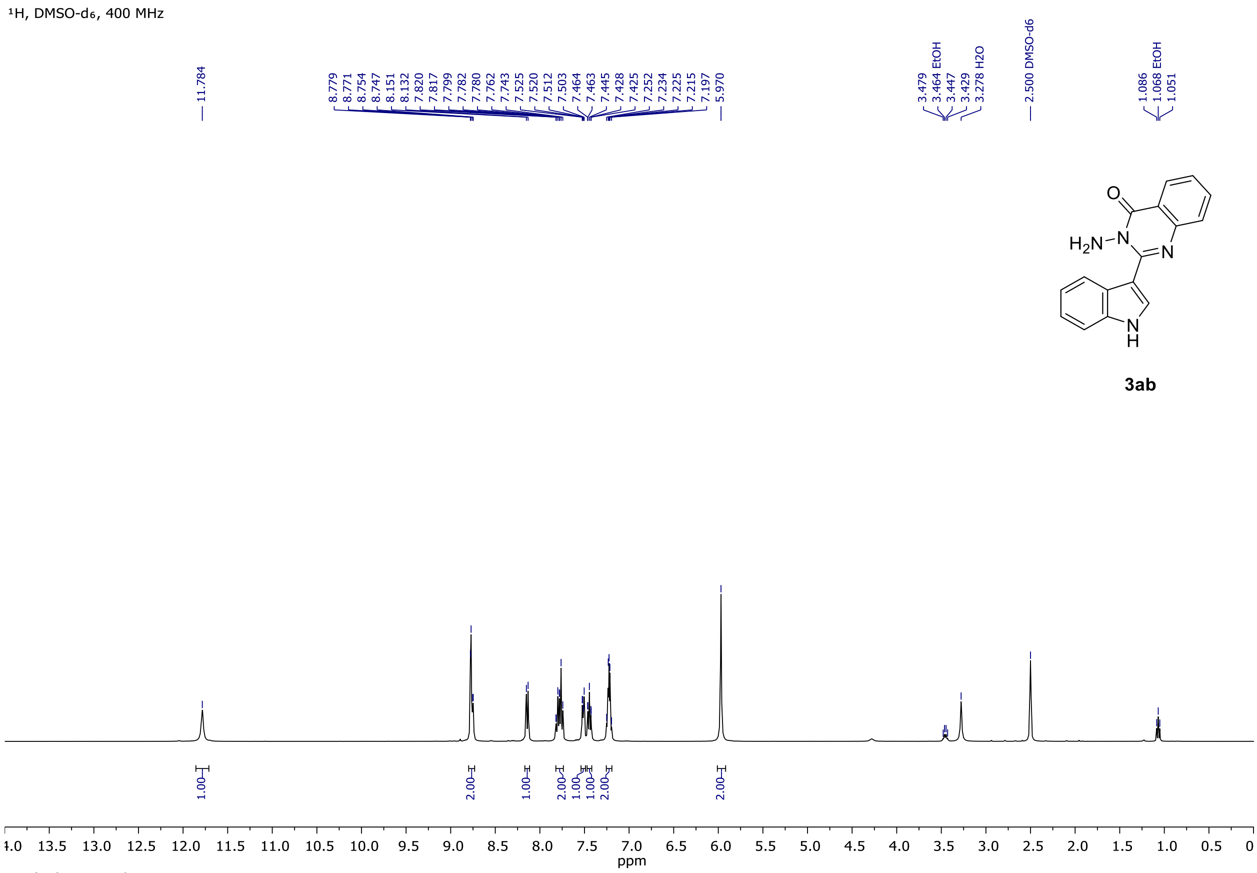
¹H, DMSO-d₆, 400 MHz
11.784
8.779 8.771 8.754 8.747 8.151 8.132 7.820 7.817 7.799 7.782 7.780 7.762 7.743 7.525 7.520 7.512 7.503 7.464 7.463 7.445 7.428 7.425 7.252 7.234 7.225 7.215 7.197
5.970
3.479 3.464 EtOH 3.447 3.429
3.278 H2O
2.500 DMSO-d6
1.086 1.068 EtOH 1.051
1.00 2.00 1.00 2.00 1.00 1.00 2.00 2.00
ppm
3ab

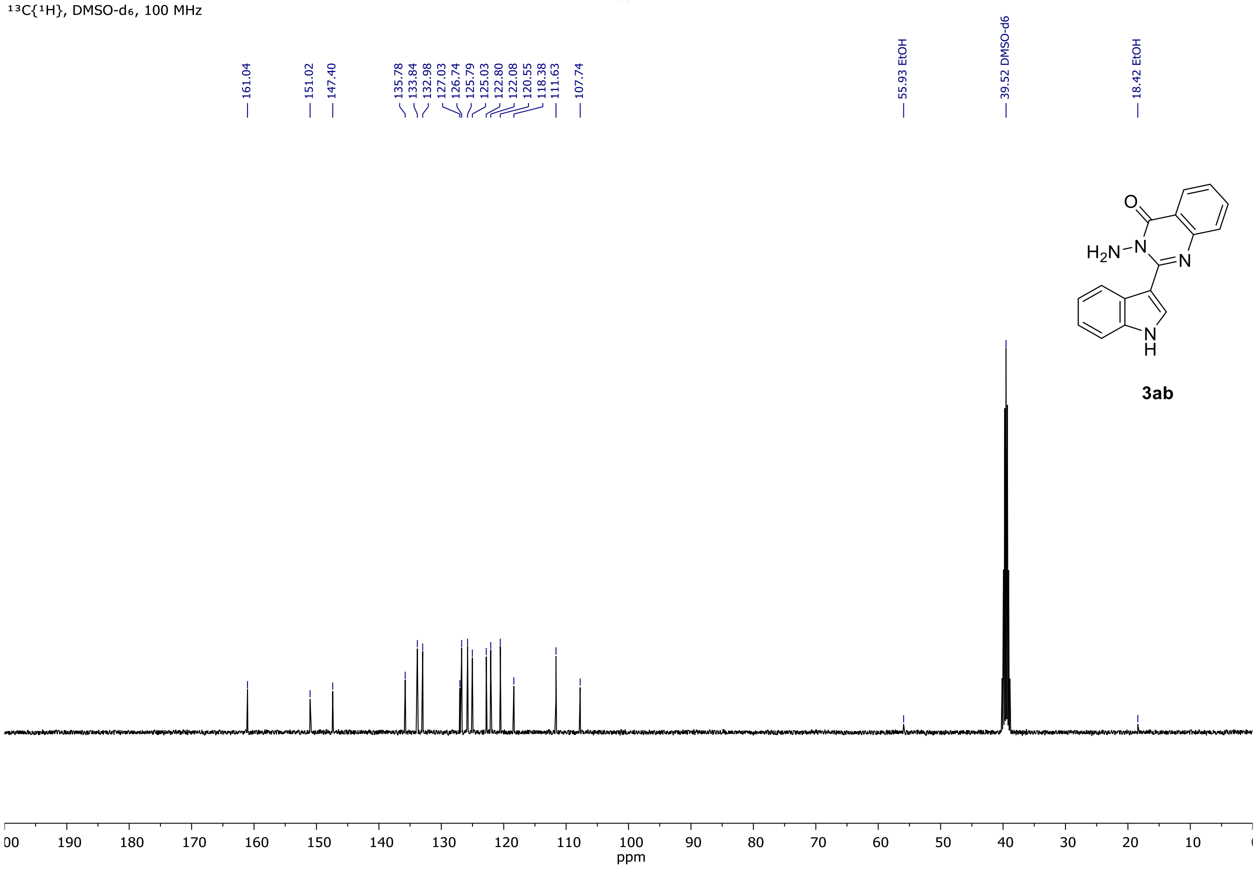
¹³C{¹H}, DMSO-d₆, 100 MHz
161.04
151.02
147.40
135.78 133.84 132.98 127.03 126.74 125.79 125.03 122.80 122.08 120.55 118.38 111.63
107.74
55.93 EtOH
39.52 DMSO-d6
18.42 EtOH
ppm
3ab

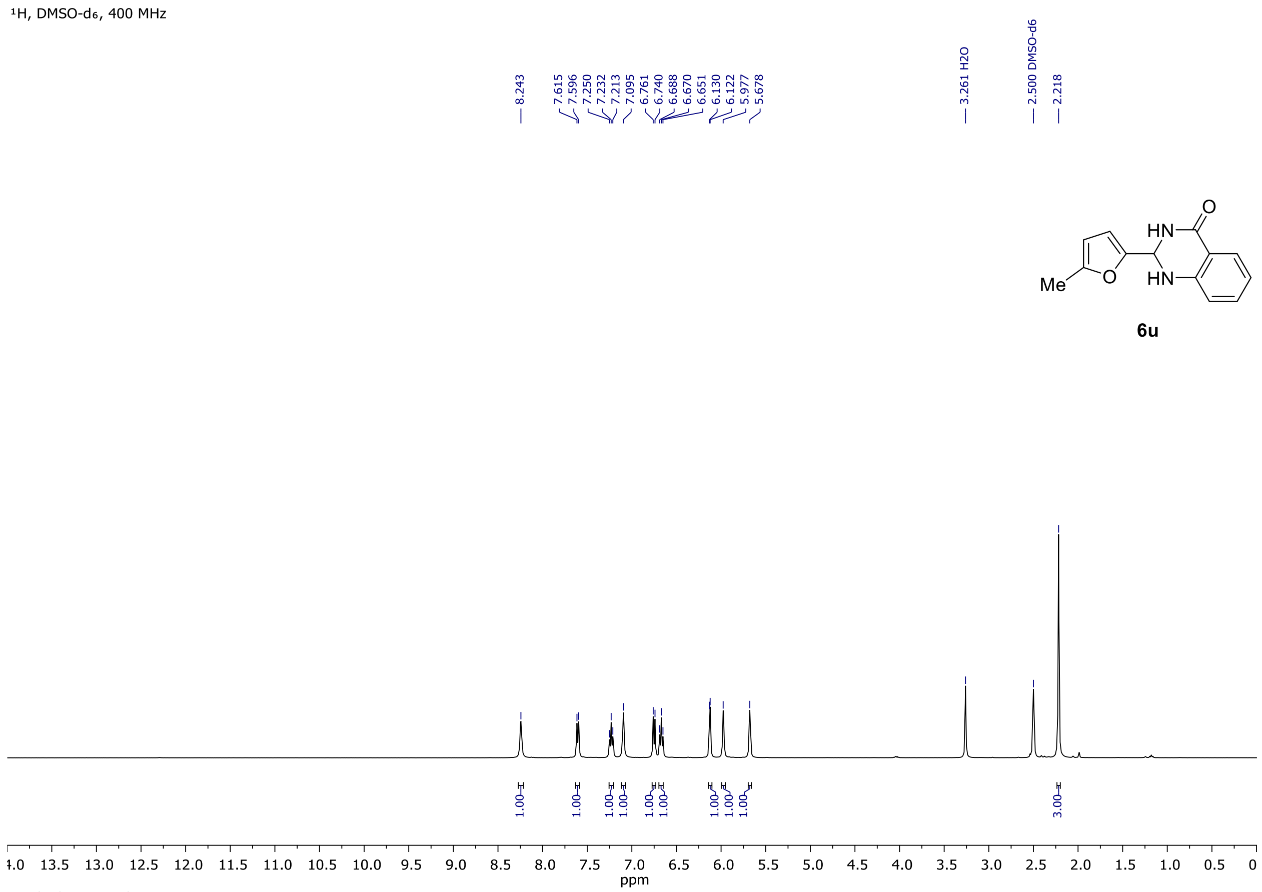

¹H, DMSO-d₆, 400 MHz
8.243
7.615
7.596
7.250
7.232
7.213
7.095
6.761
6.740
6.688
6.670
6.651
6.130
6.122
5.977
5.678
3.261 H2O
2.500 DMSO-d6
2.218
HN
O
HN
Me
O
6u
1.00
3.00
ppm

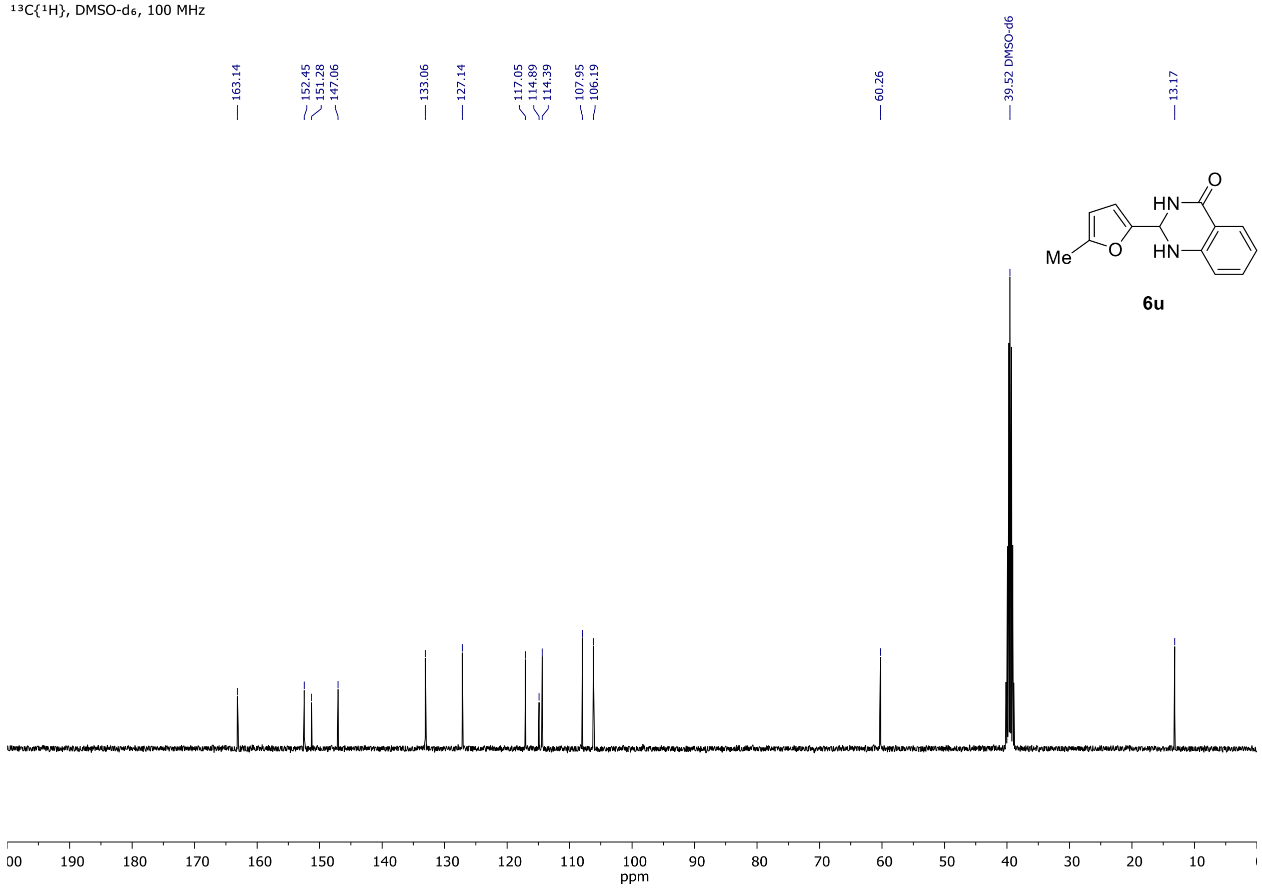

¹³C{¹H}, DMSO-d₆, 100 MHz
163.14
152.45
151.28
147.06
133.06
127.14
117.05
114.89
114.39
107.95
106.19
60.26
39.52 DMSO-d6
13.17
HN
O
HN
Me
O
6u
ppm

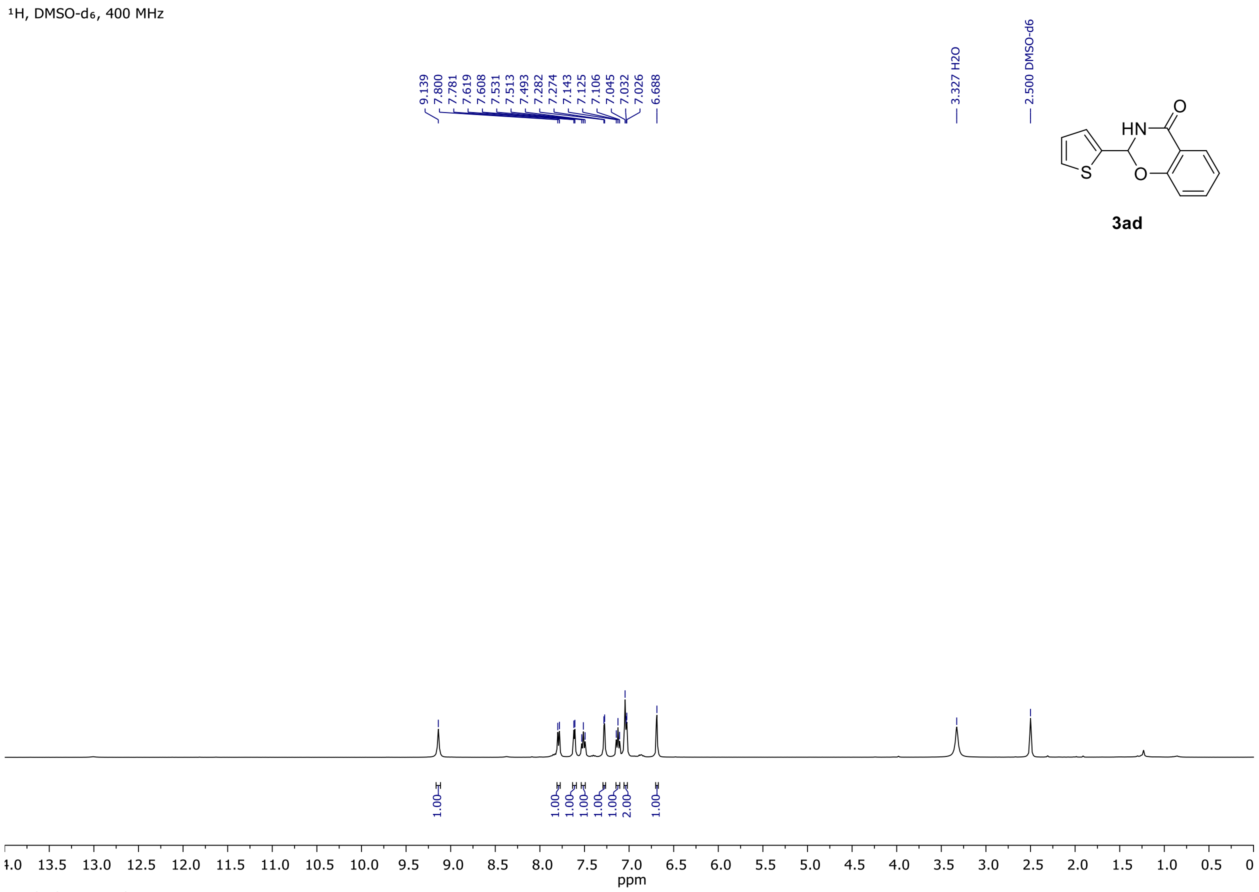

¹H, DMSO-d₆, 400 MHz
9.139
7.800
7.781
7.619
7.608
7.531
7.513
7.493
7.282
7.274
7.143
7.125
7.106
7.045
7.032
7.026
6.688
3.327 H2O
2.500 DMSO-d6
HN
O
S
O
3ad
1.00
1.00
1.00
1.00
1.00
1.00
1.00
2.00
1.00
ppm

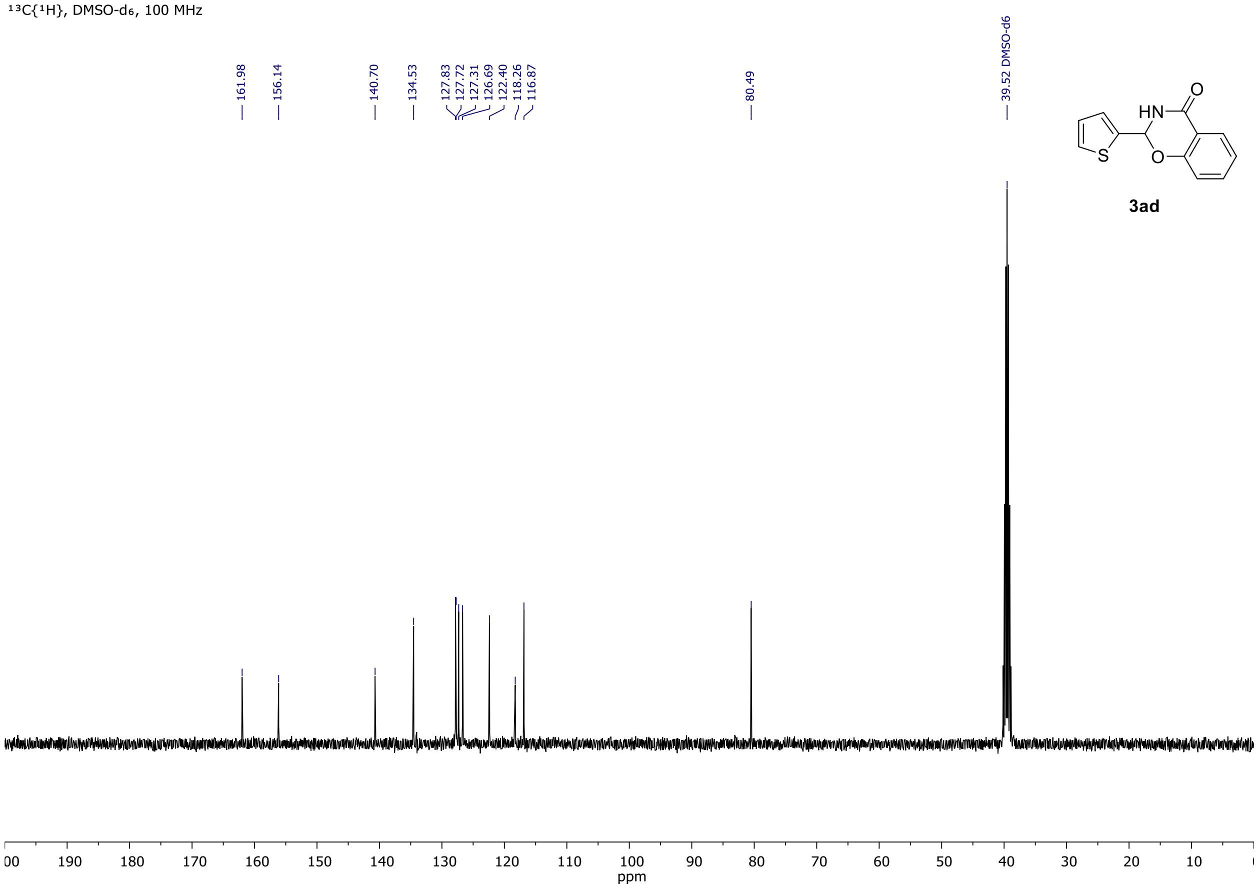

¹³C{¹H}, DMSO-d₆, 100 MHz
161.98
156.14
140.70
134.53
127.83
127.72
127.31
126.69
122.40
118.26
116.87
80.49
39.52 DMSO-d6
HN
O
S
O
3ad
ppm

^1H, DMSO-d_6, 400 MHz

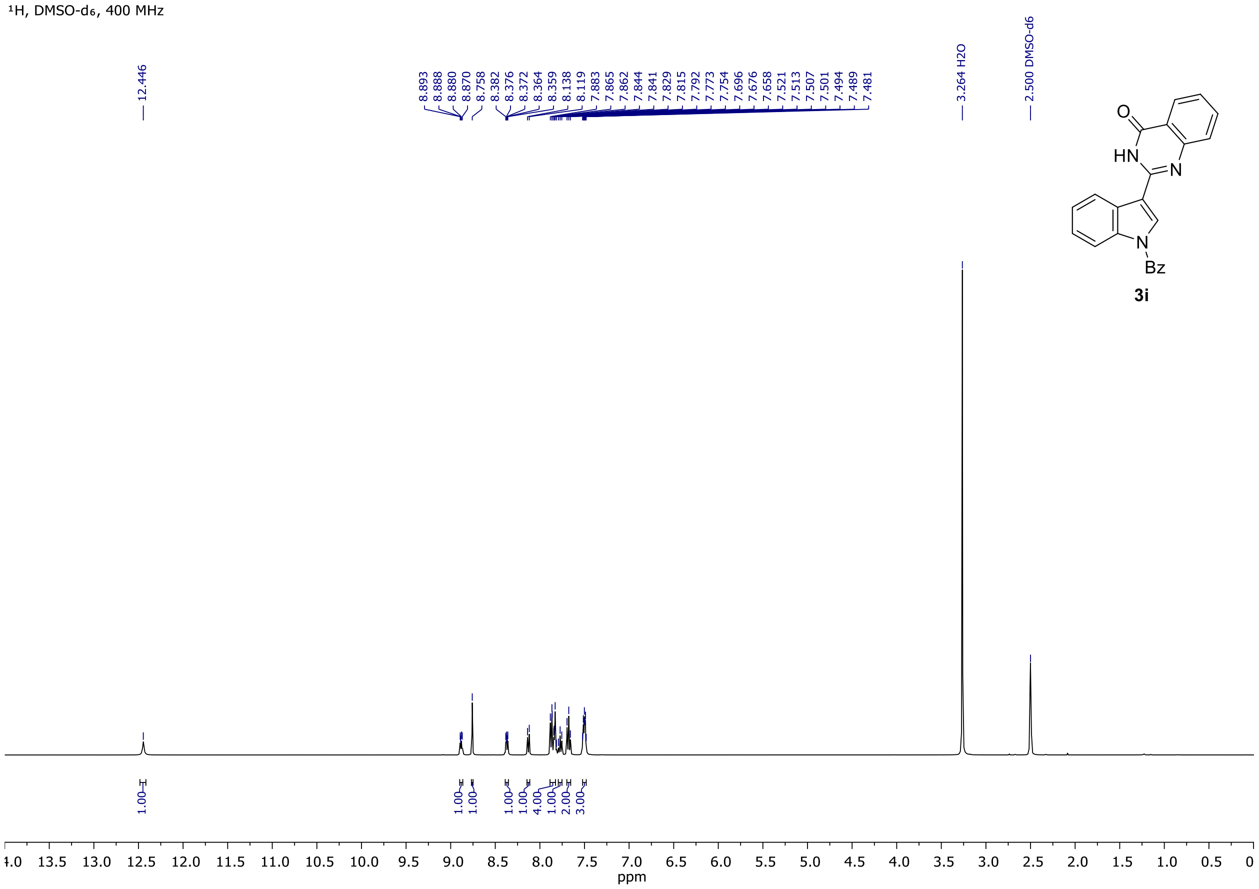

$^{13}C\{^1H\}$, DMSO-d_6, 100 MHz

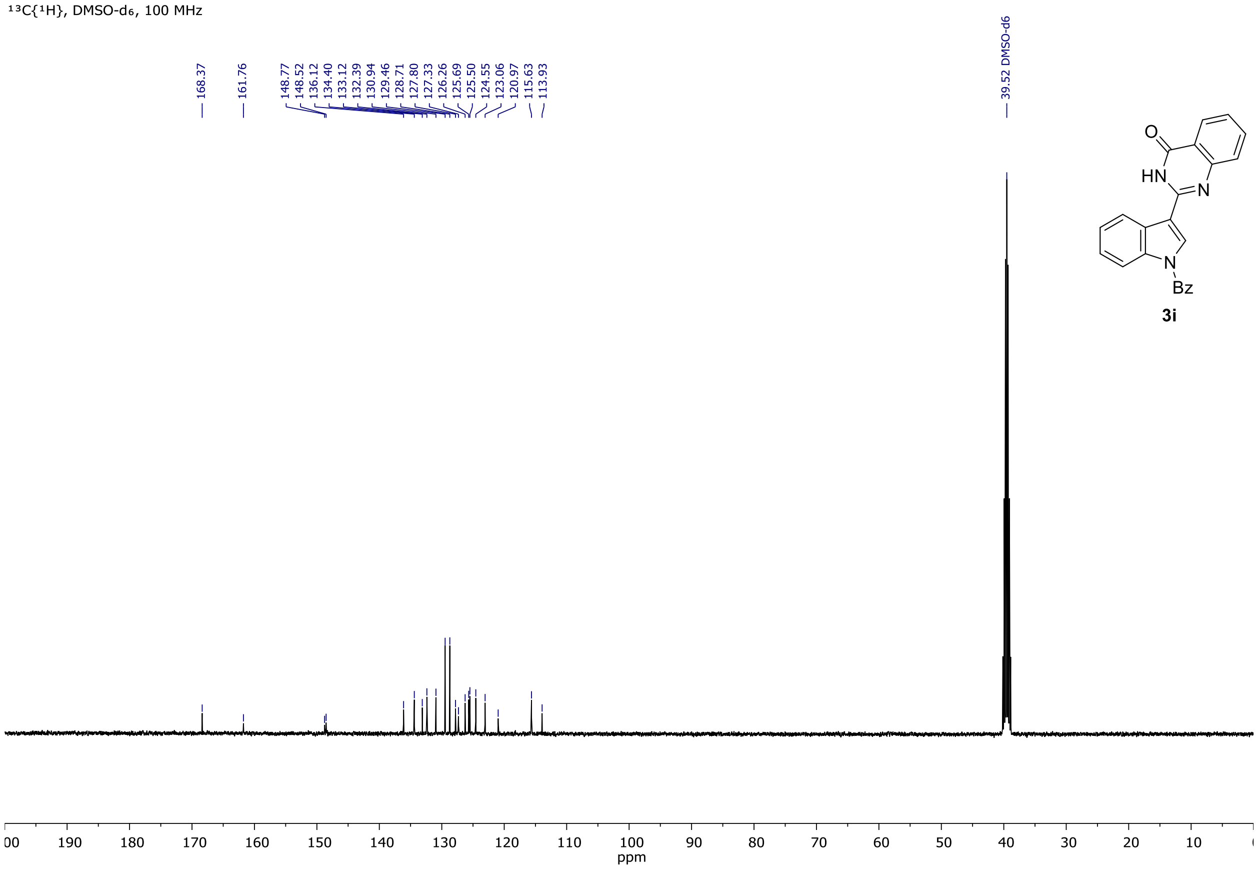

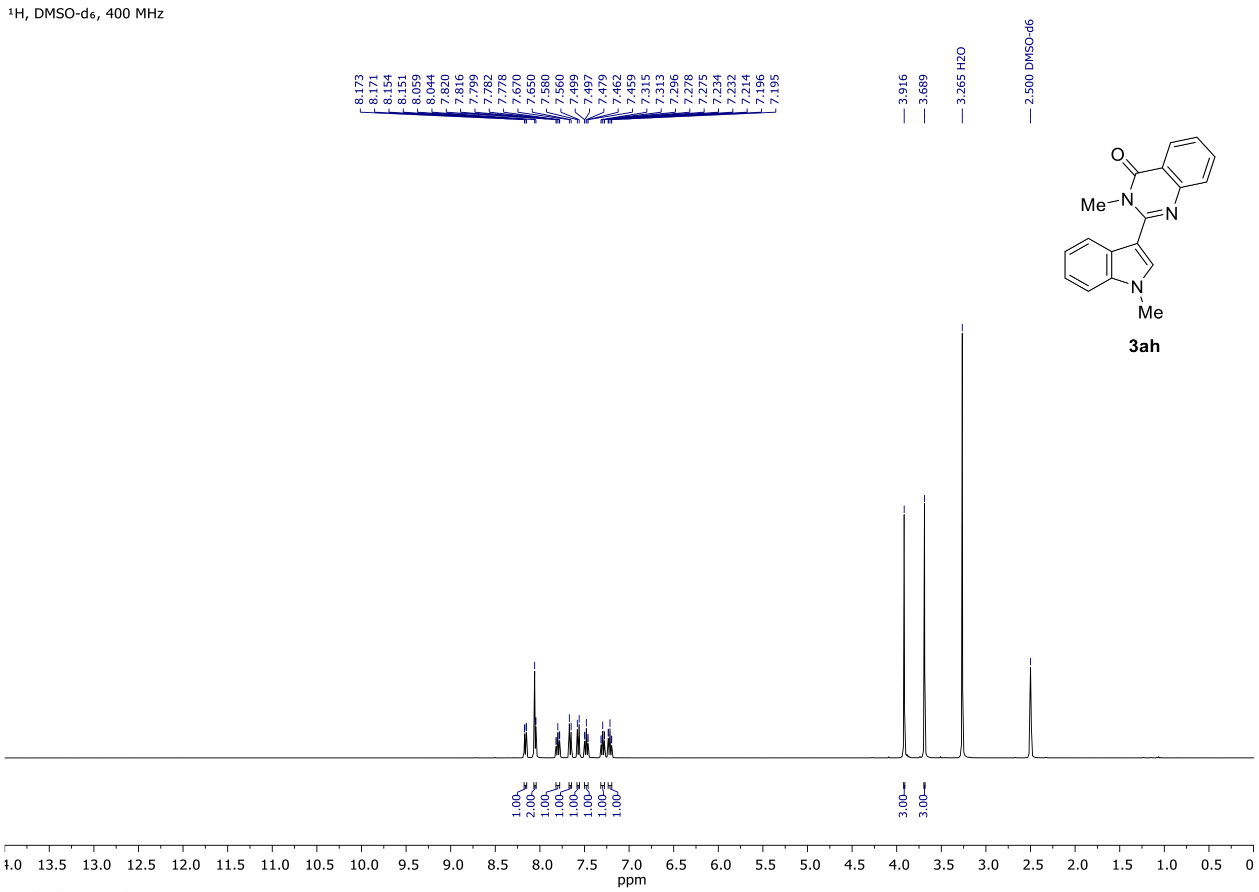
¹H, DMSO-d₆, 400 MHz
8.173
8.171
8.154
8.151
8.059
8.044
7.820
7.816
7.799
7.782
7.778
7.670
7.650
7.580
7.560
7.499
7.497
7.479
7.462
7.459
7.315
7.313
7.296
7.278
7.275
7.234
7.232
7.214
7.196
7.195
3.916
3.689
3.265 H2O
2.500 DMSO-d6
O
Me
N
N
N
Me
3ah
1.00
2.00
1.00
1.00
1.00
1.00
1.00
1.00
3.00
3.00
ppm

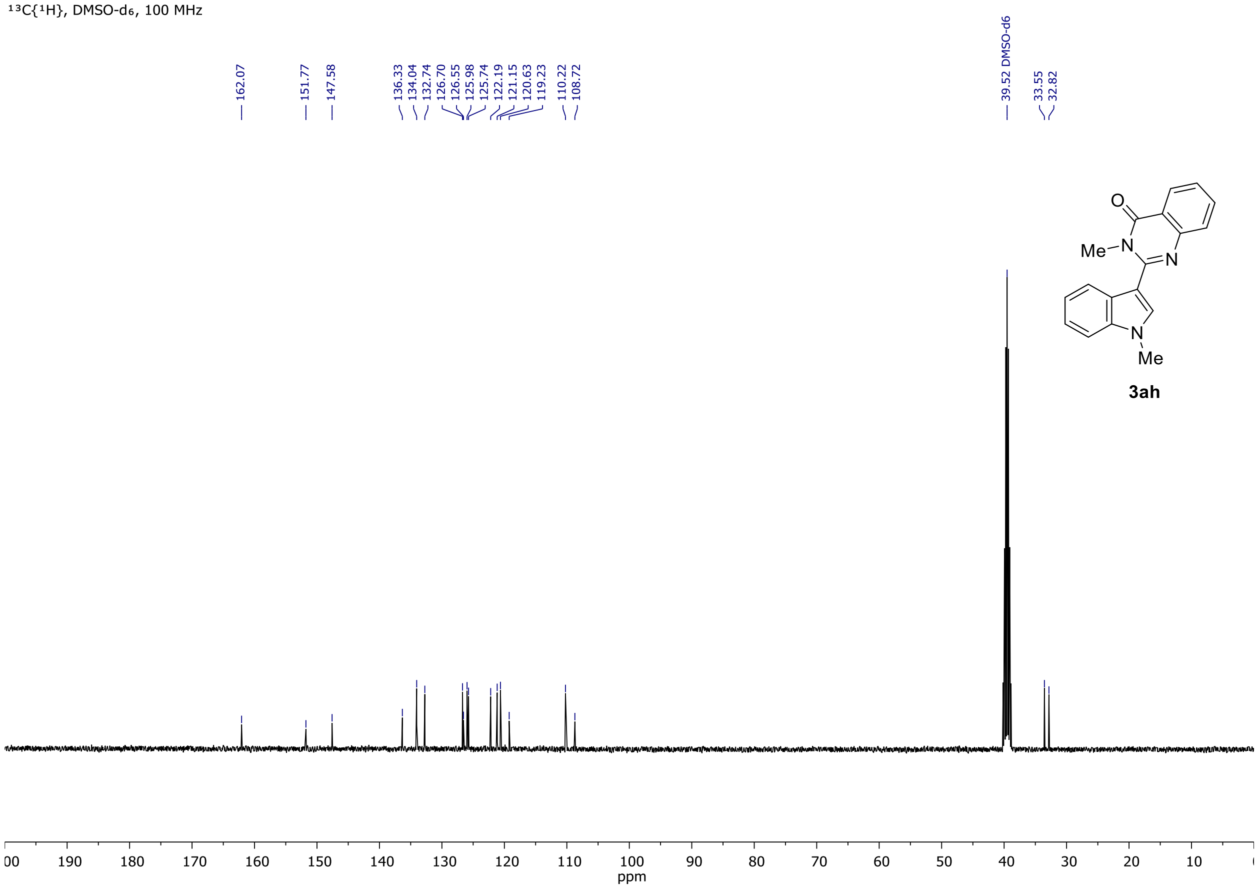
¹³C{¹H}, DMSO-d₆, 100 MHz
162.07
151.77
147.58
136.33
134.04
132.74
126.70
126.55
125.98
125.74
122.19
121.15
120.63
119.23
110.22
108.72
39.52 DMSO-d6
33.55
32.82
O
Me
N
N
N
Me
3ah
ppm

¹H, DMSO-d₆, 400 MHz

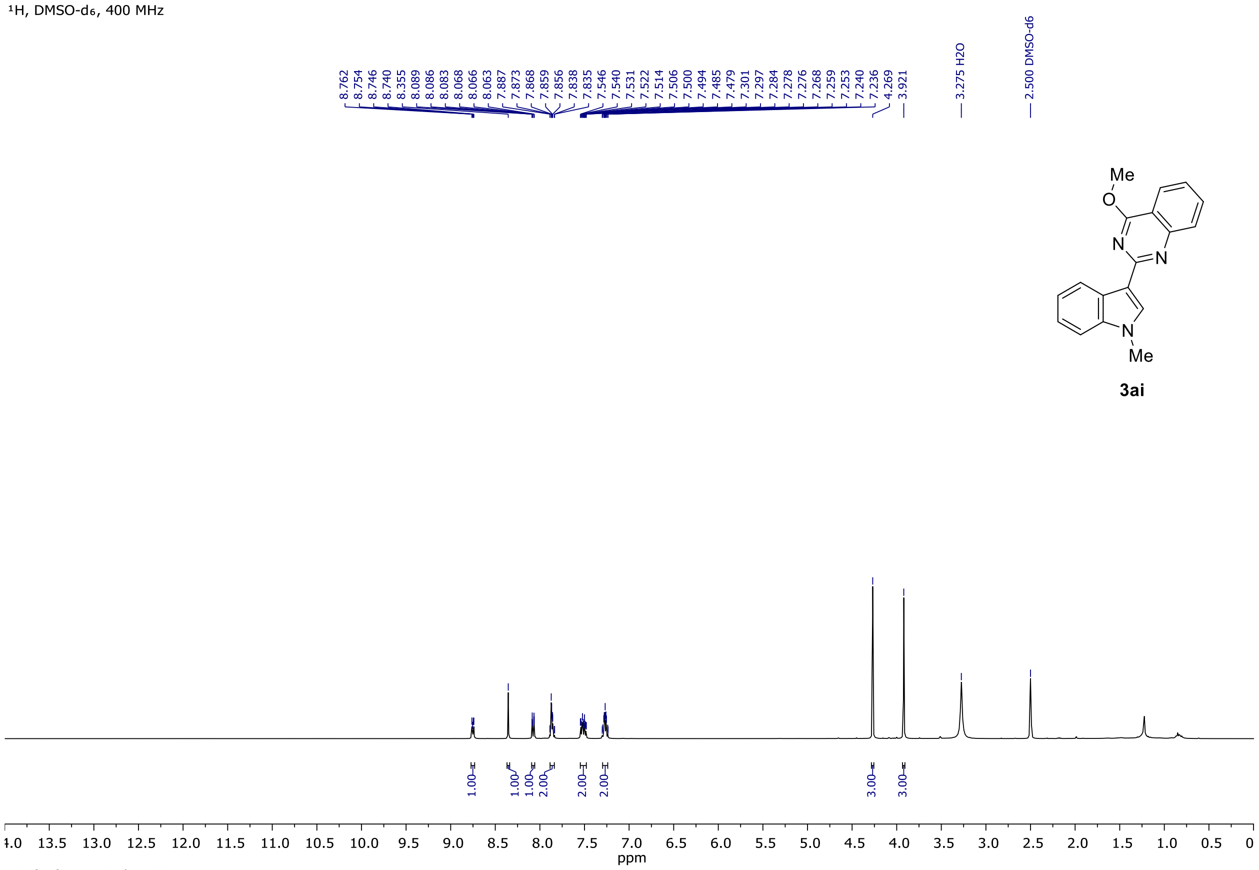

¹³C{¹H}, DMSO-d₆, 100 MHz

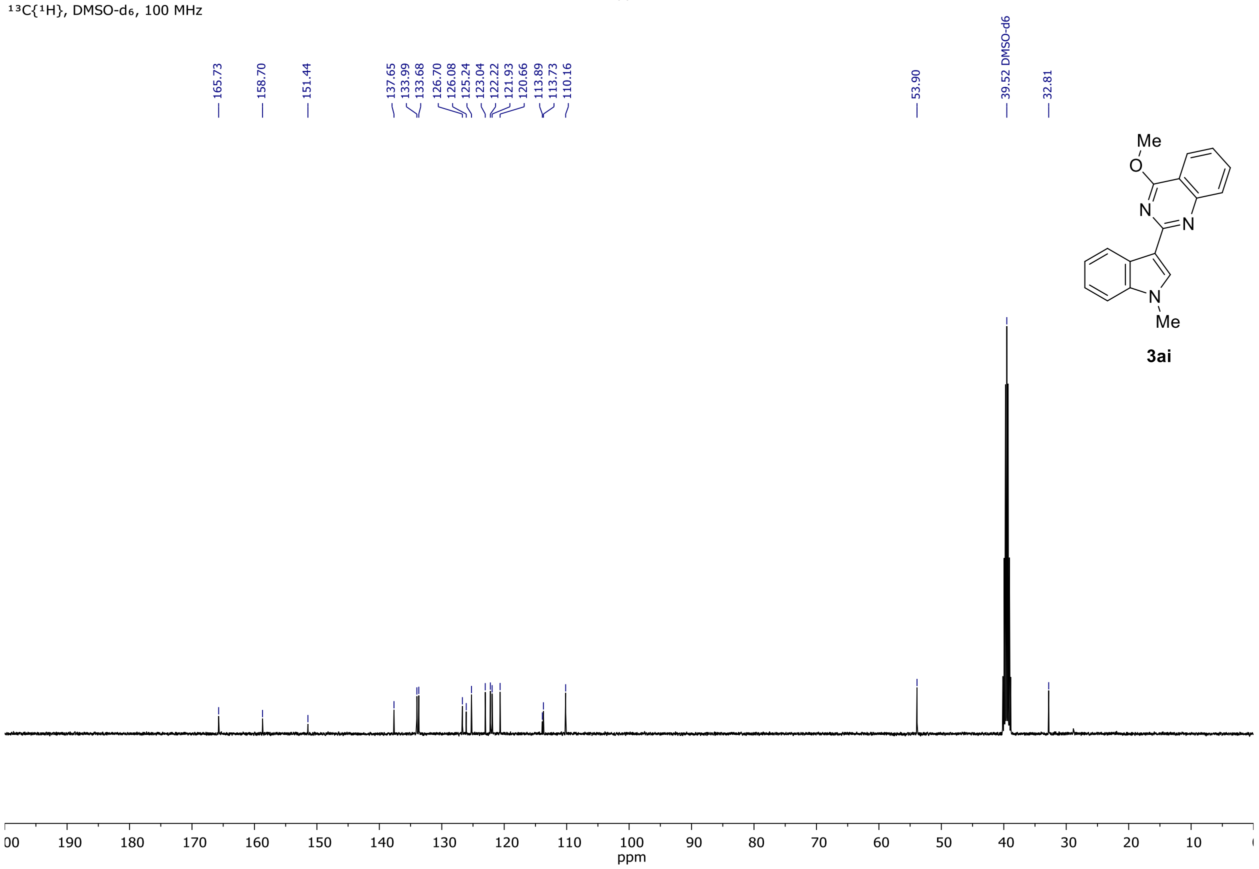

¹H, DMSO-d₆, 400 MHz

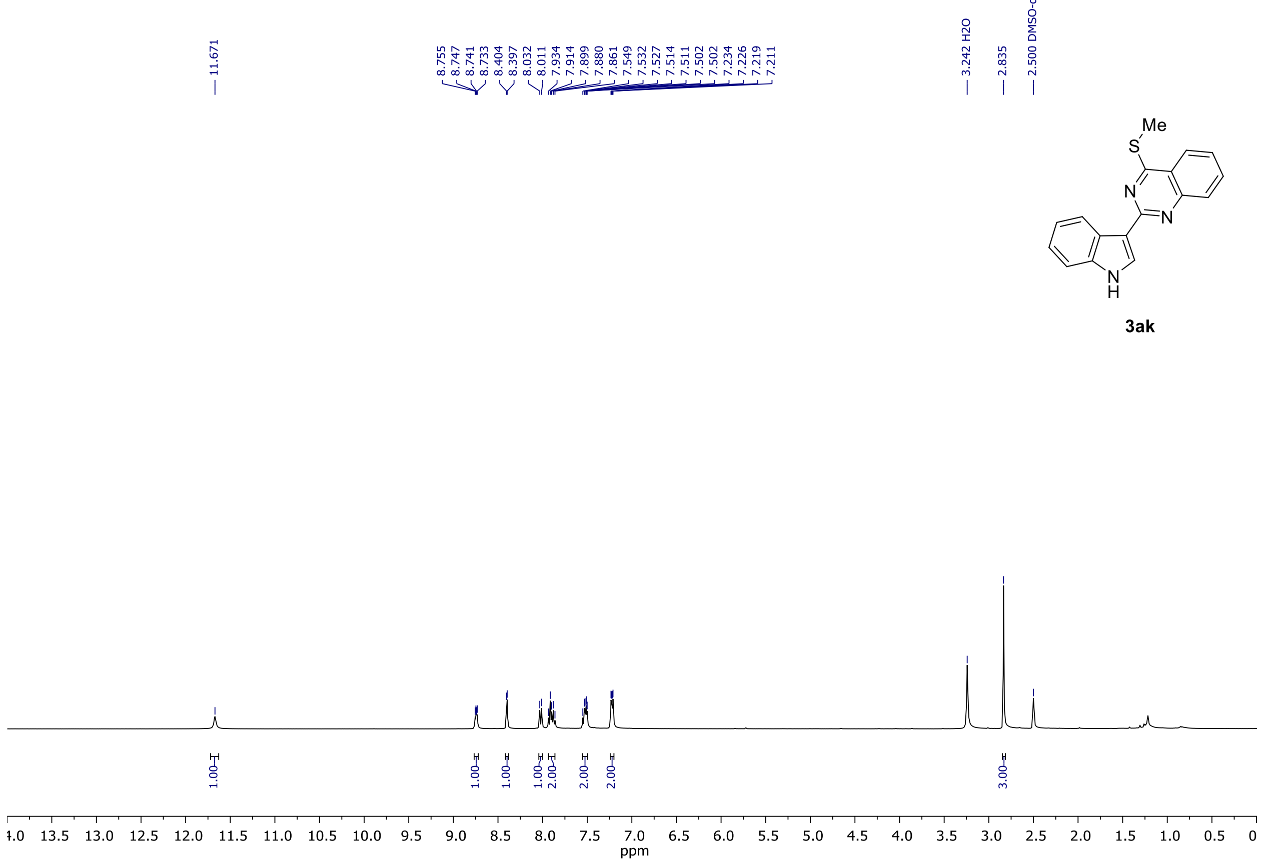

¹³C{¹H}, DMSO-d₆, 100 MHz

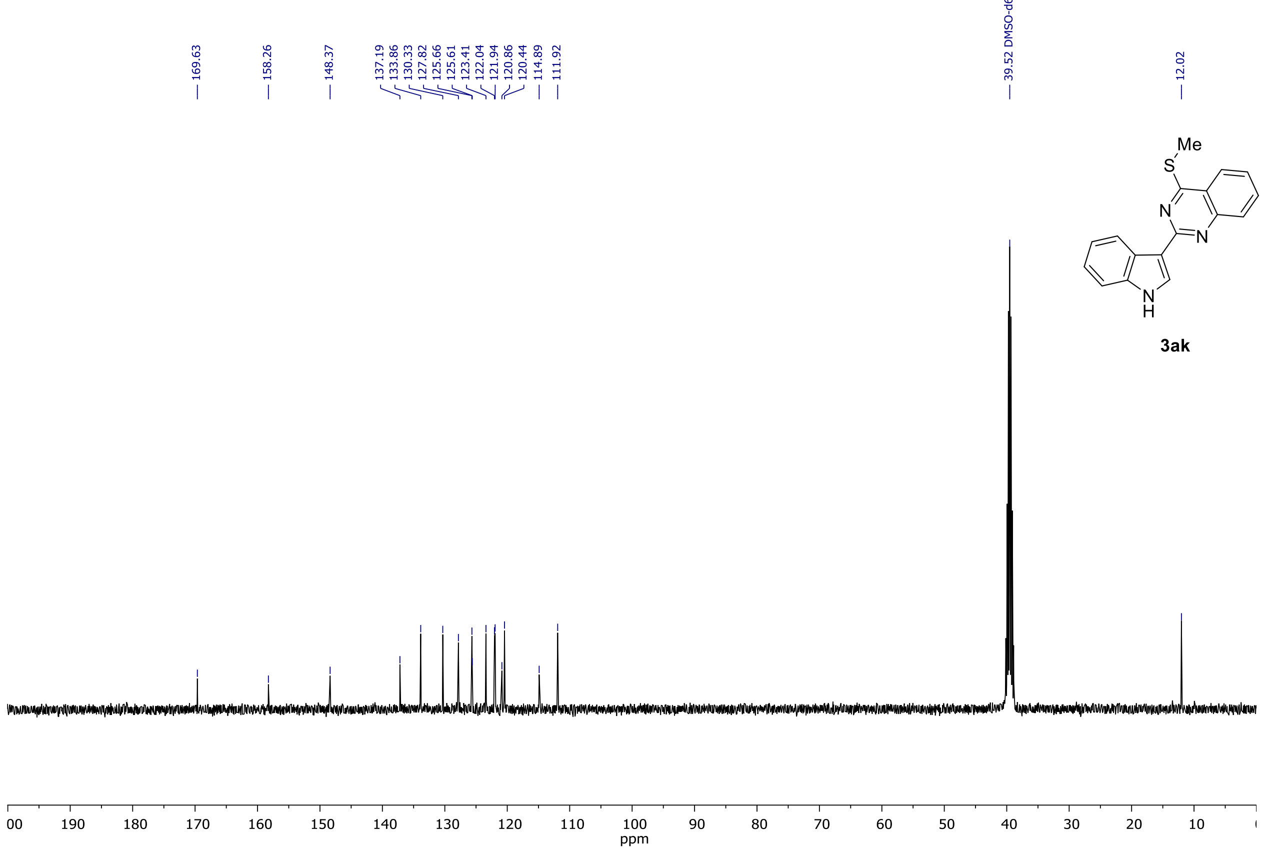

¹H, DMSO-d₆, 400 MHz

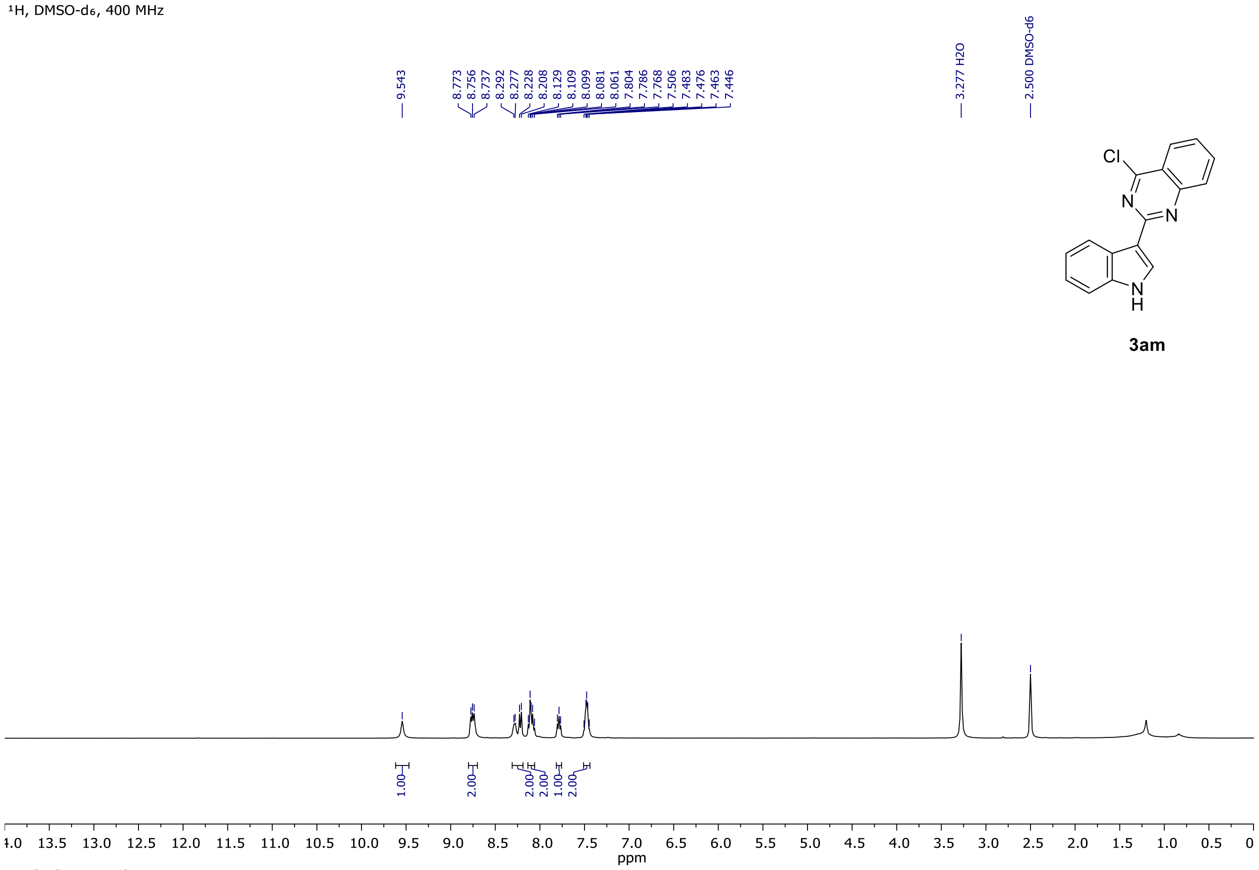

$^{13}C\{^1H\}$, DMSO-d₆, 100 MHz

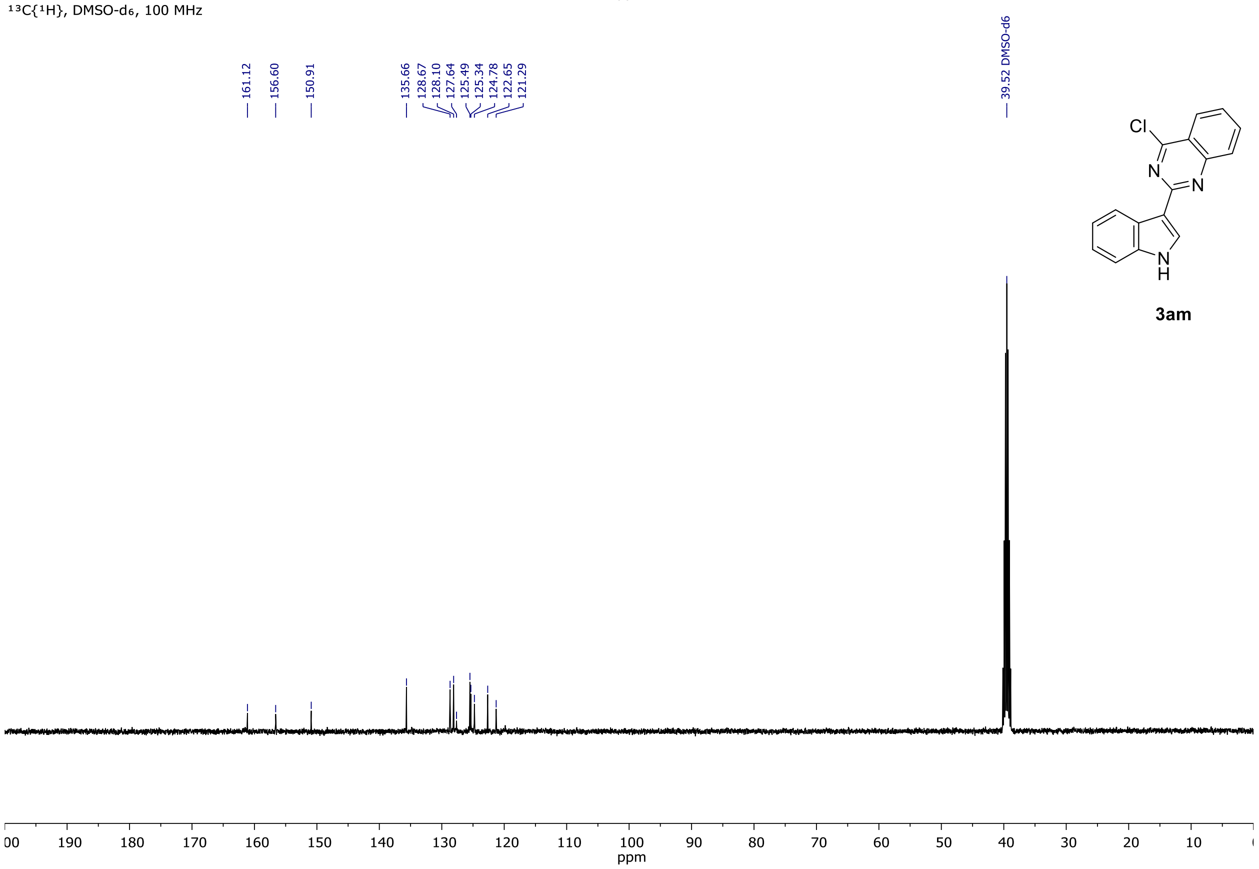

¹H, DMSO-d₆, 400 MHz

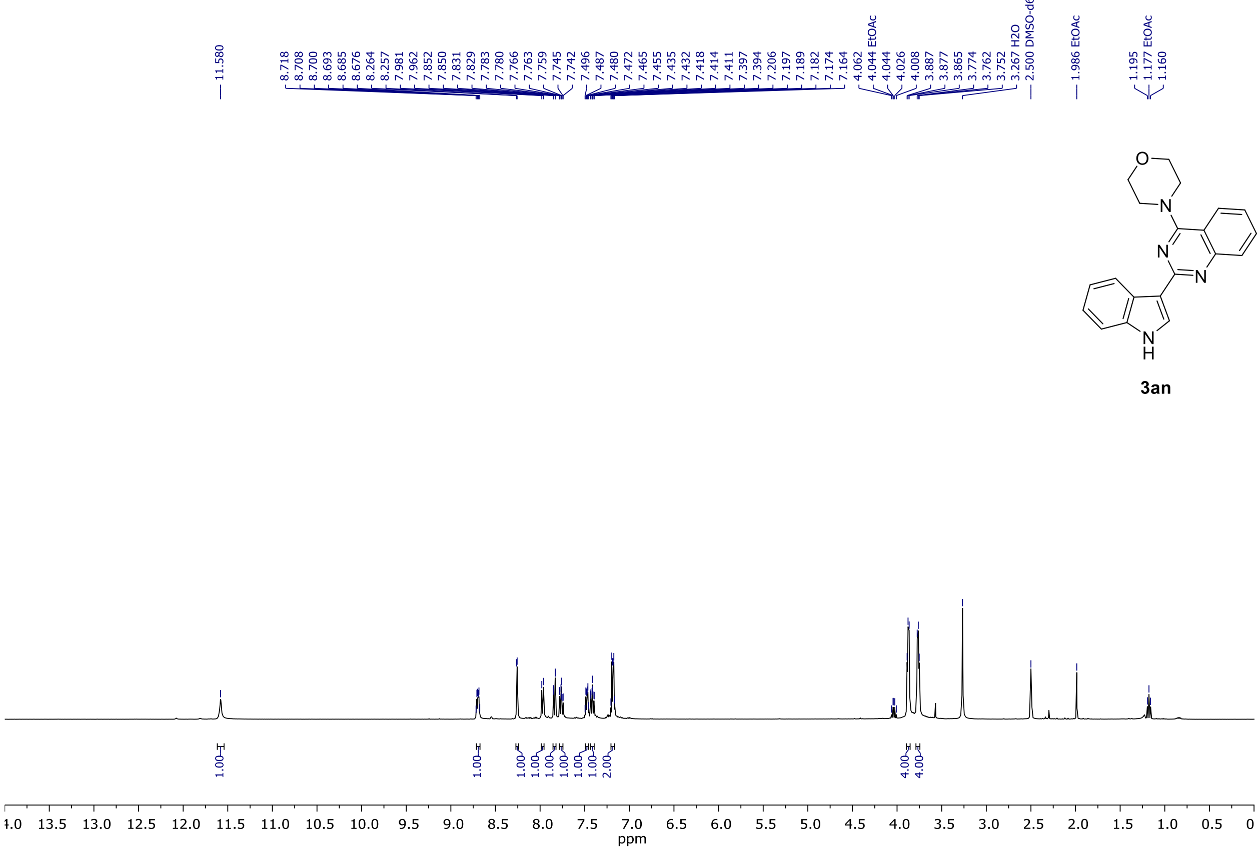

¹³C{¹H}, DMSO-d₆, 100 MHz

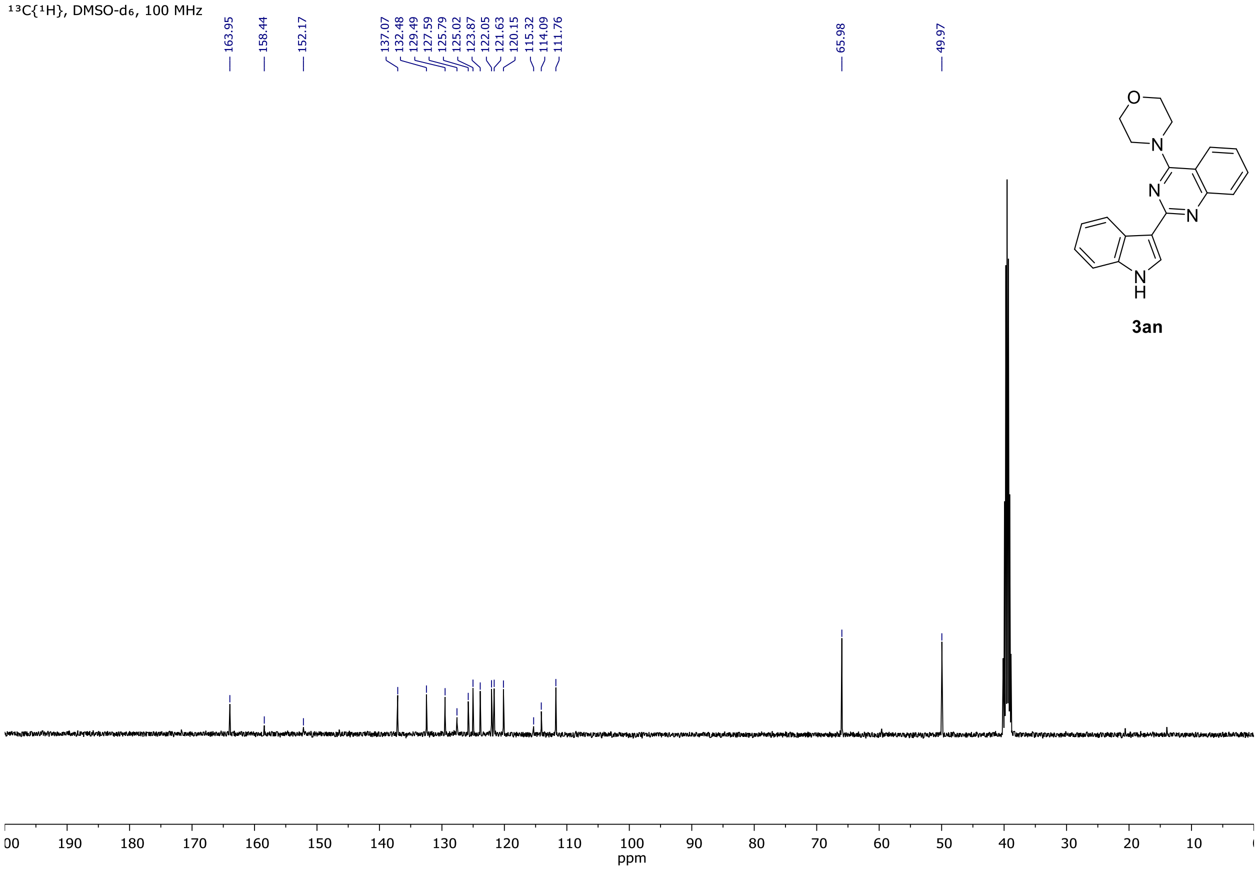

4. Copies of HRMS of new compounds

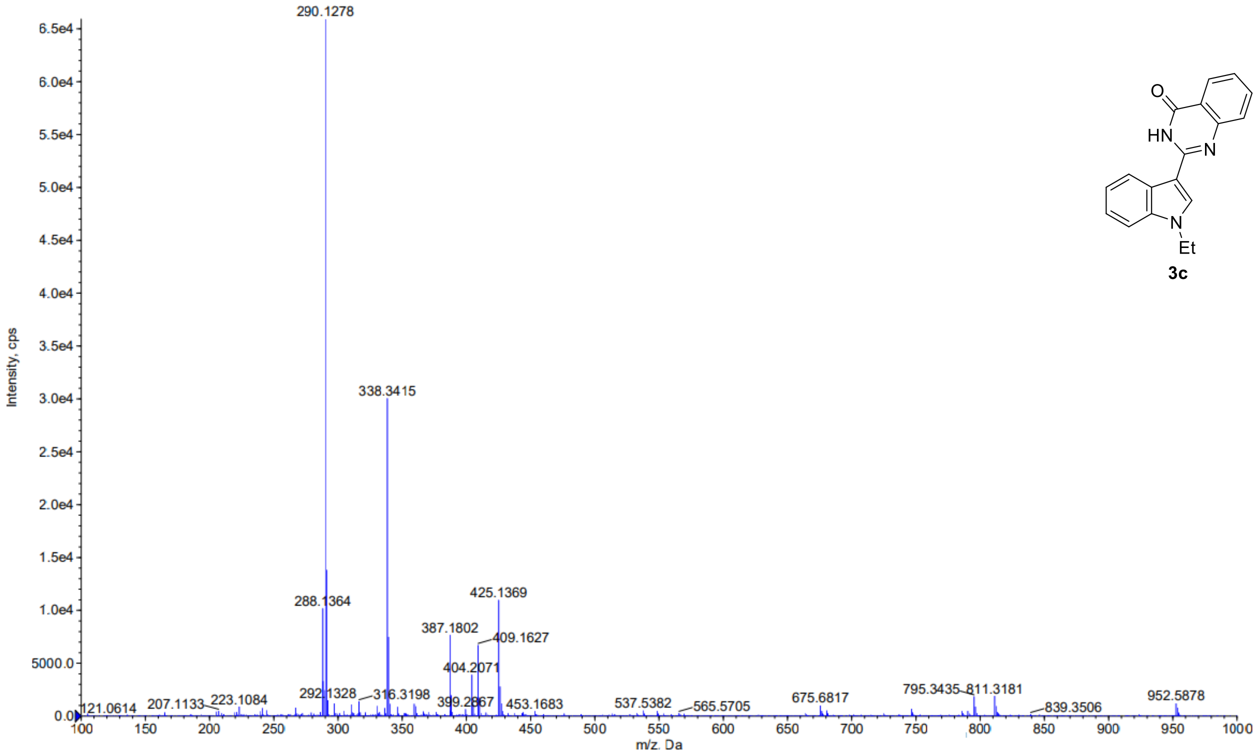

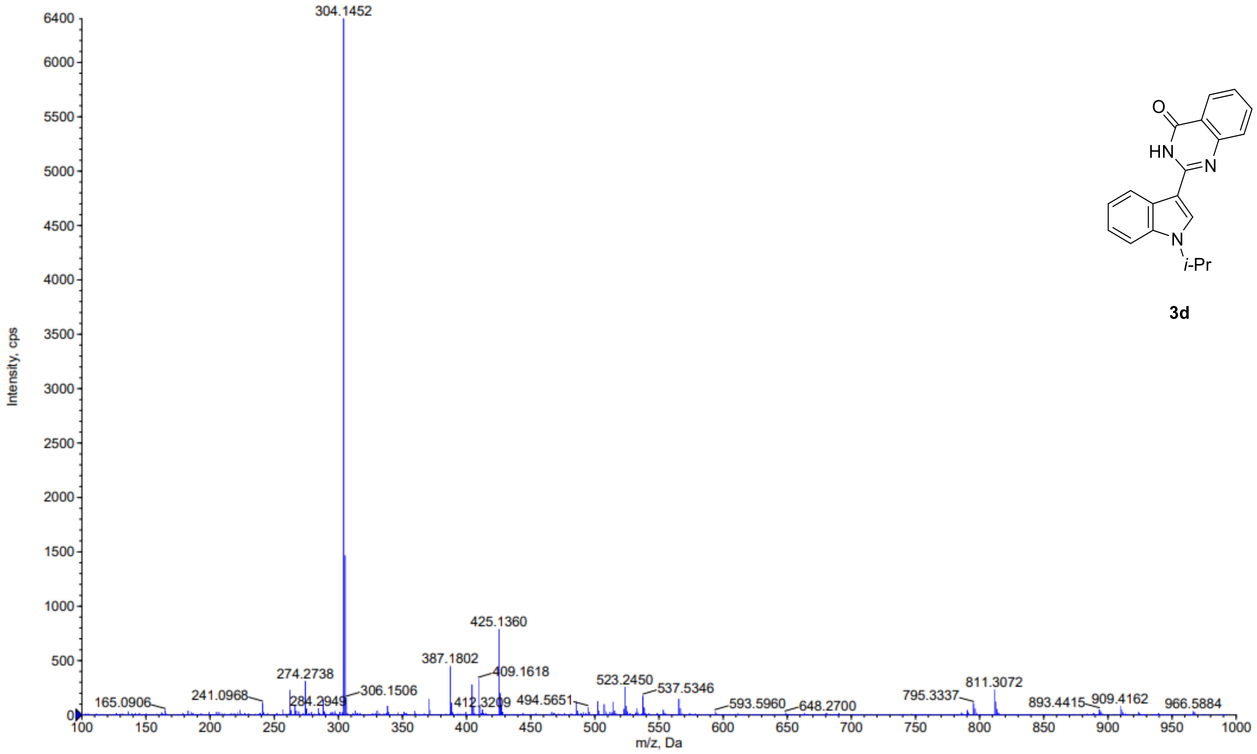

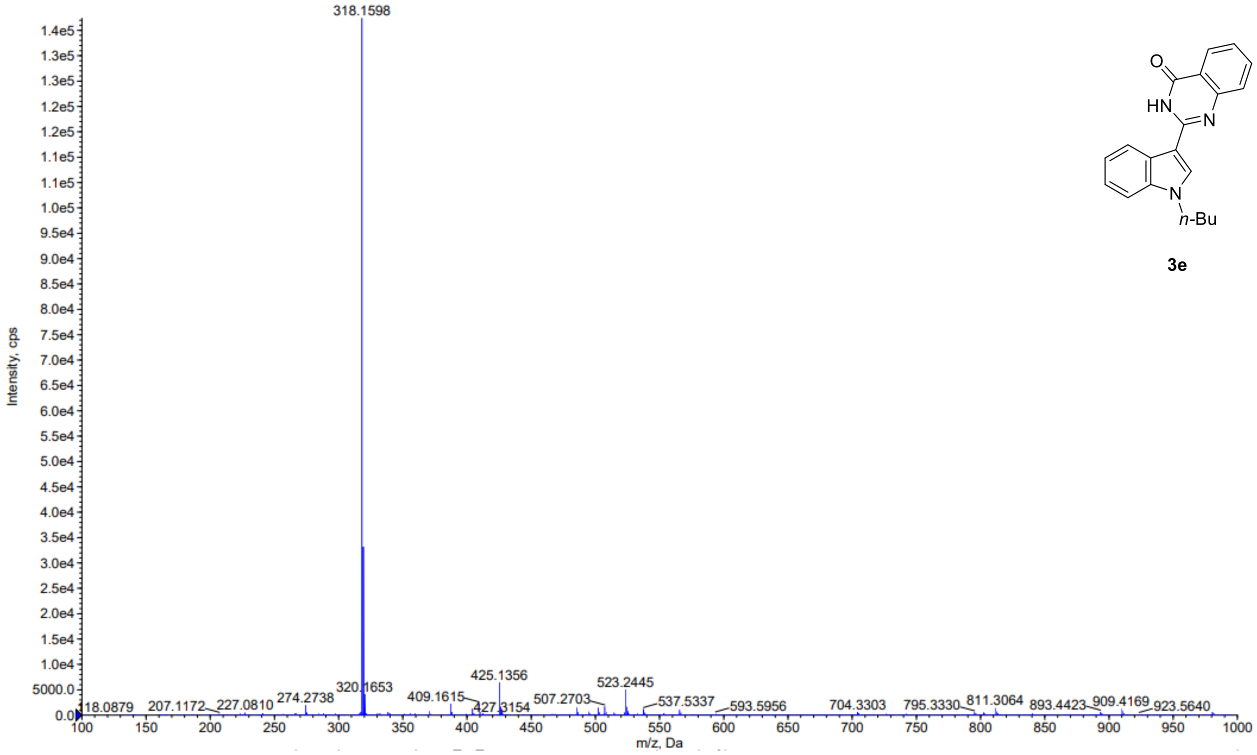
318.1598
Intensity, cps
m/z, Da
425.1356
523.2445
320.1653
HN
N
O
N
n-Bu

3e

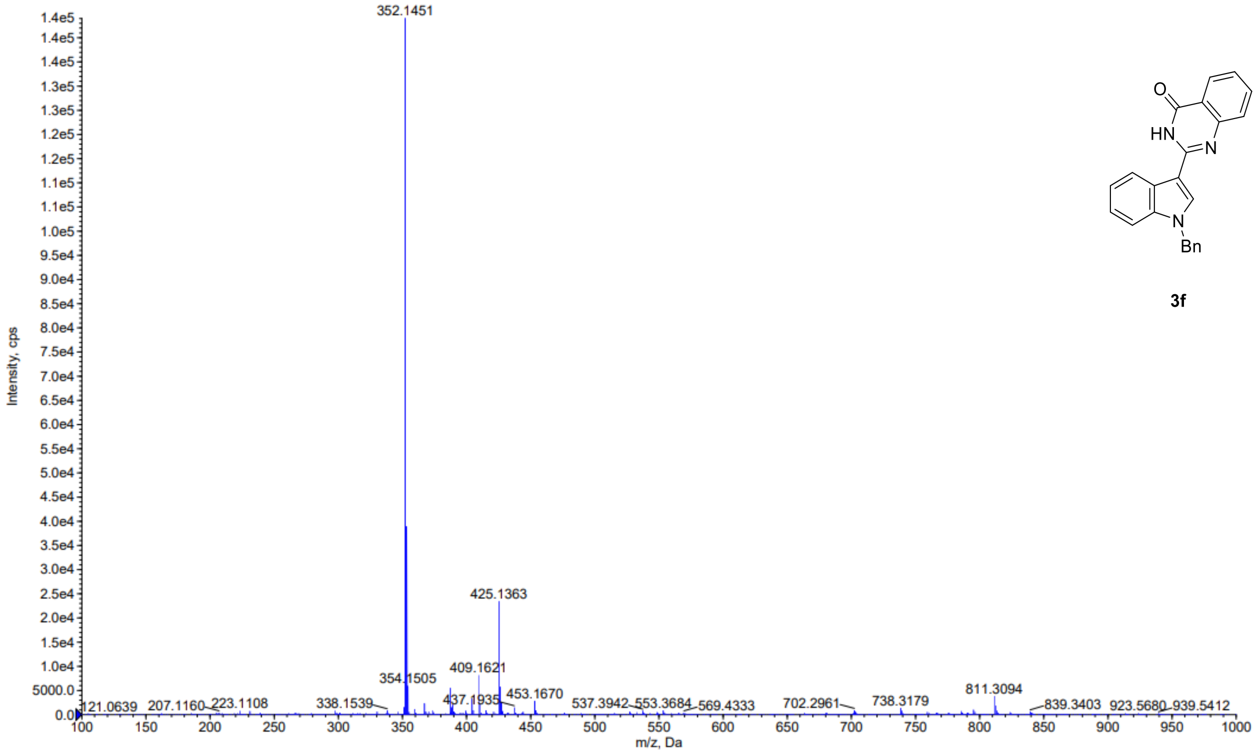
352.1451
Intensity, cps
m/z, Da
425.1363
409.1621
354.1505
811.3094
HN
N
O
N
Bn

3f

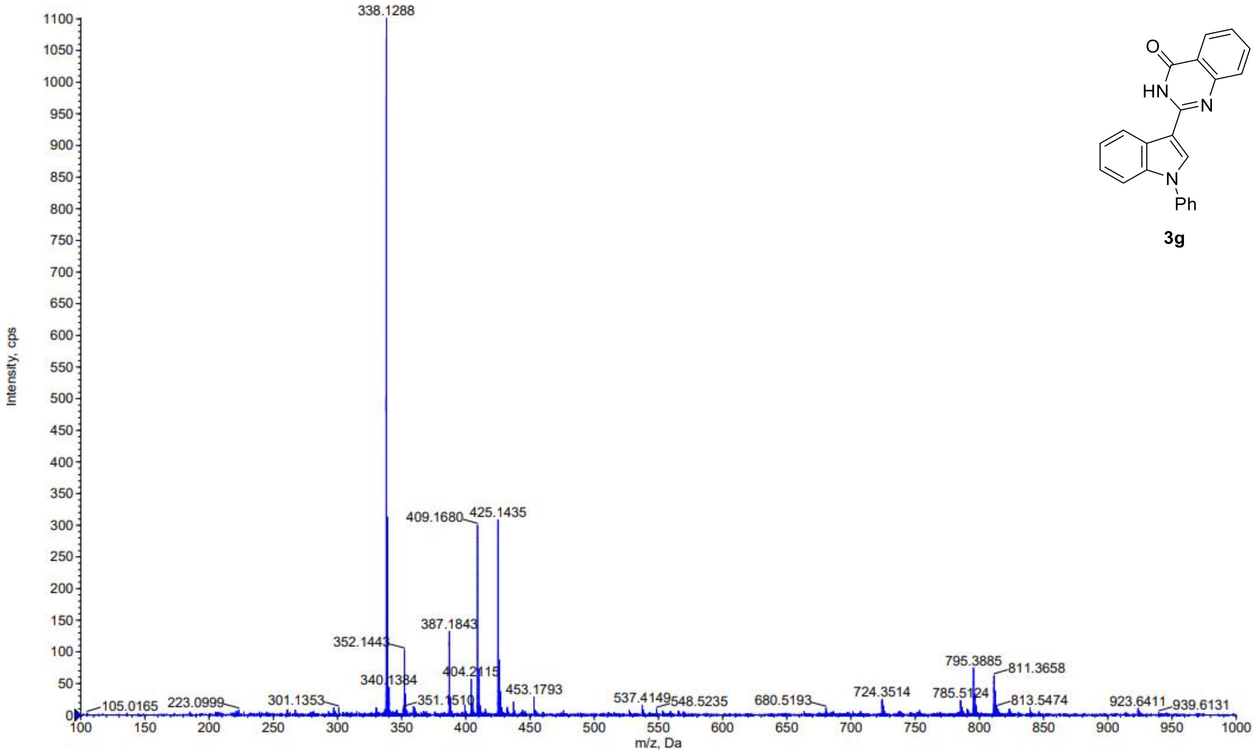
Intensity, cps
m/z, Da
338.1288
409.1680
425.1435
387.1843
352.1443
404.2115
340.1384
453.1793
351.1510
105.0165
223.0999
301.1353
537.4149
548.5235
680.5193
724.3514
795.3885
811.3658
785.5124
813.5474
923.6411
939.6131
O
HN
N
N
Ph

3g

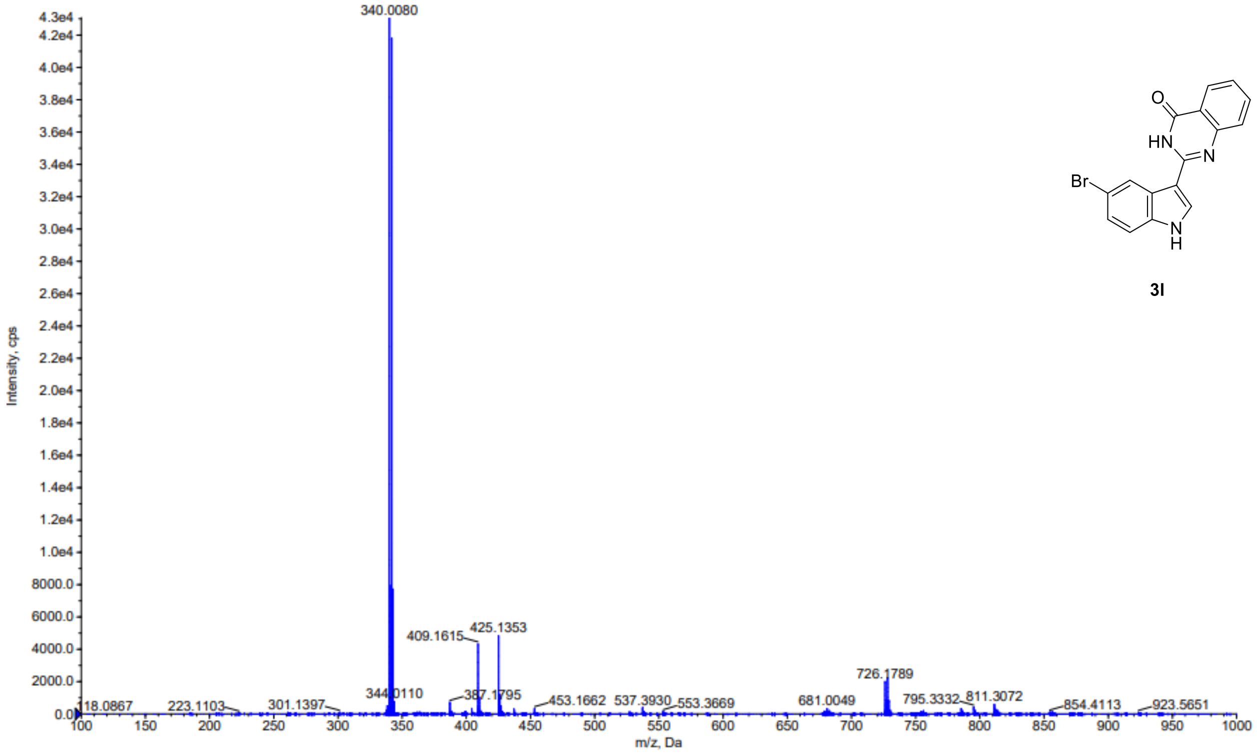
Intensity, cps
m/z, Da
340.0080
409.1615
425.1353
344.0110
387.1795
453.1662
537.3930
553.3669
681.0049
726.1789
795.3332
811.3072
854.4113
923.5651
118.0867
223.1103
301.1397
O
HN
N
Br
N
H

3l

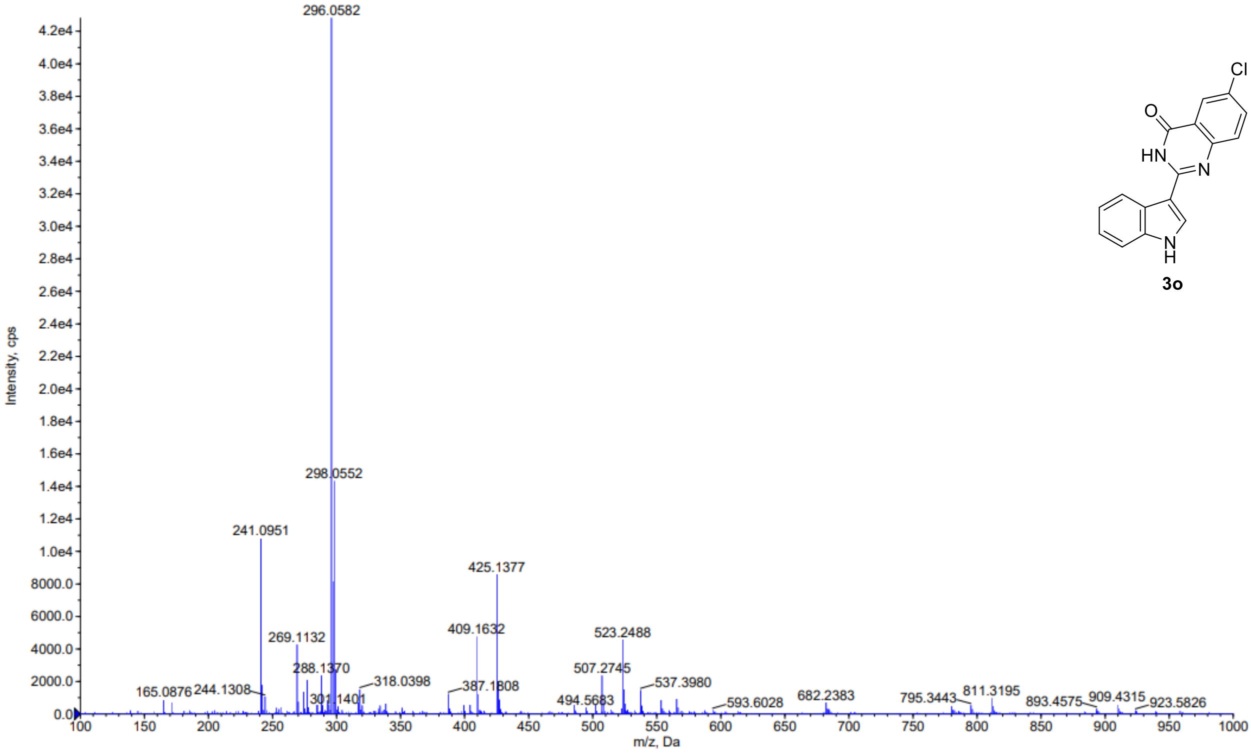

Intensity, cps
m/z, Da
296.0582
298.0552
241.0951
425.1377
409.1632
269.1132
523.2488
288.1370
507.2745
318.0398
537.3980
165.0876
244.1308
301.1401
387.1808
494.5683
593.6028
682.2383
795.3443
811.3195
893.4575
909.4315
923.5826
Cl
O
HN
N
N
H

3o

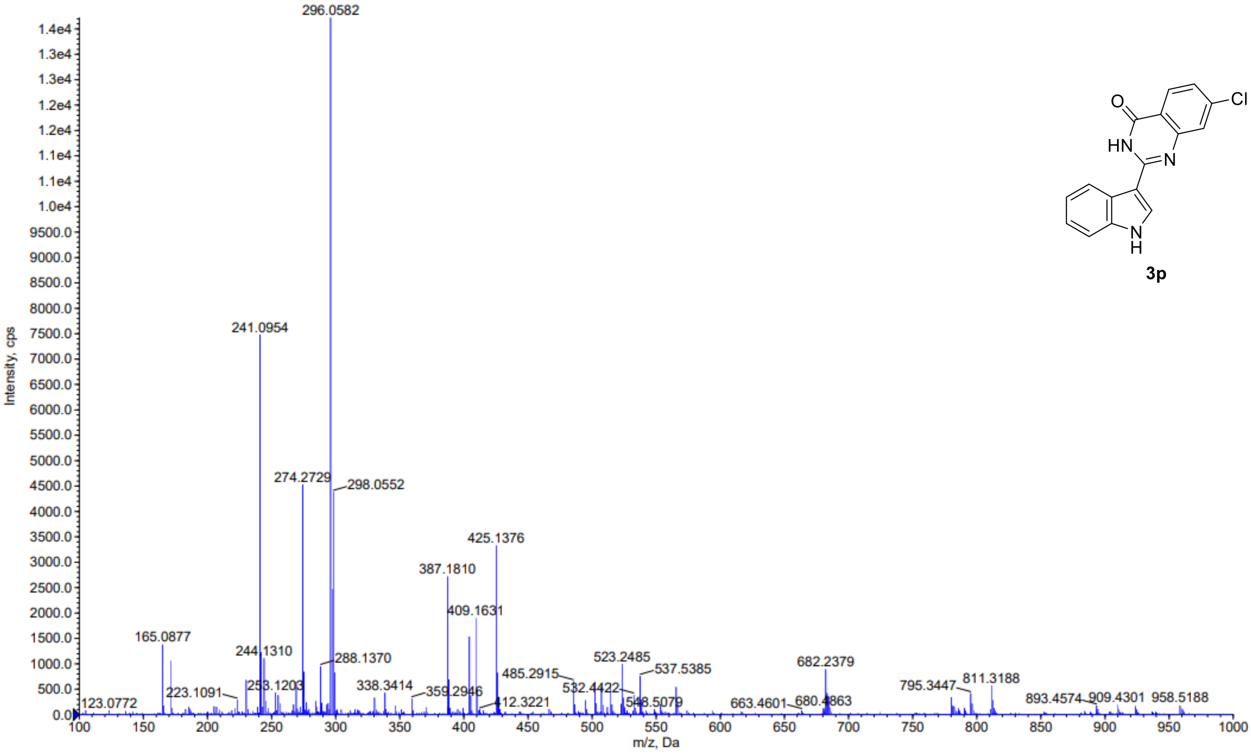

Intensity, cps
m/z, Da
296.0582
241.0954
274.2729
298.0552
425.1376
387.1810
409.1631
165.0877
244.1310
288.1370
523.2485
485.2915
537.5385
682.2379
532.4422
338.3414
223.1091
253.1203
359.2946
412.3221
548.5079
663.4601
680.4863
795.3447
811.3188
893.4574
909.4301
958.5188
123.0772
O
Cl
HN
N
N
H

3p

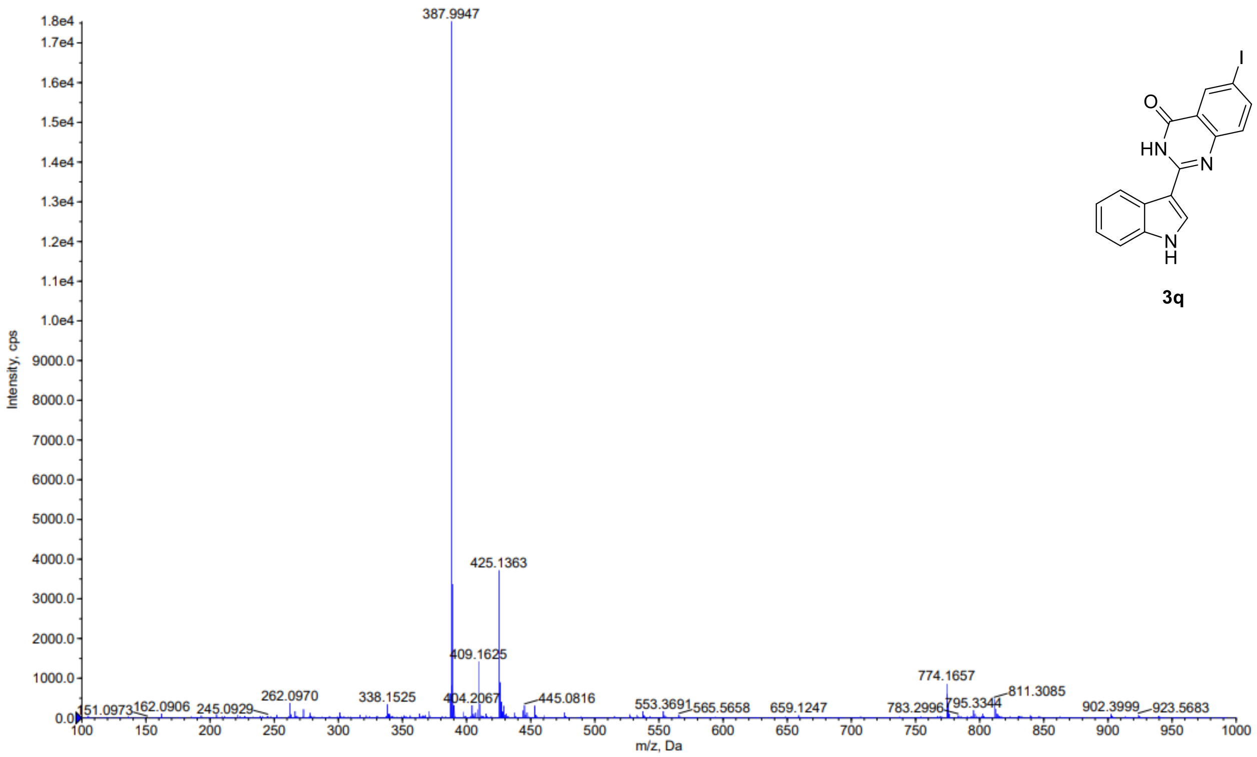
Intensity, cps
m/z, Da
387.9947
425.1363
409.1625
774.1657
811.3085
O
HN
N
I
N
H

3q

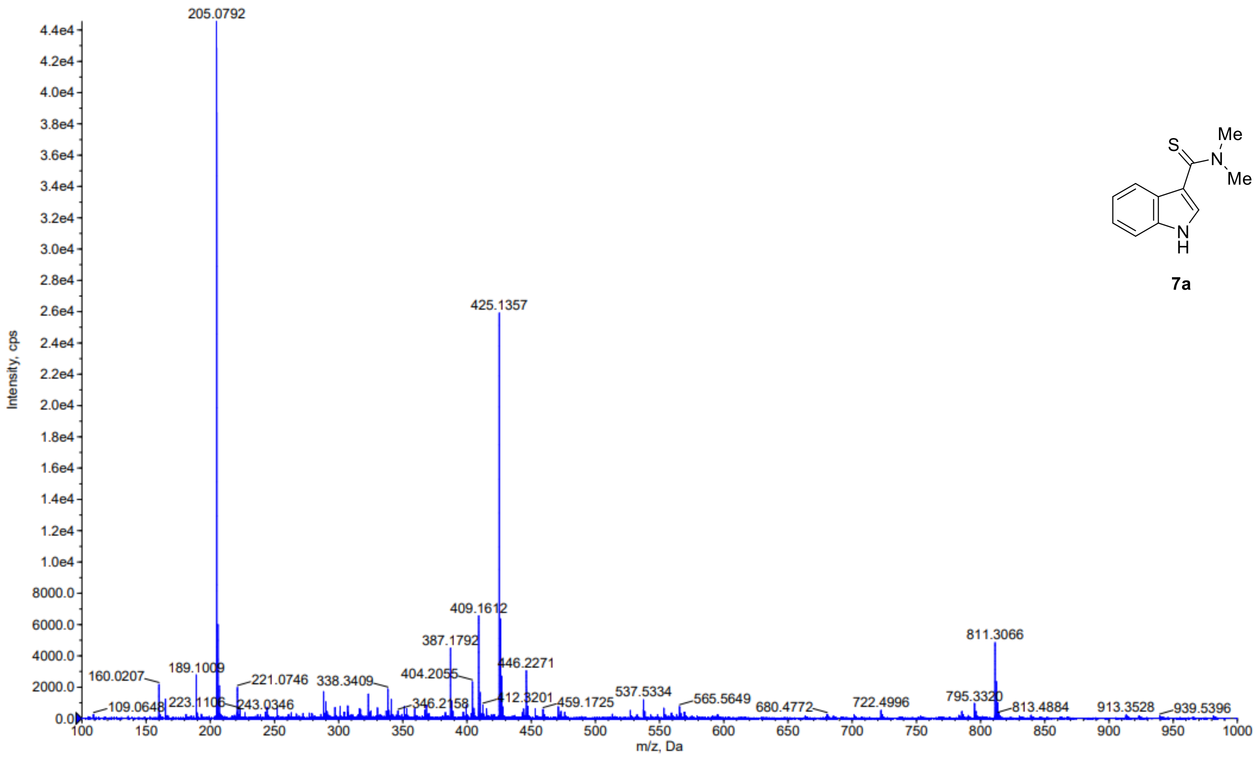
Intensity, cps
m/z, Da
205.0792
425.1357
409.1612
387.1792
811.3066
446.2271
S
N
Me
Me
N
H

7a

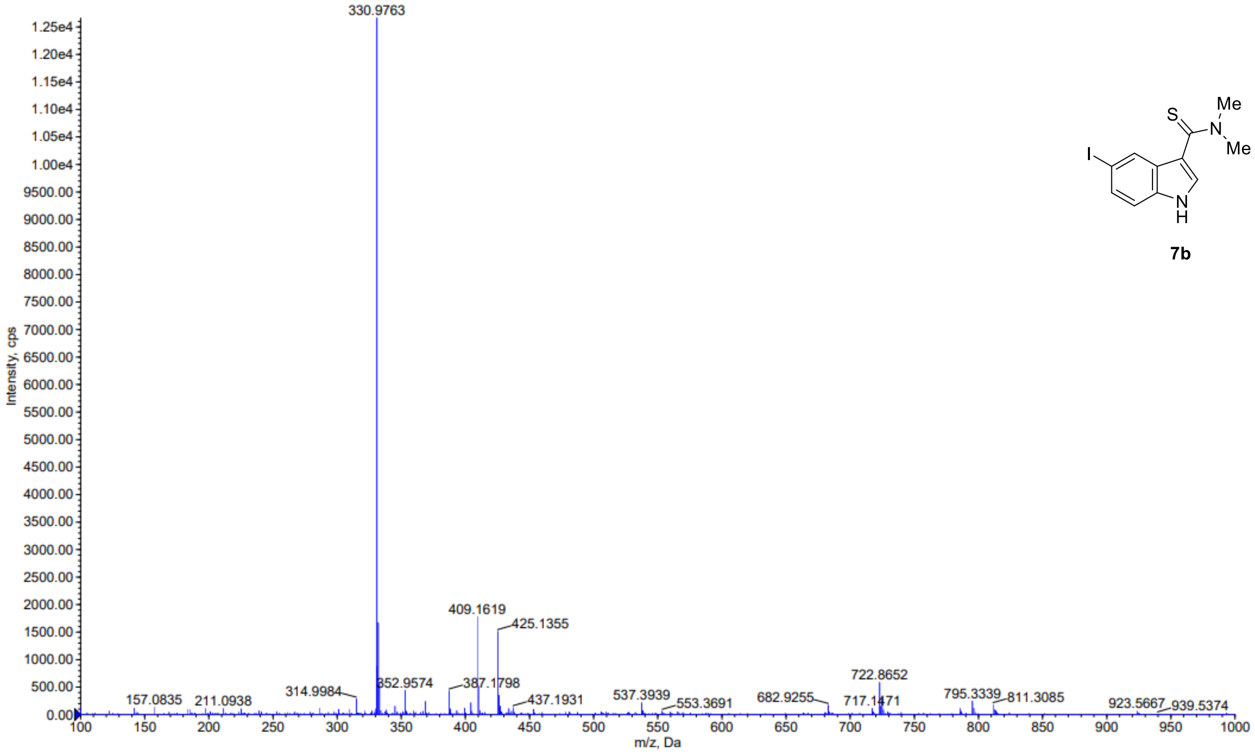
330.9763
409.1619
425.1355
157.0835
211.0938
314.9984
352.9574
387.1798
437.1931
537.3939
553.3691
682.9255
722.8652
717.1471
795.3339
811.3085
923.5667
939.5374
Intensity, cps
m/z, Da
S
N
Me
Me
I
N
H

7b

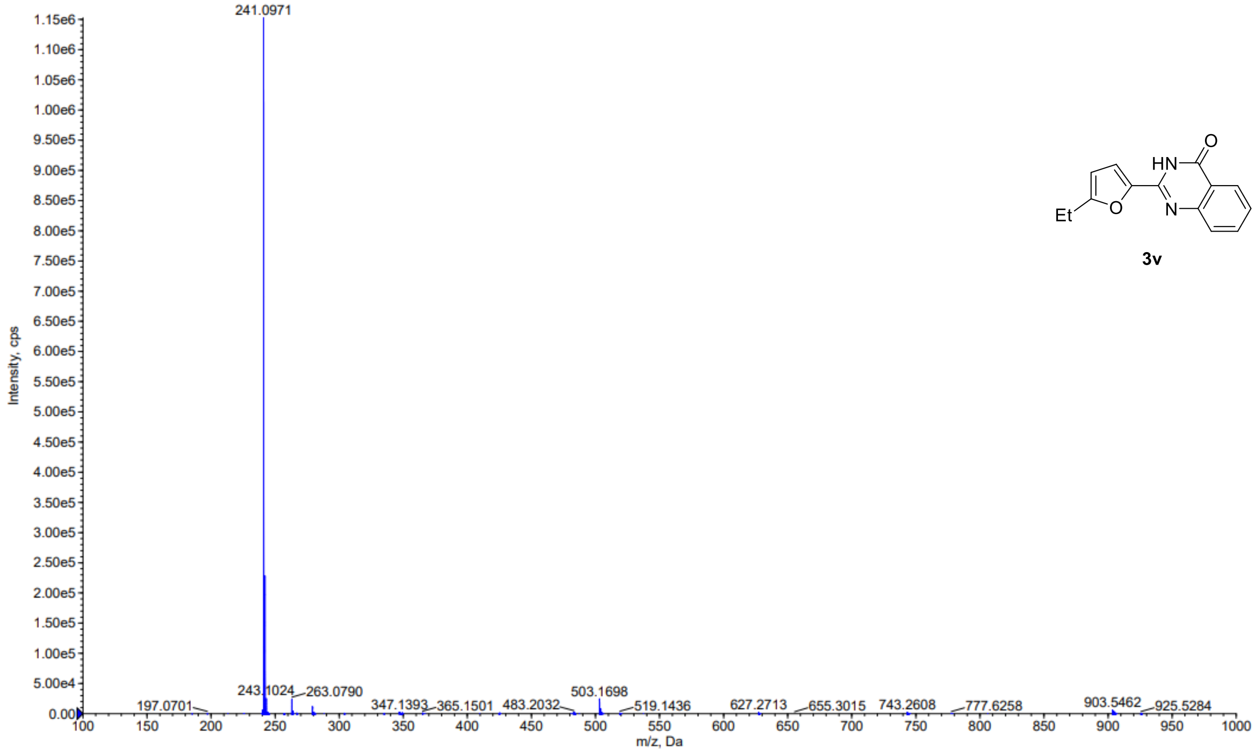
241.0971
243.1024
263.0790
197.0701
347.1393
365.1501
483.2032
503.1698
519.1436
627.2713
655.3015
743.2608
777.6258
903.5462
925.5284
Intensity, cps
m/z, Da
O
HN
N
O
Et

3v

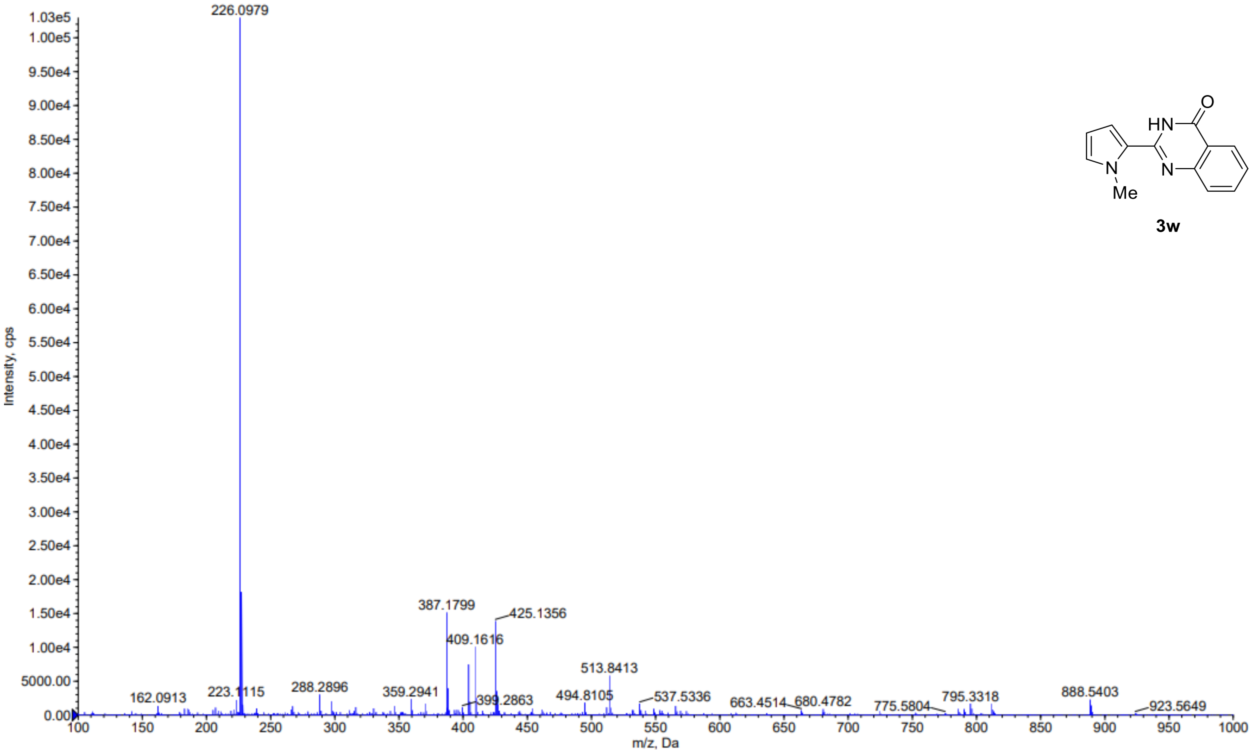
226.0979
387.1799
425.1356
409.1616
513.8413
162.0913
223.1115
288.2896
359.2941
399.2863
494.8105
537.5336
663.4514
680.4782
775.5804
795.3318
888.5403
923.5649
Intensity, cps
m/z, Da
HN
O
N
N
Me

3w

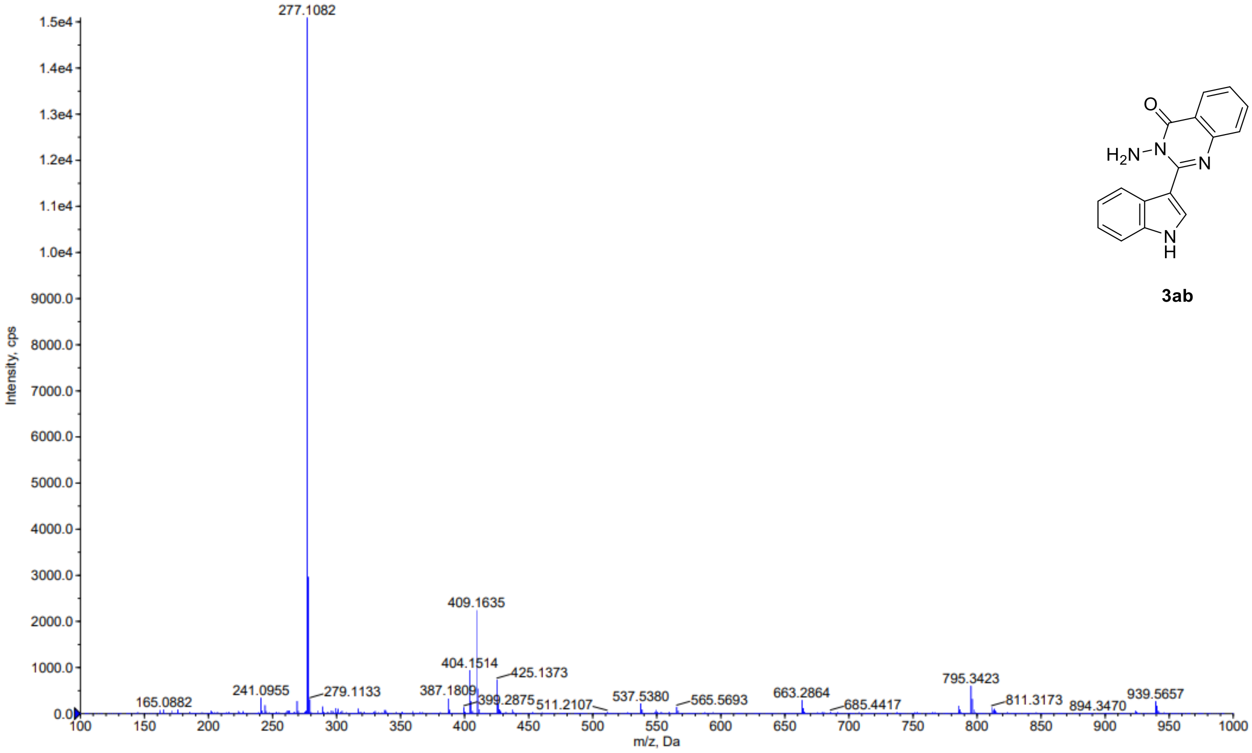
277.1082
409.1635
404.1514
425.1373
165.0882
241.0955
279.1133
387.1809
399.2875
511.2107
537.5380
565.5693
663.2864
685.4417
795.3423
811.3173
894.3470
939.5657
Intensity, cps
m/z, Da
O
H2N
N
N
N
H

3ab

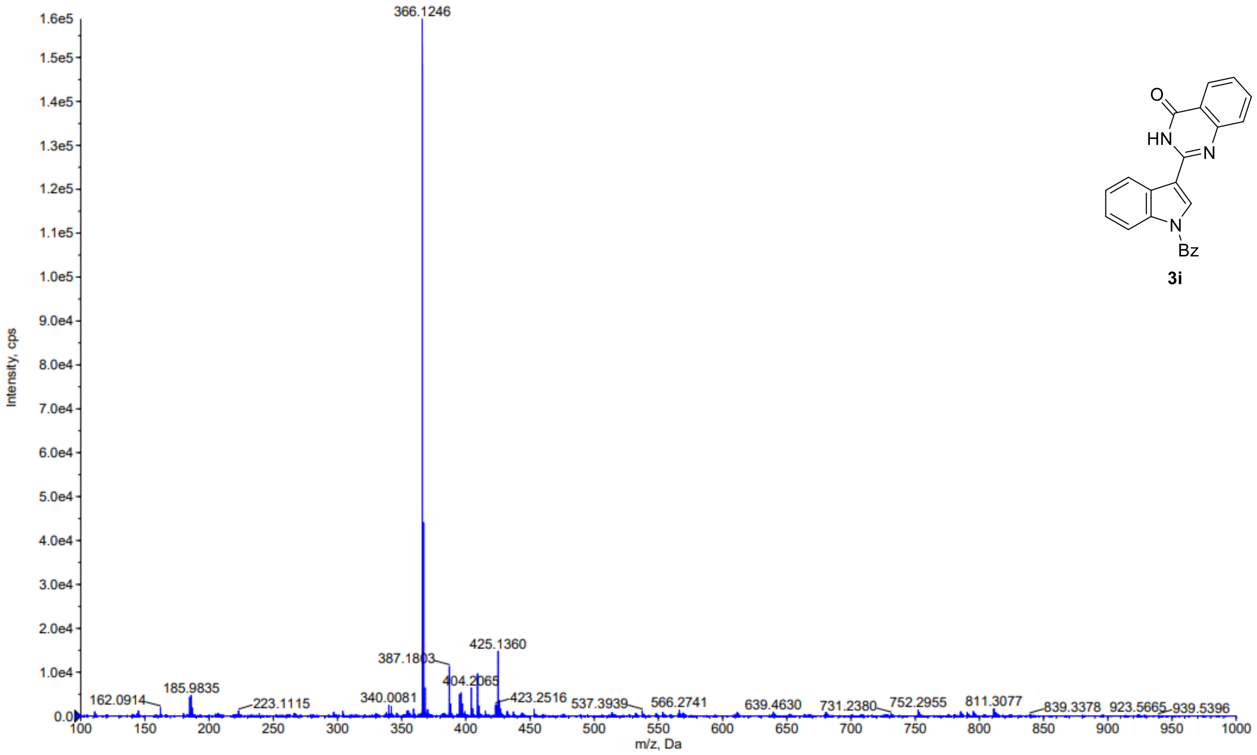
366.1246
425.1360
387.1803
404.2065
423.2516
185.9835
162.0914
223.1115
340.0081
537.3939
566.2741
639.4630
731.2380
752.2955
811.3077
839.3378
923.5665
939.5396
Intensity, cps
m/z, Da
O
HN
N
N
Bz
3i

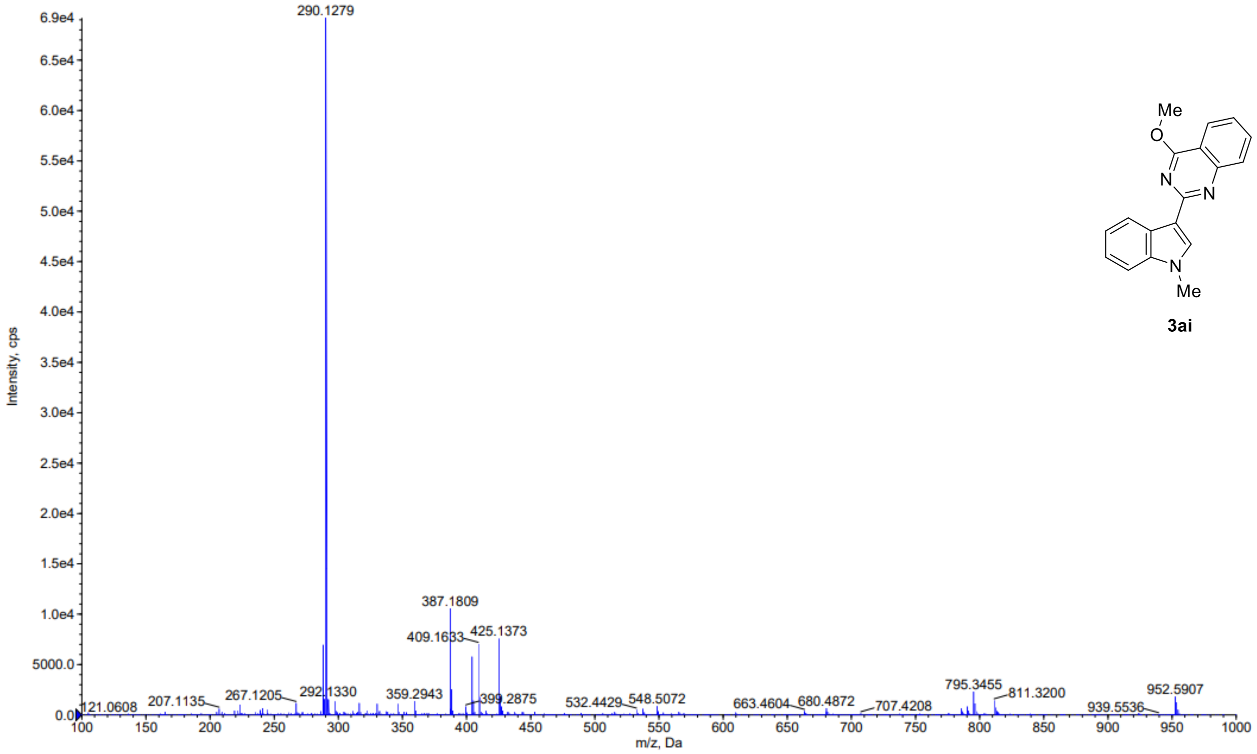
290.1279
387.1809
409.1633
425.1373
292.1330
359.2943
399.2875
121.0608
207.1135
267.1205
532.4429
548.5072
663.4604
680.4872
707.4208
795.3455
811.3200
939.5536
952.5907
Intensity, cps
m/z, Da
Me
O
N
N
N
Me
3ai

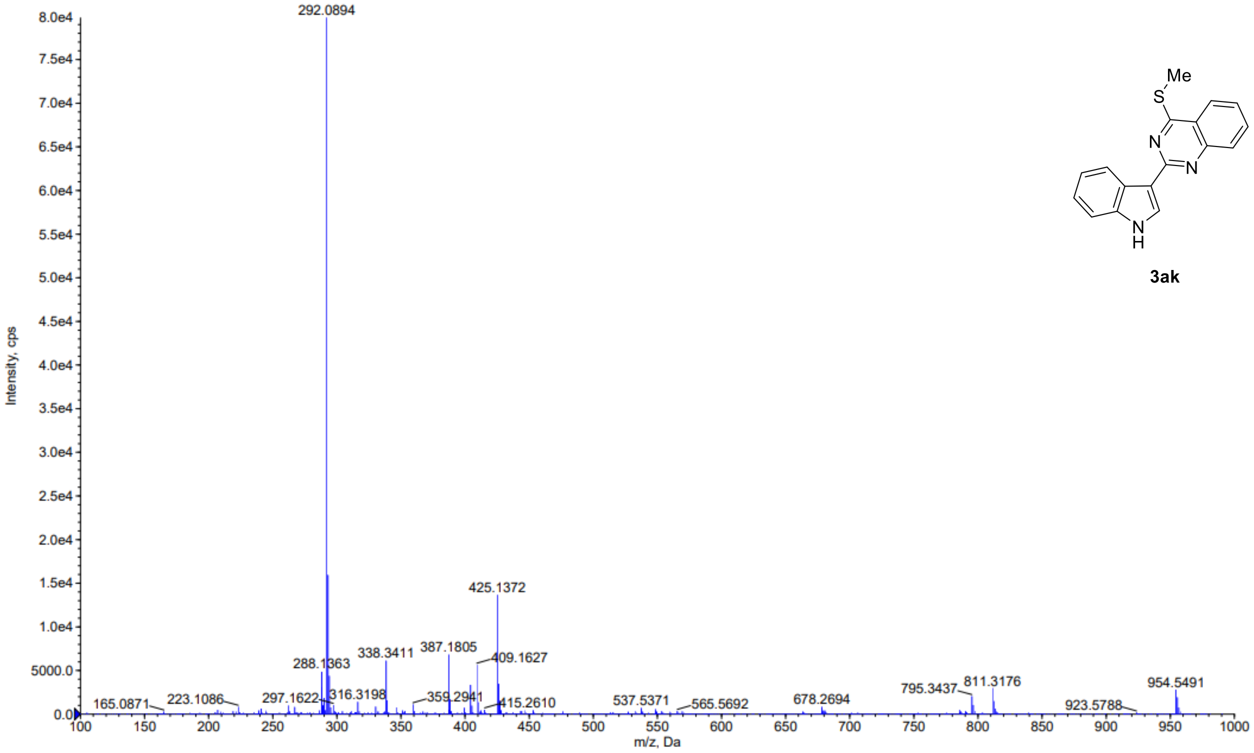

Intensity, cps
m/z, Da
292.0894
425.1372
338.3411
387.1805
409.1627
288.1363
316.3198
297.1622
359.2941
415.2610
165.0871
223.1086
537.5371
565.5692
678.2694
795.3437
811.3176
923.5788
954.5491
Me
S
N
N
N
H

3ak

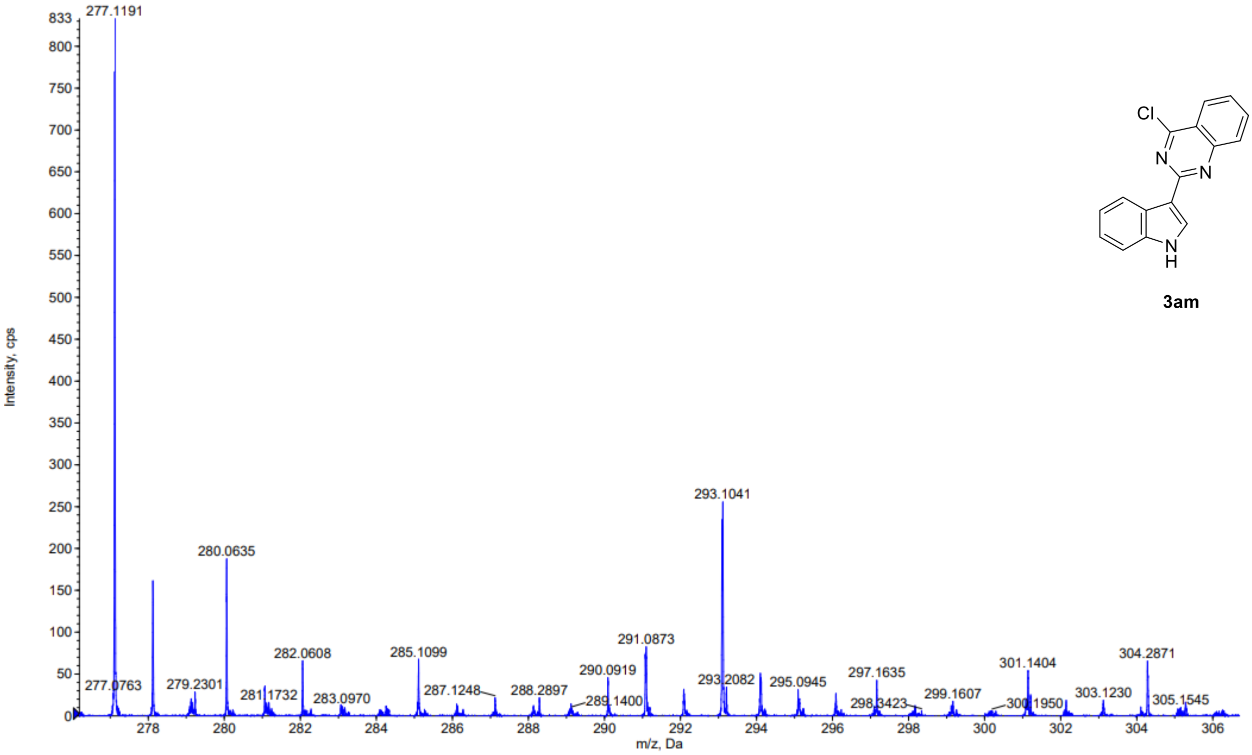

Intensity, cps
m/z, Da
277.1191
293.1041
280.0635
291.0873
282.0608
285.1099
290.0919
277.0763
279.2301
281.1732
283.0970
287.1248
288.2897
289.1400
293.2082
295.0945
297.1635
298.3423
299.1607
300.1950
301.1404
303.1230
304.2871
305.1545
Cl
N
N
N
H

3am

5. X-ray crystallography data

Table S2: Experimental details for *N*,*N*-dimethyl-1*H*-indole-3-carbothioamide (**7a**, CCDC 2269768).

Crystal data	
Chemical formula	$C_{11}H_{12}N_2S$
M_r	204.29
Crystal system, space group	Monoclinic, $P2_1/n$
Temperature (K)	295
a, *b*, *c* (Å)	7.3423 (14), 7.6336 (10), 18.673 (3)
β (°)	101.484 (18)
V (Å^3)	1025.6 (3)
Z	4
Radiation type	Mo *Kα*
μ (mm^{-1})	0.28
Crystal size (mm)	0.55 × 0.25 × 0.2
Data collection	
Diffractometer	New Xcalibur, Ruby
Absorption correction	Multi-scan<br>*CrysAlis PRO*, Agilent Technologies, Version 1.171.37.33 (release 27-03-2014 CrysAlis171 .NET) (compiled Mar 27 2014,17:12:48) Empirical absorption correction using spherical harmonics, implemented in SCALE3 ABSPACK scaling algorithm.
T_{min}, T_{max}	0.668, 1.000
No. of measured, independent and observed [$I > 2\sigma(I)$] reflections	5309, 2421, 2089
R_{int}	0.029
$(\sin \theta/\lambda)_{max}$ (Å^{-1})	0.691
Refinement	
$R[F^2 > 2\sigma(F^2)]$, $wR(F^2)$, S	0.039, 0.109, 1.05
No. of reflections	2421
No. of parameters	133
H-atom treatment	H atoms treated by a mixture of independent and constrained refinement
$\Delta\rho_{max}$, $\Delta\rho_{min}$ (e Å^{-3})	0.33, −0.24

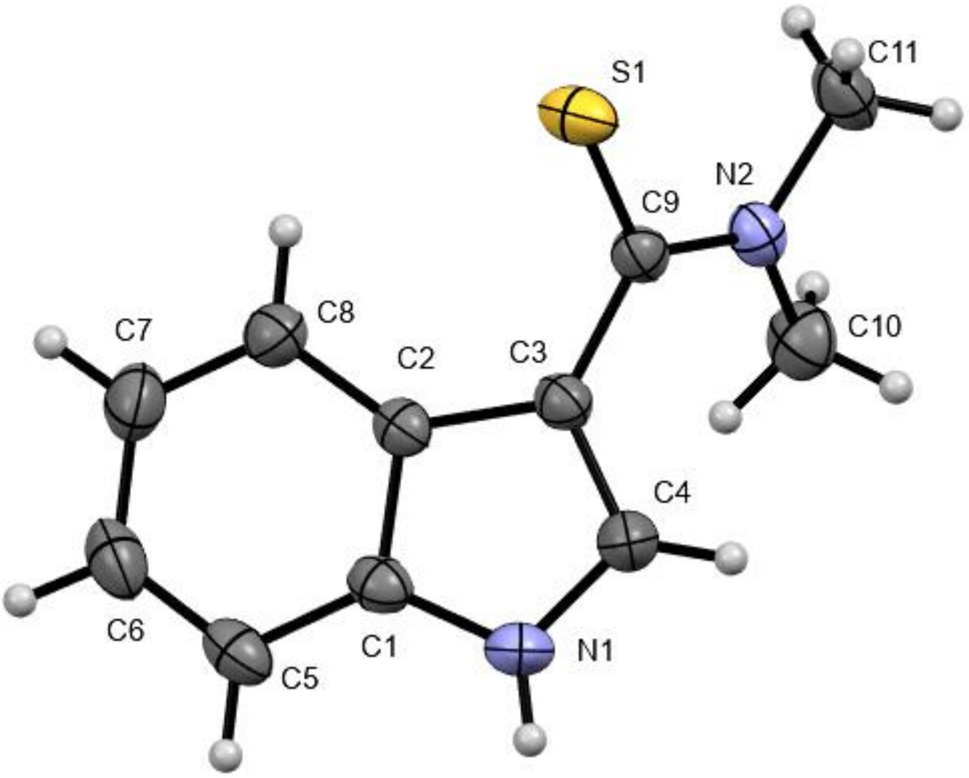

Figure S1. Structure of the *N*,*N*-dimethyl-1*H*-indole-3-carbothioamide (**7a**, CCDC 2269768) according to the X-ray diffraction data; non-hydrogen atoms are shown as thermal vibration ellipsoids with a probability of 50%.
